# Isobactins: *O*-acyl isopeptide prodrugs of teixobactin and teixobactin derivatives†

**DOI:** 10.1039/d2sc02670h

**Published:** 2022-10-24

**Authors:** Chelsea R. Jones, Gretchen Guaglianone, Grant H. Lai, James S. Nowick

**Affiliations:** Department of Chemistry, University of California, Irvine Irvine California 92697 USA jsnowick@uci.edu; Department of Pharmaceutical Sciences, University of California, Irvine Irvine California 92697 USA

## Abstract

The antibiotic teixobactin is a promising drug candidate against drug-resistant pathogens, such as MRSA and VRE, but forms insoluble gels that may limit intravenous administration. *O*-Acyl isopeptide prodrug analogues of teixobactin circumvent the problem of gel formation while retaining antibiotic activity. The teixobactin prodrug analogues contain ester linkages between Ile_6_ and Ser_7_, Ile_2_ and Ser_3_, or between both Ile_6_ and Ser_7_ and Ile_2_ and Ser_3_. Upon exposure to physiological pH, the prodrug analogues undergo clean conversion to the corresponding amides, with half-lives between 13 and 115 min. Prodrug analogues containing lysine, arginine, or leucine at position 10 exhibit good antibiotic activity against a variety of Gram-positive bacteria while exhibiting little or no cytotoxicity or hemolytic activity. Because *O*-acyl isopeptide prodrug analogues of teixobactin exhibit clean conversion to the corresponding teixobactin analogues with reduced propensity to form gels, it is anticipated that teixobactin prodrugs will be superior to teixobactin as drug candidates.

## Abstract 2

*O*-Acyl isopeptide prodrug analogues of the antibiotic teixobactin are stable and non-gelating at acidic pH but convert to the corresponding active teixobactin analogues at neutral pH, thus overcoming limitations in intravenous dosing of teixobactin.

## Graphical abstract

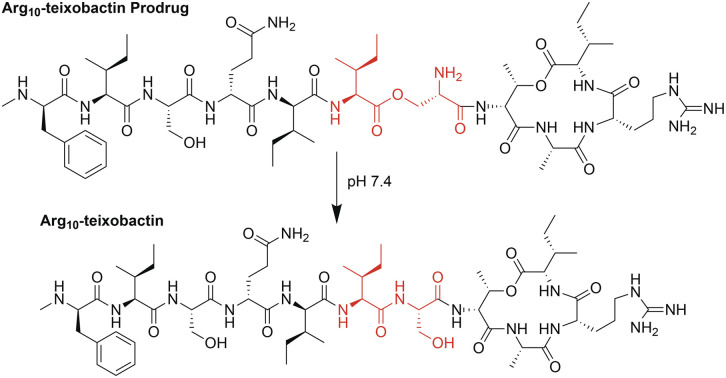

## Introduction

**Fig. 1:**
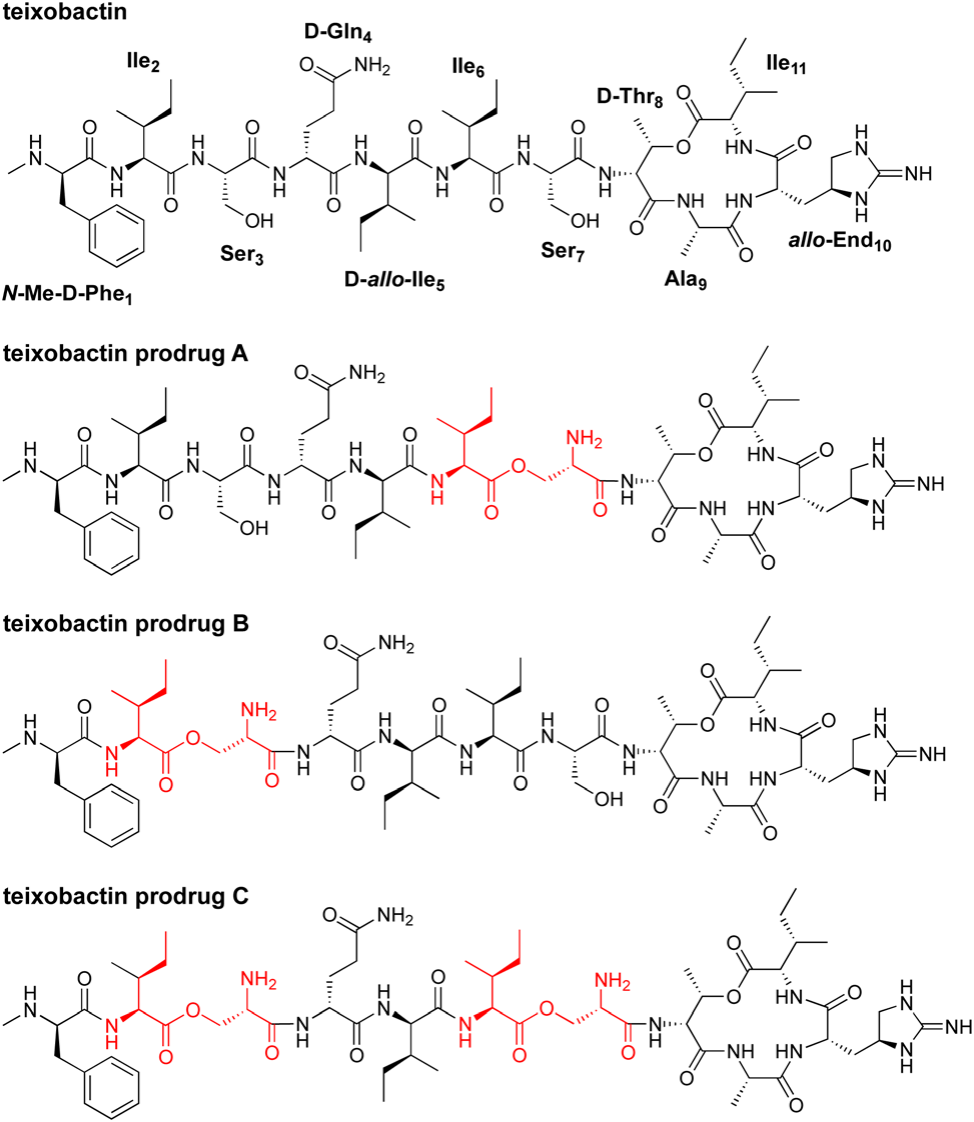
Structures of teixobactin and teixobactin prodrugs.

In 2015, teixobactin was reported as a promising new antibiotic candidate.^1^ This non-ribosomal depsipeptide has excellent antibiotic activity against Gram-positive bacteria, including drug-resistant strains such as MRSA, VRE, and MDR-TB (Fig. 1). The macrocyclic ring of teixobactin binds to the highly conserved prenyl-pyrophosphate-saccharide regions of lipid II and related membrane-bound cell wall precursors.^1–5^ These targets are extracellular and immutable, making it almost impossible for bacteria to develop resistance.^3,6–8^ For these reasons, teixobactin has generated considerable excitement as a promising new candidate for antibiotic drug development.

Unfortunately, teixobactin aggregates to form gels in the physiological conditions needed for intravenous administration. In performing structure–activity relationship studies with teixobactin analogues, our laboratory found that modifications of teixobactin that eliminated the propensity to form gels also eliminated its potent antibiotic activity.^9–11^ We further found that teixobactin and active teixobactin analogues assemble to form amyloid-like fibrils and that this mode of supramolecular assembly appears to be inherently connected to both the potent antibiotic activity and the formation of gels.^2,12^ The propensity of teixobactin to form gels has the potential to jeopardize its promise as a clinically useful intravenous antibiotic against drug-resistant Gram-positive pathogens by limiting dosing to low concentrations that do not form gels or aggregates.

**Fig. 2:**
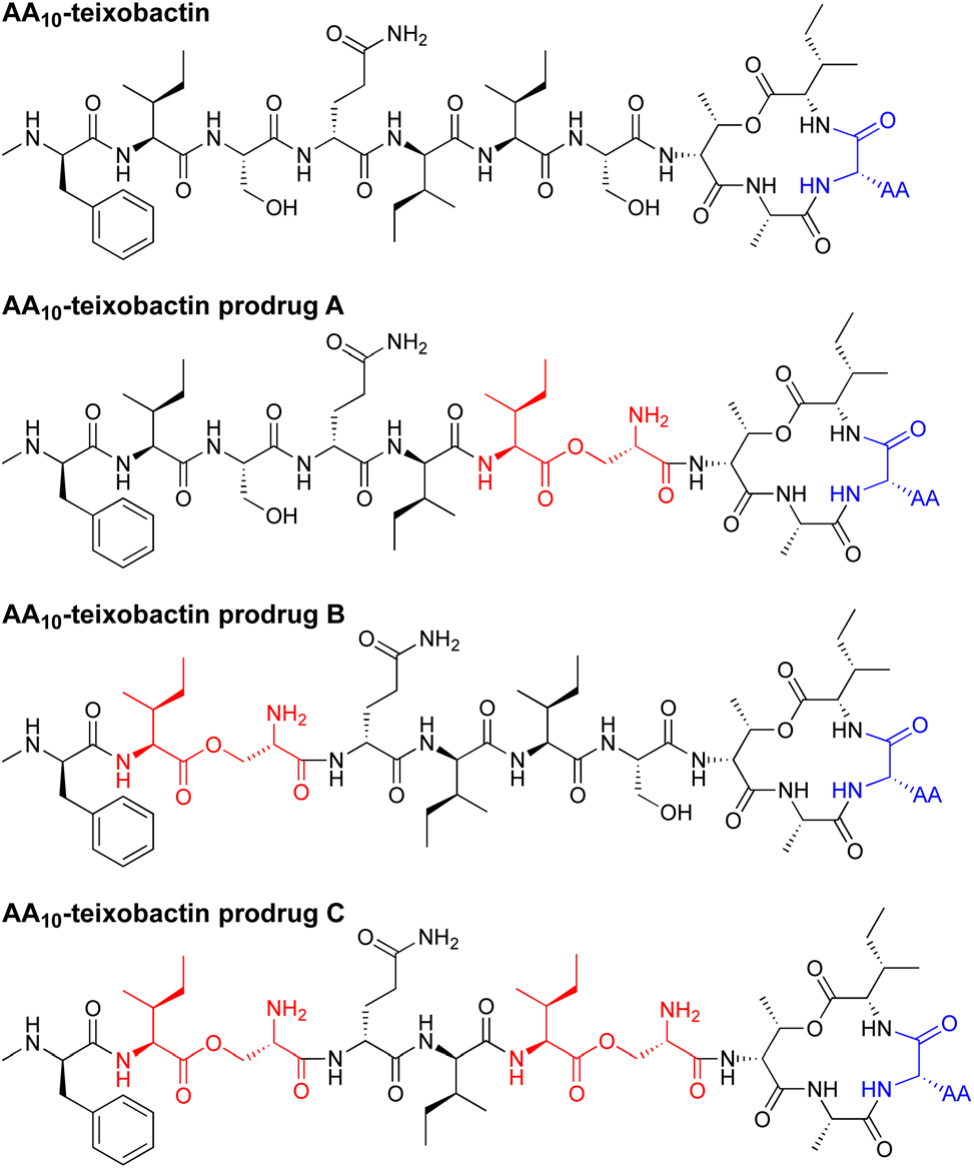
Structures of teixobactin analogues and *O*-acyl isopeptide prodrugs of teixobactin analogues. AA is lysine, arginine, or leucine.

In the current paper, we introduce *O*-acyl isopeptide prodrug analogues of teixobactin that are stable and non-gelating in acidic solution but gradually convert to the corresponding active teixobactin analogue at neutral pH (Fig. 2). We envision that prodrugs of native teixobactin (Fig. 1) and teixobactin prodrug analogues should thus circumvent the problem of gel formation and facilitate intravenous administration. The *O*-acyl isopeptide prodrug analogues undergo clean conversion to the corresponding teixobactin analogues at physiological pH. The prodrug analogues exhibit comparable or slightly improved antibiotic activity to the corresponding teixobactin analogues. The prodrug analogues also exhibit improved solubility in aqueous conditions and do not gelate immediately upon exposure to physiological conditions. Hemolytic assays with human red blood cells show little to no hemolytic activity, and cytotoxicity assays with HeLa cells show no significant cytotoxicity. A mouse thigh infection model against MRSA demonstrates *in vivo* efficacy. These findings suggest that teixobactin prodrugs and teixobactin prodrug analogues may be attractive alternatives to teixobactin as antibiotic drug candidates that circumvent the gelation problem of teixobactin.

## Results and discussion

**Fig. 3:**
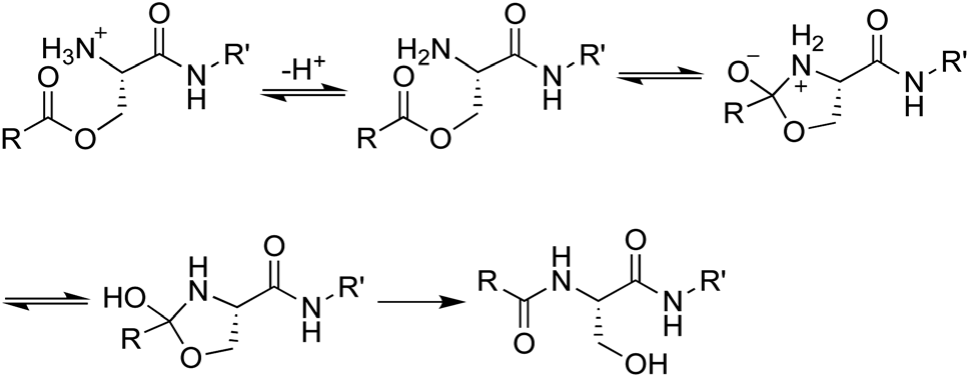
Mechanism of the conversion of *O*-acyl isopeptides to peptides.

We envisioned that the *O*-acyl isopeptide strategy that Kiso and coworkers developed to facilitate the synthesis of aggregation-prone peptide sequences could be adapted to circumvent the propensity of teixobactin to aggregate under conditions needed for intravenous administration.^13–17^ In the *O*-acyl isopeptide strategy, a native amide bond is replaced with an ester linkage to serine or threonine to disrupt β-sheet formation by an aggregation-prone sequence. The resulting *O*-acyl isopeptide also has an additional positive charge which further enhances solubility. Upon exposure to neutral or basic conditions, the *O*-acyl isopeptide rearranges to form the native amide bond (Fig. 3).

### Synthesis of teixobactin *O*-acyl isopeptide prodrug analogues

We adapted the synthesis of teixobactin analogues that our laboratory previously developed to permit the synthesis of *O*-acyl isopeptide prodrugs of teixobactin analogues.^11^ We synthesized the teixobactin *O*-acyl isopeptide prodrug analogues by Fmoc-based solid-phase peptide synthesis (SPPS) using the commercially available Boc-Ser(Fmoc-Ile)-OH *O*-acyl isodipeptide building block in place of Ile_2_ and Ser_3_, Ile_6_ and Ser_7_, or both Ile_2_ and Ser_3_ and Ile_6_ and Ser_7_. We used this approach to synthesize prodrugs of the teixobactin analogues, Lys_10_–teixobactin, Arg_10_–teixobactin, and Leu_10_–teixobactin. The Arg_10_ analogue replaces the cyclic guanidinium group of *allo*-enduracididine (*allo*-End) with an acyclic guanidinium group and exhibits good antibiotic activity, albeit not as good as the native antibiotic.^11,18–20^ The Lys_10_ analogue also contains a positively charged residue and exhibits good antibiotic activity.^11,20^ The Leu_10_ analogue is especially interesting, because it contains an uncharged residue, yet exhibits good antibiotic activity.^20–22^ Although the lack of commercial sources of *allo*-enduracididine makes it more difficult to access the corresponding prodrugs of teixobactin, we anticipate that the synthetic route described here should also allow the synthesis of teixobactin prodrugs.

**Fig. 4:**
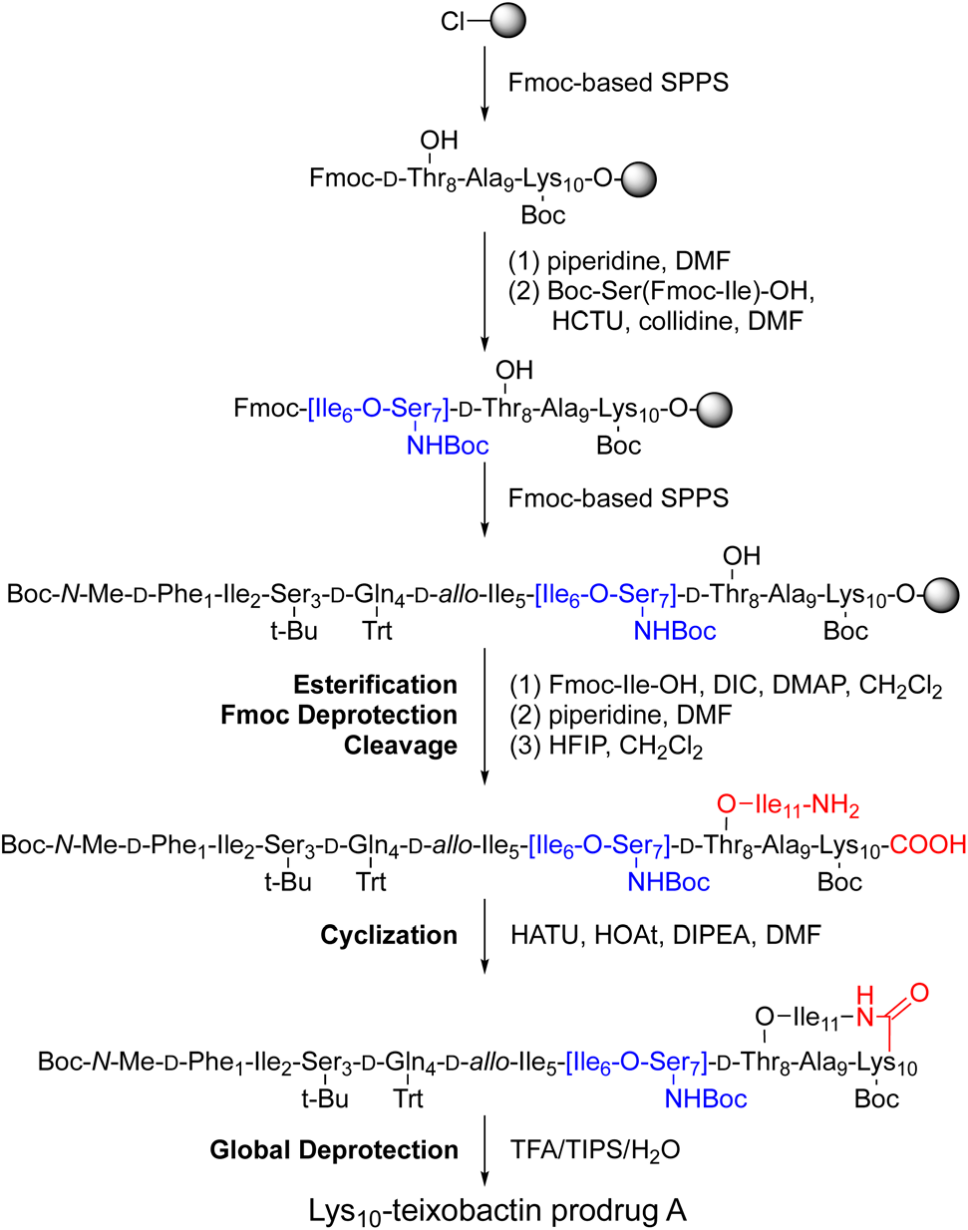
Synthesis of Lys_10_–teixobactin prodrug A.

The synthesis of these *O*-acyl isopeptide prodrugs begins by attaching Fmoc-Lys(Boc)-OH, Fmoc-Arg(Pbf)-OH, or Fmoc-Leu-OH to 2-chlorotrityl chloride resin. Residues 9 through 1 are then introduced by standard Fmoc-based SPPS using HCTU as the coupling reagent.^11^ Boc-Ser(Fmoc-Ile)-OH is coupled in place of the desired Ile and Ser residues to provide an ester linkage in place of an amide linkage. Ile_11_ is then introduced through an esterification using DIC and DMAP.^11^ Fmoc deprotection followed by cleavage of the peptide from resin with 20% hexafluoroisopropanol (HFIP) in CH_2_Cl_2_ affords the selectively deprotected uncyclized peptide. Solution-phase macrolactamization with HATU and HOAt, followed by global deprotection with trifluoroacetic acid (TFA) and RP-HPLC purification affords the desired *O*-acyl isopeptide prodrug analogue of teixobactin as the trifluoroacetate salt. Fig. 4 illustrates this route with the synthesis of Lys_10_–teixobactin prodrug A. Synthesis on a 0.1 mmol scale typically yields *ca.* 10 mg of the *O*-acyl isopeptide prodrug.

The trifluoroacetate salts of the *O*-acyl isopeptide teixobactin analogues are more soluble and easier to handle than the corresponding teixobactin analogues. When synthesizing active teixobactin analogues, we routinely have to dissolve the compounds in 30–40% aqueous acetonitrile for preparative HPLC injection, while only 10–20% acetonitrile is required for the *O*-acyl isopeptide teixobactin prodrug analogues. We routinely handle the *O*-acyl isopeptide teixobactin prodrug analogues as 10 mg mL^−1^ aqueous solutions, while the corresponding teixobactin analogues are not soluble at this concentration and form gels (Fig. S1†). The introduction of the *O*-acyl isopeptide linkage thus improves the solubility and handling of the prodrugs by reducing their propensity to aggregate in aqueous solutions.

### Conversion of the prodrugs to teixobactin analogues

**Fig. 5:**
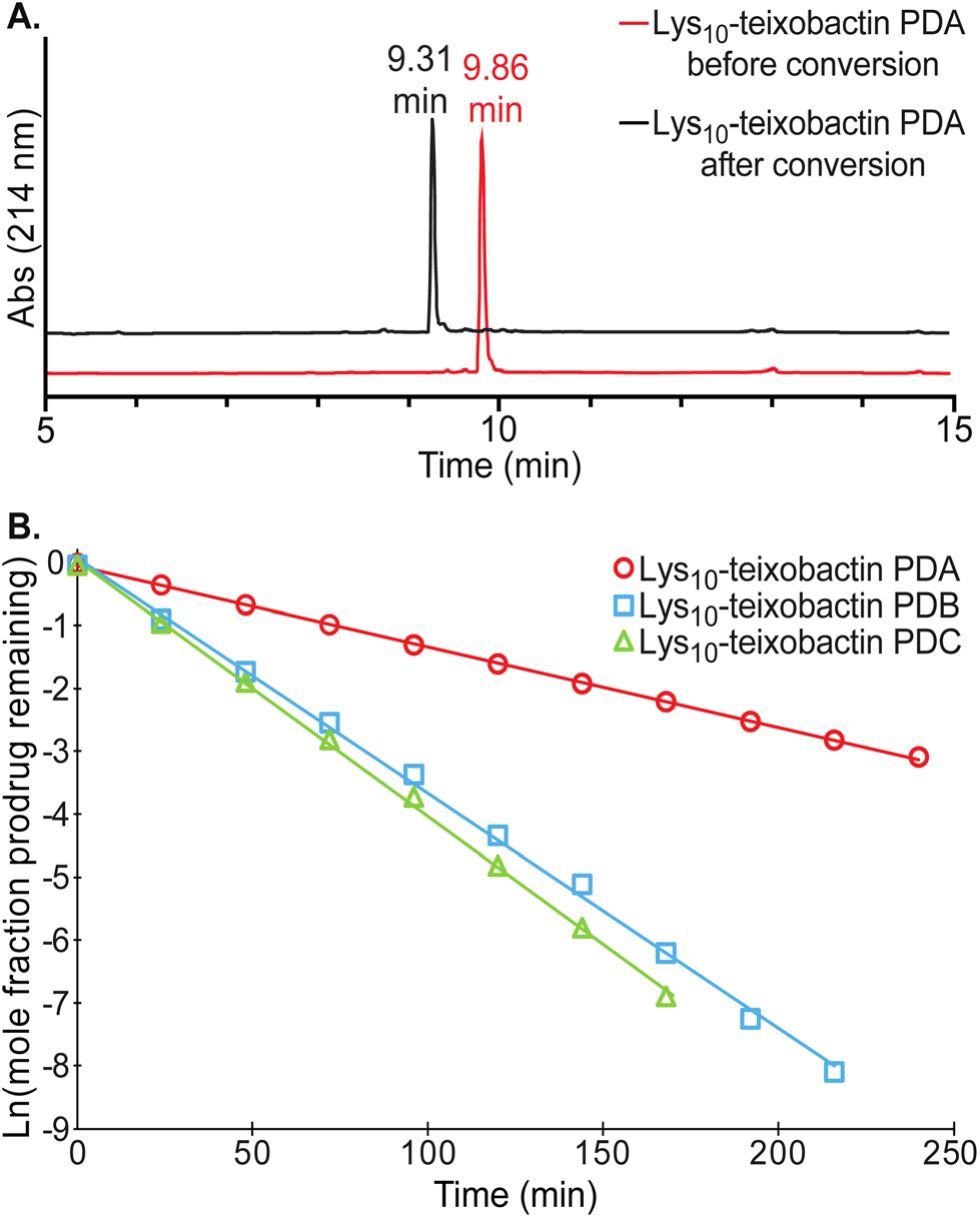
(A) Analytical RP-HPLC trace showing clean conversion of Lys_10_–teixobactin prodrug A (red) to Lys_10_–teixobactin (black). HPLC was run on a C18 column with a gradient of 5–100% acetonitrile over 20 min. (B) Conversion kinetics of Lys_10_–teixobactin prodrugs A, B, and C, illustrating the disappearance of each prodrug over time. All reactions were run at 23 ± 2 °C in 50 mM phosphate buffer at pH 7.4 and monitored by HPLC analysis on a C18 column with a gradient of 5–67% acetonitrile over 15 min.

Each of the teixobactin prodrugs undergoes clean conversion to the corresponding teixobactin analogue at physiological pH. When each of the A and B series of prodrugs was incubated in 50 mM phosphate buffer at pH 7.4 and the conversion reaction was monitored by HPLC, a new peak appeared in the HPLC trace corresponding to the teixobactin analogue. Fig. 5A illustrates the clean conversion of Lys_10_–teixobactin prodrug A to Lys_10_–teixobactin. No intermediates were observed, and the conversion was complete within 12–24 h at 23 ± 2 °C (Fig. S2 and S3†). When each of the C series of prodrugs was incubated in 50 mM phosphate buffer under similar conditions, two intermediates were observed, and conversion to the corresponding teixobactin analogue was complete within 12–24 h (Fig. S4†). The intermediates correspond to the A and B series prodrugs, which form upon isomerization of the Ile_2_–Ser_3_ and Ile_6_–Ser_7_ isopeptide linkages.

The conversion of the A and B series of prodrugs to the corresponding teixobactin analogues shows clean first-order kinetics for the disappearance of the prodrugs and the appearance of the corresponding teixobactin analogues. The conversion of the C series of prodrugs also shows clean first-order kinetics for the disappearance of the prodrugs (Fig. S5 and S6†). The A- and B-series prodrugs form as intermediates during conversion of the C series and then undergo subsequent conversion to the corresponding teixobactin analogues (Fig. S7–S9†). Fig. 5B illustrates the conversion kinetics for the disappearance of Lys_10_–teixobactin prodrugs A, B, and C. The A-series prodrugs exhibited half-lives of 54–115 minutes in phosphate buffer at 23 ± 2 °C, while the B- and C-series prodrugs exhibited half-lives of 13–36 minutes (Table 1). The shorter half-lives of the B- and C-series prodrugs may reflect effects of the *N*-terminal methylammonium group on the p*K*_a_ of the proximal Ser_3_ ammonium group in the B- and C-series *O*-acyl isopeptides.

**Table 1:** Half-lives for the teixobactin *O*-acyl isopeptide prodrug analogues^a^

	Half-life (min)
Lys_10_–teixobactin prodrug A	54^b^
Lys_10_–teixobactin prodrug B	18^b^
Lys_10_–teixobactin prodrug C	15^c^
Arg_10_–teixobactin prodrug A	65^b^
Arg_10_–teixobactin prodrug B	25^b^
Arg_10_–teixobactin prodrug C	13^c^
Leu_10_–teixobactin prodrug A	115^b^
Leu_10_–teixobactin prodrug B	36^b^
Leu_10_–teixobactin prodrug C	19^c^

a All half-lives were determined at 23 ± 2 °C in 50 mM phosphate buffer at pH 7.4.

b Prodrugs A and B convert directly to the corresponding teixobactin analogues.

c Prodrugs C convert to prodrugs A and B before converting to the corresponding teixobactin analogues. The half-lives reported for prodrugs C are the half-lives for the disappearance of prodrugs C. Conversion of all prodrugs to the corresponding teixobactin analogues occurs cleanly and quantitatively.

Conversion of the prodrugs to the corresponding teixobactin analogues occurs more rapidly at 37 °C. When the Lys_10_–teixobactin series of prodrugs was incubated at 37 °C in 100 mM phosphate buffer at pH 7.4, conversion to Lys_10_–teixobactin occurred 3–5 times faster, with half-lives of 4–10 minutes (Fig. S10 and S11†).

### Antibiotic activity

The *O*-acyl isopeptide prodrugs exhibit comparable or slightly improved antibiotic activity compared to the corresponding teixobactin analogues (Table 2). We evaluated the antibiotic activity of the teixobactin *O*-acyl isopeptide prodrugs using minimum inhibitory concentration (MIC) assays with four Gram-positive bacteria and compared the MIC values to those of the parent teixobactin analogues. We used methicillin-susceptible and methicillin-resistant *S. aureus* as representative pathogens and *B. subtilis* and *S. epidermidis* as additional Gram-positive bacteria, and we used *E. coli* as a Gram-negative control. We compared the activities of the teixobactin analogues and prodrugs to those of teixobactin and vancomycin.

**Table 2:** MIC values of teixobactin *O*-acyl isopeptide prodrug analogues, teixobactin analogues, teixobactin, and vancomycin in μg mL^−1^ with 0% and 0.002% polysorbate 80

	*Bacillus subtilis* ATCC 6051	*Staphylococcus epidermidis* ATCC 14990	*Staphylococcus aureus* (MSSA) ATCC 29213	*Staphylococcus aureus* (MRSA) ATCC 700698	*Escherichia coli* ATCC 10798
Lys_10_–teixobactin	2^a^	2–4^a^	4^a^	4^a^	>32^a^
	0.25^b^	2^b^	2^b^	2^b^	>8^b^
Lys_10_–teixobactin prodrug A	1^a^	2^a^	2^a^	2^a^	>32^a^
	0.125^b^	1^b^	1–2^b^	1^b^	>8^b^
Lys_10_–teixobactin prodrug B	0.5–1^a^	2^a^	2^a^	2^a^	>32^a^
	0.25^b^	1^b^	1–2^b^	1^b^	>8^b^
Lys_10_–teixobactin prodrug C	0.5–1^a^	2^a^	2^a^	2^a^	>32^a^
	0.125^b^	1^b^	1^b^	1^b^	>8^b^
Arg_10_–teixobactin	1^a^	1^a^	2^a^	2^a^	>32^a^
	0.5^b^	1^b^	1^b^	1^b^	>8^b^
Arg_10_–teixobactin prodrug A	1^a^	2^a^	2^a^	1^a^	>32^a^
	0.25^b^	1^b^	1^b^	1^b^	>8^b^
Arg_10_–teixobactin prodrug B	1^a^	2^a^	2^a^	2^a^	>32^a^
	0.25^b^	0.5–1^b^	2^b^	1^b^	>8^b^
Arg_10_–teixobactin prodrug C	1^a^	2^a^	2^a^	2^a^	>32^a^
	0.25^b^	0.5^b^	1^b^	1^b^	>8^b^
Leu_10_–teixobactin	2^a^	2^a^	2^a^	2^a^	>32^a^
	0.5–1^b^	2^b^	0.25–0.5^b^	0.5–1^b^	>8^b^
Leu_10_–teixobactin prodrug A	1^a^	1^a^	2^a^	1^a^	>32^a^
	0.125^b^	0.5^b^	0.5^b^	0.5^b^	>8^b^
Leu_10_–teixobactin prodrug B	0.5^a^	1^a^	1^a^	1^a^	>32^a^
	0.0625^b^	0.25–0.5^b^	0.25–0.5^b^	0.5^b^	>8^b^
Leu_10_–teixobactin prodrug C	1^a^	2^a^	2^a^	1^a^	>32^a^
	0.125^b^	0.5^b^	0.5^b^	0.5^b^	>8^b^
Teixobactin	0.0312^a^	1^a^	1^a^	0.5^a^	32^a^
	0.0078^b^	0.0078^b^	0.5^b^	0.25^b^	>8^b^
Vancomycin	0.125–0.25^a^	1^a^	0.5^a^	2^a^	>32^a^
	0.5^b^	2^b^	1^b^	2^b^	>8^b^

a Culture media containing 0% polysorbate 80.

b Culture media containing 0.002% polysorbate 80.

The Lys_10_–teixobactin prodrugs showed slightly improved activity compared to Lys_10_–teixobactin. Thus, the Lys_10_–teixobactin prodrugs exhibited MICs of 0.5–2 μg mL^−1^, while Lys_10_–teixobactin exhibited MICs of 2–4 μg mL^−1^. The Arg_10_–teixobactin prodrugs showed comparable antibiotic activity to Arg_10_–teixobactin, with MICs of 1–2 μg mL^−1^. The Leu_10_–teixobactin prodrugs showed equal or slightly improved antibiotic activity compared to Leu_10_–teixobactin, with MICs of 0.5–2 μg mL^−1^.

In the original report on teixobactin, the authors performed MIC assays in the presence of 0.002% polysorbate 80, with the rationale that the polysorbate 80 prevented teixobactin from sticking to plastic surfaces.^1,23,24^ Our laboratory has previously found that addition of polysorbate 80 can have dramatic effects upon the measured MIC value.^11^ When we performed MIC assays with the Lys_10_-, Arg_10_-, and Leu_10_–teixobactin analogues and the corresponding prodrugs A, B, and C in the presence of polysorbate 80, we also observed enhanced antibiotic activity. Lys_10_–teixobactin exhibited MICs of 0.25–2 μg mL^−1^, and the Lys_10_–teixobactin prodrugs exhibited MICs of 0.125–2 μg mL^−1^. Arg_10_–teixobactin and the Arg_10_–teixobactin prodrugs exhibited MICs of 0.25–2 μg mL^−1^. Leu_10_–teixobactin exhibited MICs of 0.25–1 μg mL^−1^, and the Leu_10_–teixobactin prodrugs exhibited MICs of 0.0625–0.5 μg mL^−1^.

Although the prodrugs themselves are not expected to exhibit antibiotic activity, conversion under the 37 °C assay conditions should be rapid enough to prevent the bacteria from propagating. The greater activity observed for some of the prodrugs may reflect higher effective drug concentrations resulting from complete dispersion of the prodrugs within the media. In the presence of 0.002% polysorbate 80, the Leu_10_–teixobactin prodrugs (MIC 0.0625–0.5 μg mL^−1^) are somewhat more active than vancomycin (MIC 0.125–2 μg mL^−1^), although somewhat less active than teixobactin itself (MIC 0.0078–1 μg mL^−1^).

### Gel formation

**Fig. 6:**
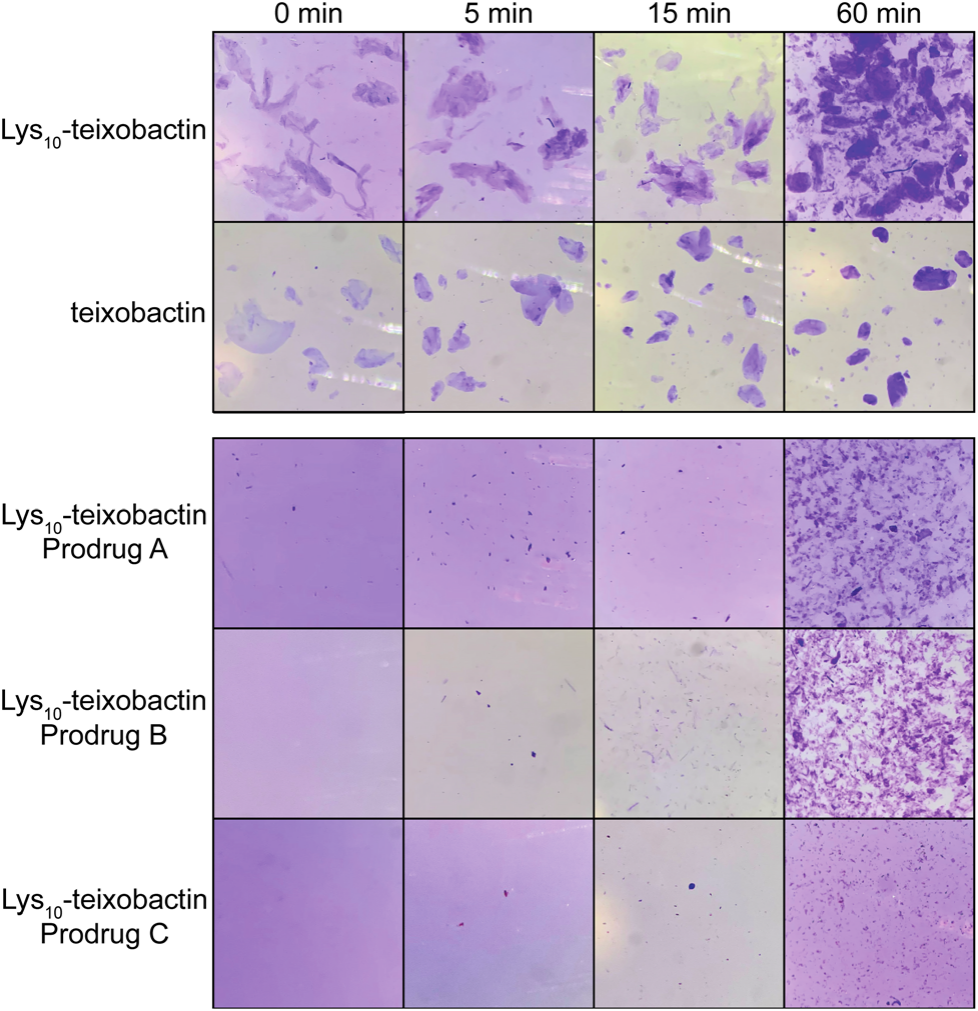
Gel formation of Lys_10_–teixobactin and teixobactin and delayed gel formation of Lys_10_–teixobactin prodrugs A, B, and C.

The *O*-acyl isopeptide prodrug analogues of teixobactin exhibit delayed gel formation at physiological pH. We compared gel formation of the teixobactin analogues to that of the *O*-acyl isopeptide prodrugs in a qualitative gelation assay. In this experiment, a 10 mg mL^−1^ stock solution of the peptide trifluoroacetate salt in DMSO is added to 1× PBS buffer at pH 7.4 and gel formation is observed over time.^9,12^ Crystal violet is added to the PBS buffer to facilitate the visualization of the gels by providing contrast. When Lys_10_–teixobactin is added to PBS, large gelatinous aggregates form immediately (Fig. 6). Similar behavior is also observed for teixobactin (as the hydrochloride salt). In contrast, when the Lys_10_–teixobactin prodrugs are added, no immediate gel formation occurs. After 5 min, a few small gelatinous aggregates become visible, with Lys_10_–teixobactin prodrug C showing the least amount of gel formation. After 15 min, the number of aggregates increases but the size of the aggregates does not. By 60 min, gel formation increases significantly, especially for Lys_10_–teixobactin prodrugs A and B, which also begin to form gelatinous aggregates of larger size. The Arg_10_–teixobactin prodrugs and Leu_10_–teixobactin prodrugs show similar behavior, not immediately forming gels when added to PBS, and then forming aggregates over 60 minutes (Fig. S12 and S13†). In contrast, Arg_10_–teixobactin and Leu_10_–teixobactin form gels immediately upon addition to PBS.

The gelation assays of the *O*-acyl isopeptide prodrugs demonstrate that these compounds do not gelate immediately upon exposure to buffer, unlike teixobactin and active teixobactin analogues. Thus, the prodrugs remain in solution and can be thoroughly dispersed in PBS. As the prodrugs gradually convert, they form aggregates that are smaller and more dispersed than those formed by teixobactin and the teixobactin analogues. The greater solubility of the prodrugs should impart better pharmacological properties than the parent analogues and may thus make them superior drug candidates.

### Hemolytic and cytotoxicity assays

We evaluated the hemolytic activity of the *O*-acyl isopeptide prodrugs and the corresponding teixobactin analogues with human red blood cells (Fig. S14–S21†). We used Triton X-100 and melittin as positive controls and vancomycin and water (vehicle) as negative controls in the hemolysis assays.^9,25,26^ The teixobactin analogues and corresponding *O*-acyl isopeptide prodrugs exhibited no hemolytic activity at concentrations up to 100 μg mL^−1^ in the absence of polysorbate 80. In the presence of 0.002% polysorbate 80, the Arg_10_–teixobactin analogue and corresponding *O*-acyl isopeptide prodrugs exhibited modest hemolytic activity, with 7–10% hemolytic activity occurring at 100 μg mL^−1^. In contrast, 0.002% polysorbate 80 had no effect on the hemolytic activity of Lys_10_–teixobactin and Leu_10_–teixobactin and little effect on the corresponding *O*-acyl isopeptide prodrug analogues. When we performed hemolysis assays with teixobactin, we observed no hemolysis up to 100 μg mL^−1^ without polysorbate 80 and modest hemolysis (4%) at 100 μg mL^−1^ with 0.002% polysorbate 80. We observed no hemolysis with vancomycin at concentrations up to 100 μg mL^−1^, and we observed 26–30% hemolysis with 1.25 μg mL^−1^ melittin with and without 0.002% polysorbate 80. Collectively, these studies suggest the *O*-acyl isopeptide prodrug analogues should be suitable for intravenous administration at concentrations well above the MIC values.

To further assess the potential of the teixobactin *O*-acyl isopeptide prodrug analogues as potential drugs we performed cytotoxicity assays (Fig. S22–S27†). We evaluated the cytotoxicity of the teixobactin *O*-acyl isopeptide prodrugs and the corresponding teixobactin analogues on HeLa cells using a Promega Cytotox-Glo assay. In these experiments, the Lys_10_- and Arg_10_–teixobactin *O*-acyl isopeptide prodrugs exhibited no cytotoxicity at concentrations up to 50 μM (72–85 μg mL^−1^). The Leu_10_–teixobactin *O*-acyl isopeptide prodrugs exhibited no cytotoxicity at concentrations up to 25 μM (33–39 μg mL^−1^) and slight cytotoxicity at 50 μM (66–77 μg mL^−1^). These studies further suggest that the *O*-acyl isopeptide prodrug analogues should be suitable for intravenous administration at concentrations well above the MIC values.

### *In vivo* efficacy

**Fig. 7:**
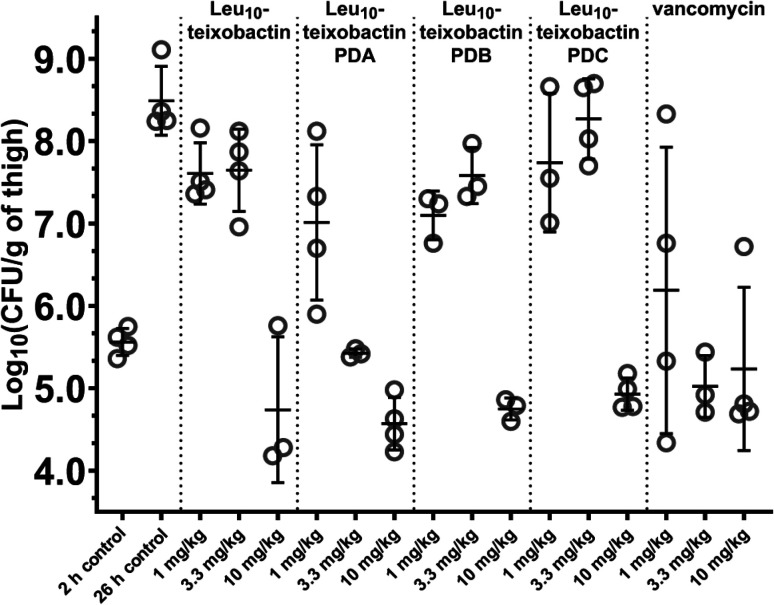
Neutropenic mouse thigh infection efficacy model against MRSA (ATCC BAA-1717) of the Leu_10_–teixobactin prodrugs and Leu_10_–teixobactin, with vancomycin as a positive control.

We evaluated the *in vivo* efficacy of the Leu_10_–teixobactin *O*-acyl isopeptide prodrugs and Leu_10_–teixobactin in a mouse thigh infection model against MRSA (Fig. 7 and S28†). In this experiment, mice were rendered neutropenic and then infected with MRSA (ATCC BAA-1717). After 2 h, the mice were treated with the Leu_10_–teixobactin prodrugs, Leu_10_–teixobactin, or vancomycin with intravenous BID dosing at 1, 3.3, and 10 mg kg^−1^. After an additional 24 h, the mice were euthanized and the bacterial loads of the treated mice were compared to those of the untreated mice at the 2 and 26 hour time points. At 10 mg kg^−1^ dosing, Leu_10_–teixobactin and each of the Leu_10_–teixobactin prodrugs lowered the bacterial loads by 3–4 orders of magnitude over the untreated mice at the 26 hour time point and also lowered the bacterial load below the untreated mice at the 2 hour time point. Leu_10_–teixobactin prodrug A also substantially lowered the bacterial load at 3.3 mg kg^−1^ dosing and thus appeared to exhibit the greatest efficacy, demonstrating activity comparable to vancomycin.

## Conclusions

The propensity of teixobactin to aggregate and form gels in aqueous conditions may lead to limitations in dosing or formulation, or even the failure of teixobactin to advance to and succeed in clinical trials. Teixobactin prodrugs and prodrug analogues containing *O*-acyl isopeptide linkages may circumvent this problem. The compounds have a reduced propensity to aggregate and do not form gels immediately upon exposure to physiological pH. The prodrug analogues convert quantitatively and cleanly to the corresponding teixobactin analogues and exhibit conversion half-lives of 13–115 minutes at room temperature. The prodrug analogues exhibit comparable or slightly improved antibiotic activity against Gram-positive bacteria compared to the corresponding teixobactin analogues. The prodrug analogues have MIC values of 0.5–2 μg mL^−1^ without addition of polysorbate 80, and even lower MIC values in the presence of 0.002% polysorbate 80. Furthermore, the prodrug analogues exhibit low hemolytic activity with or without polysorbate 80 and do not exhibit any significant cytotoxicity. The Leu_10_–teixobactin series exhibited efficacy upon intravenous dosing in an *in vivo* infection model against MRSA.

We anticipate that intravenous administration of *O*-acyl isopeptide prodrugs of teixobactin and teixobactin analogues in human patients will allow thorough mixing and dilution in the bloodstream before conversion to the corresponding drugs. The mixing and dilution should mitigate the formation of gels and thus facilitate intravenous administration, providing a more straightforward path for teixobactin derivatives to progress to and succeed in clinical trials.

Teixobactin *O*-acyl isopeptide prodrug analogues containing leucine at position 10 exhibit comparable antibiotic activity to vancomycin. We expect that teixobactin *O*-acyl isopeptide prodrugs containing *allo*-enduracidine at position 10 will exhibit even better antibiotic activity. Because teixobactin *O*-acyl isopeptide prodrug analogues exhibit clean conversion to the corresponding teixobactin analogues with reduced propensity to form gels, we anticipate that teixobactin prodrugs will be superior to teixobactin as drug candidates. Even the Lys_10_-, Arg_10_-, and Leu_10_–teixobactin prodrug analogues reported here exhibit good antibiotic activity, with Leu_10_–teixobactin prodrugs A, B, and C being especially noteworthy. We propose the term “isobactins” for teixobactin prodrugs and teixobactin prodrug analogues, to reflect that they isomerize to teixobactin and teixobactin analogues. We believe that the isobactins warrant further study, because the isobactins overcome a potential limitation in the intravenous dosing of teixobactin.

## Author contributions

J. S. N. and C. R. J. collaborated on the conception of the project, with J. S. N. proposing the A-series prodrugs and C. R. J. proposing the B- and C-series prodrugs. J. S. N. supervised the project. C. R. J. and G. H. L. synthesized the peptides, and C. R. J. characterized the peptides. C. R. J. performed the conversion, MIC, gelation, and hemolytic experiments. G. G. and C. R. J. performed mammalian cell work and the cytotoxicity experiments. J. S. N. and C. R. J. wrote and edited the manuscript.

## Conflicts of interest

J. S. N. and C. R. J. have submitted a patent application on prodrugs of teixobactin and teixobactin analogues through the Regents of the University of California. The authors have collaborated with and received subcontracts from NovoBiotic Pharmaceuticals, LLC, albeit not for this project.

## Supplementary Material

SC-013-D2SC02670H-s001
