# Supplementary material for: Isobactins: O-acyl isopeptide prodrugs of teixobactin and teixobactin derivatives
Source: Chem Sci. 2022 Oct 24;13(44):13110–6. doi: 10.1039/d2sc02670h (PMC9667932; doi:10.1039/d2sc02670h)
Supplement: SC-013-D2SC02670H-s001 [file SC-013-D2SC02670H-s001.pdf]

Electronic Supplementary Material (ESI) for Chemical Science.
This journal is © The Royal Society of Chemistry 2022

Supporting Information for:

Isobactins: *O*-Acyl Isopeptide Prodrugs of Teixobactin and Teixobactin Derivatives

Chelsea R. Jones,[a] Gretchen Guaglianone,[a] Grant H. Lai,[a] and James S. Nowick*,[a,b]
E-mail: jsnowick@uci.edu

[a]Department of Chemistry, University of California, Irvine,
Irvine, California 92697, United States

[b]Department of Pharmaceutical Sciences, University of California, Irvine,
Irvine, California 92697, United States

Table of Contents

Supplementary figures

Figure S1. Solubility of Lys_{10}-teixobactin prodrugs A, B, and C and Lys_{10}-teixobactin in H_2O as a 10 mg/mL solution S5

Figure S2. Analytical trace of the complete conversion of Arg_{10}-teixobactin prodrug A after 24 h S5

Figure S3. Analytical trace of the complete conversion of Arg_{10}-teixobactin prodrug B after 24 h S6

Figure S4. Analytical trace of the complete conversion of Arg_{10}-teixobactin prodrug C after 24 h S6

Figure S5. Conversion kinetics of all Arg_{10}-teixobactin prodrugs at rt S7

Figure S6. Conversion kinetics of all Leu_{10}-teixobactin prodrugs at rt S7

Figure S7. Conversion of Lys_{10}-teixobactin prodrug C at rt S8

Figure S8. Conversion of Arg_{10}-teixobactin prodrug C at rt S8

Figure S9. Conversion of Leu_{10}-teixobactin prodrug C at rt S9

Figure S10. Conversion kinetics of all Lys_{10}-teixobactin prodrugs at 37 °C S10

Figure S11. Conversion of Lys_{10}-teixobactin prodrug C at 37 °C S10

Figure S12. Gel formation of Arg_{10}-teixobactin and teixobactin and delayed gel formation of Arg_{10}-teixobactin prodrugs A, B, and C S11

Figure S13. Gel formation of Leu_{10}-teixobactin and teixobactin and delayed gel formation of Leu_{10}-teixobactin prodrugs A, B, and C S12

Figure S14. Hemolytic assay of Lys_{10}-teixobactin and the Lys_{10}-teixobactin prodrugs without polysorbate 80 S13

Figure S15. Hemolytic assay of Lys_{10}-teixobactin and the Lys_{10}-teixobactin prodrugs with 0.002% polysorbate 80 S13

Figure S16. Hemolytic assay of Arg_{10}-teixobactin and the Arg_{10}-teixobactin prodrugs without polysorbate 80 S14

Figure S17. Hemolytic assay of Arg_{10}-teixobactin and the Arg_{10}-teixobactin prodrugs with 0.002% polysorbate 80 S14

Figure S18. Hemolytic assay of Leu_{10}-teixobactin and the Leu_{10}-teixobactin prodrugs without polysorbate 80 S15

Figure S19. Hemolytic assay of Leu_{10}-teixobactin and the Leu_{10}-teixobactin prodrugs with 0.002% polysorbate 80 S15

Figure S20. Hemolytic assay of teixobactin and vancomycin without polysorbate 80 S16

Figure S21. Hemolytic assay of teixobactin and vancomycin with 0.002% polysorbate 80 S16

Figure S22. Cytotoxicity assay of Lys_{10}-teixobactin prodrug A and prodrug B with HeLa cells S17

Figure S23. Cytotoxicity assay of Lys_{10}-teixobactin prodrug C and Lys_{10}-teixobactin with HeLa cells S17

Figure S24. Cytotoxicity assay of Arg_{10}-teixobactin prodrug A and prodrug B with HeLa cells S18

Figure S25. Cytotoxicity assay of Arg_{10}-teixobactin prodrug C and Arg_{10}-teixobactin with HeLa cells S18

Figure S26. Cytotoxicity assay of Leu_{10}-teixobactin prodrug A and prodrug B with HeLa cells S19

Figure S27. Cytotoxicity assay of Leu_{10}-teixobactin prodrug C and Leu_{10}-teixobactin with HeLa cells S19

Figure S28. Complete data graph of the neutropenic mouse thigh efficacy study S20

Materials and Methods

Materials S21

Methods for synthesis, purification, and analysis of peptides S21

Synthesis of teixobactin *O*-acyl isopeptide prodrug analogues and their corresponding teixobactin analogues S22

Representative synthesis of Lys_{10}-teixobactin prodrug A S22

Table S1. Yields of purified teixobactin *O*-acyl isopeptide prodrug analogues and their corresponding teixobactin analogues S25

Conversion kinetic studies of teixobactin *O*-acyl isopeptide prodrug analogues at room temperature and 37 °C S25

MIC assays of teixobactin *O*-acyl isopeptide prodrug analogues and their corresponding teixobactin analogues S26

Figure S29. MIC assay plate layout S28

Gel formation studies of teixobactin *O*-acyl isopeptide prodrug analogues and their corresponding teixobactin analogues S28

Hemolytic assays of teixobactin *O*-acyl isopeptide prodrug analogues and their corresponding teixobactin analogues S28

Figure S30. Hemolytic assay plate layout S31

Cell culture and cytotoxicity assays of teixobactin *O*-acyl isopeptide prodrug analogues and their corresponding teixobactin analogues S31

Figure S31. Cytotoxicity assay plate layout S32

Conversion of the peptide TFA salts to HCl salts S33

Neutropenic mouse thigh infection efficacy study against MRSA S33

References for Supporting Information S35

Characterization of teixobactin analogues

Lys_{10}-teixobactin S36

Analytical HPLC Trace and MALDI-TOF Mass Spectrum

Lys_{10}-teixobactin prodrug A S38
Analytical HPLC Trace and MALDI-TOF Mass Spectrum

Lys_{10}-teixobactin prodrug B S40
Analytical HPLC Trace and MALDI-TOF Mass Spectrum

Lys_{10}-teixobactin prodrug C S42
Analytical HPLC Trace and MALDI-TOF Mass Spectrum

Arg_{10}-teixobactin S44
Analytical HPLC Trace and MALDI-TOF Mass Spectrum

Arg_{10}-teixobactin prodrug A S46
Analytical HPLC Trace and MALDI-TOF Mass Spectrum

Arg_{10}-teixobactin prodrug B S48
Analytical HPLC Trace and MALDI-TOF Mass Spectrum

Arg_{10}-teixobactin prodrug C S50
Analytical HPLC Trace and MALDI-TOF Mass Spectrum

Leu_{10}-teixobactin S52
Analytical HPLC Trace and MALDI-TOF Mass Spectrum

Leu_{10}-teixobactin prodrug A S54
Analytical HPLC Trace and MALDI-TOF Mass Spectrum

Leu_{10}-teixobactin prodrug B S56
Analytical HPLC Trace and MALDI-TOF Mass Spectrum

Leu_{10}-teixobactin prodrug C S58
Analytical HPLC Trace and MALDI-TOF Mass Spectrum

Supplementary figures

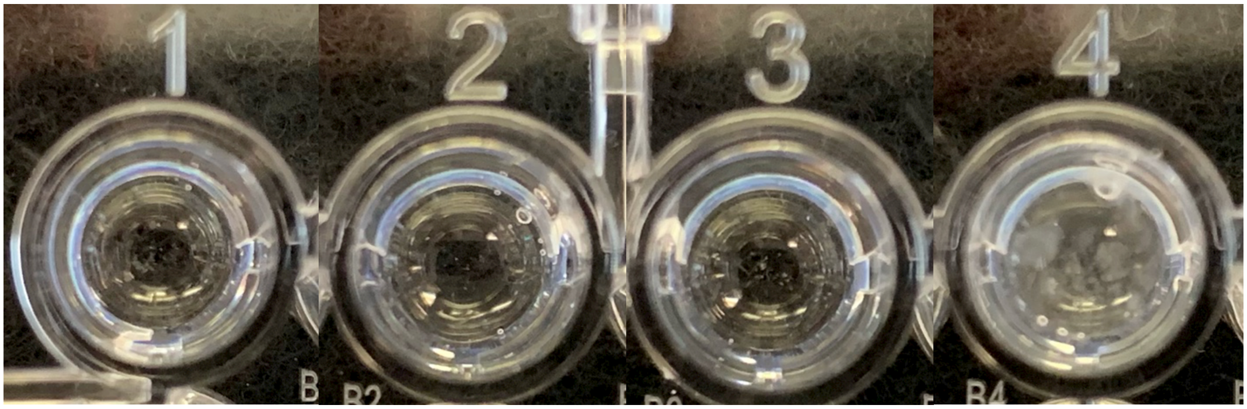

Figure S1. Solubility of Lys_{10}-teixobactin prodrug A, B, and C and Lys_{10}-teixobactin in water. Well 1: 1.0 mg of Lys_{10}-teixobactin prodrug A TFA salt in 100 µL of water. Well 2: 1.0 mg of Lys_{10}-teixobactin prodrug B TFA salt in 100 µL of water. Well 3: Lys_{10}-teixobactin prodrug C TFA salt in 100 µL of water. Well 4: 1.0 mg of Lys_{10}-teixobactin TFA salt in 100 µL of water. Lys_{10}-teixobactin prodrugs A, B, and C have solubility of at least 10 mg/mL in water and form clear solutions, while Lys_{10}-teixobactin is considerably less soluble and forms a gel.

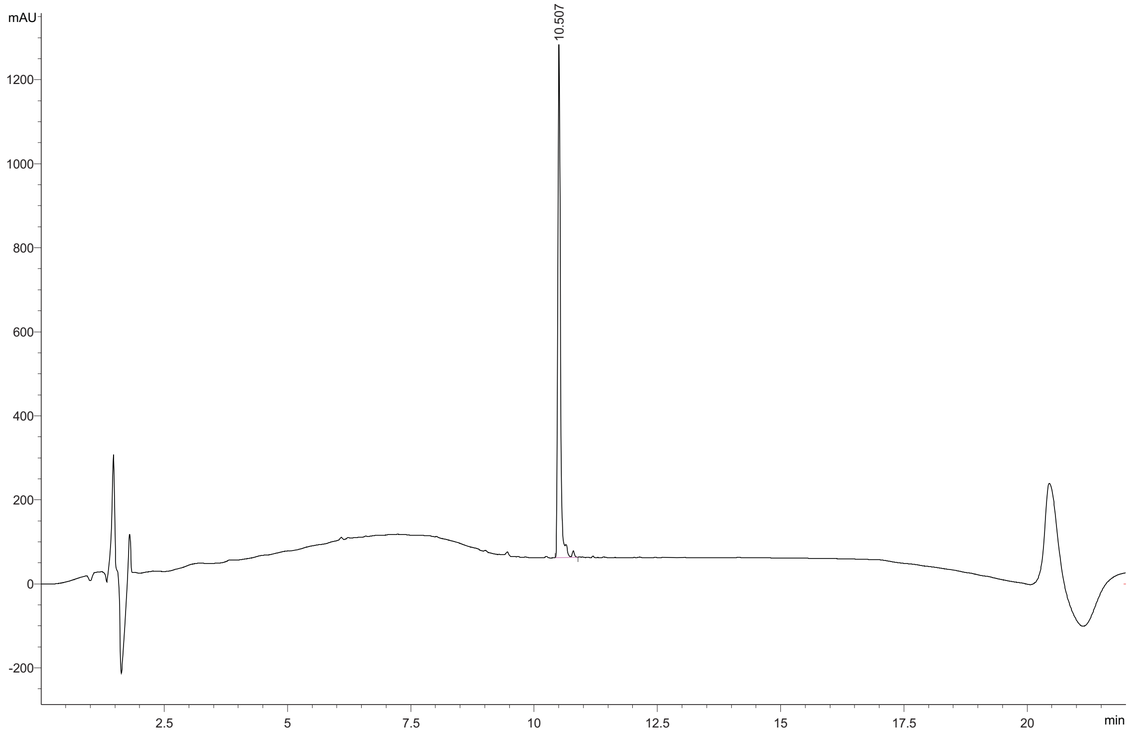

Figure S2. Analytical RP-HPLC trace showing the of the complete conversion of Arg_{10}-teixobactin prodrug A after 24 h in 50 mM sodium phosphate buffer at pH 7.4. HPLC was run on a C18 column with a gradient of 5–67% acetonitrile over 15 mins.

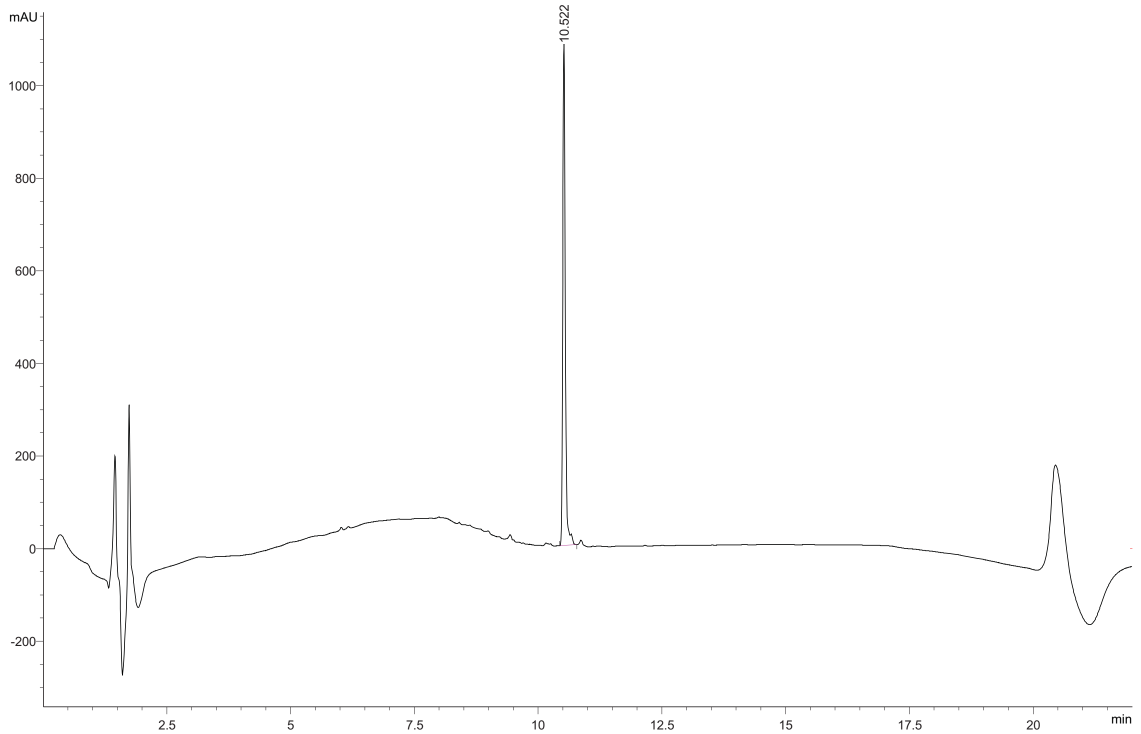

Figure S3. Analytical RP-HPLC trace showing the of the complete conversion of Arg_{10}-teixobactin prodrug B after 24 h in 50 mM sodium phosphate buffer at pH 7.4. HPLC was run on a C18 column with a gradient of 5–67% acetonitrile over 15 mins.

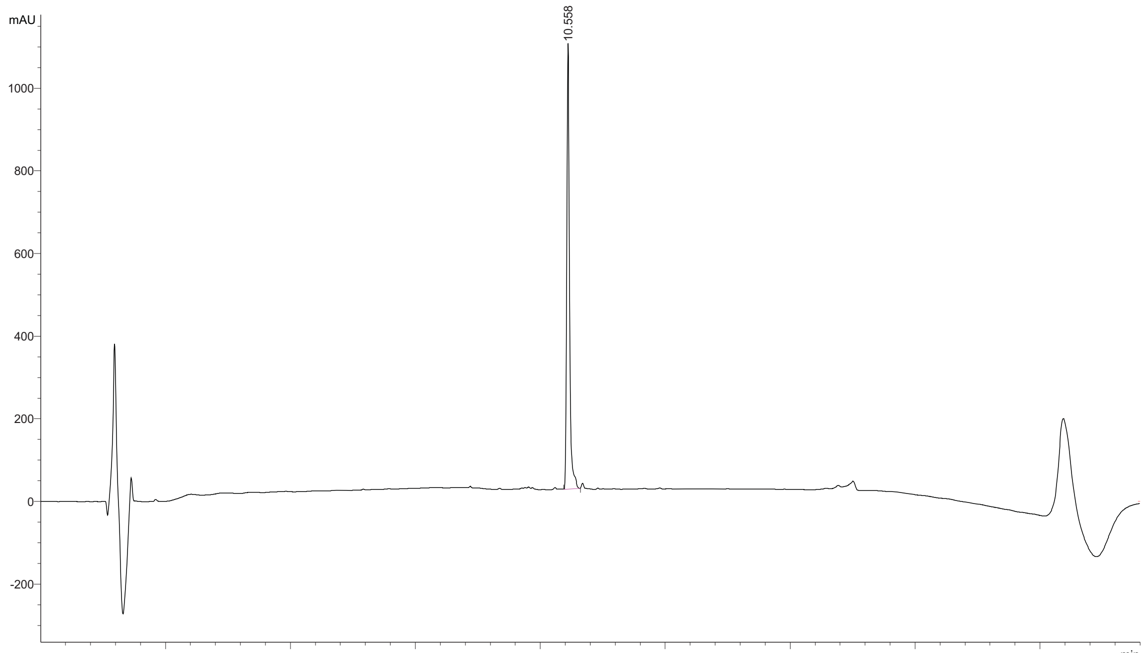

Figure S4. Analytical RP-HPLC trace showing the of the complete conversion of Arg_{10}-teixobactin prodrug C after 24 h in 100 mM sodium phosphate buffer at pH 7.4. HPLC was run on a C18 column with a gradient of 5–67% acetonitrile over 15 mins.

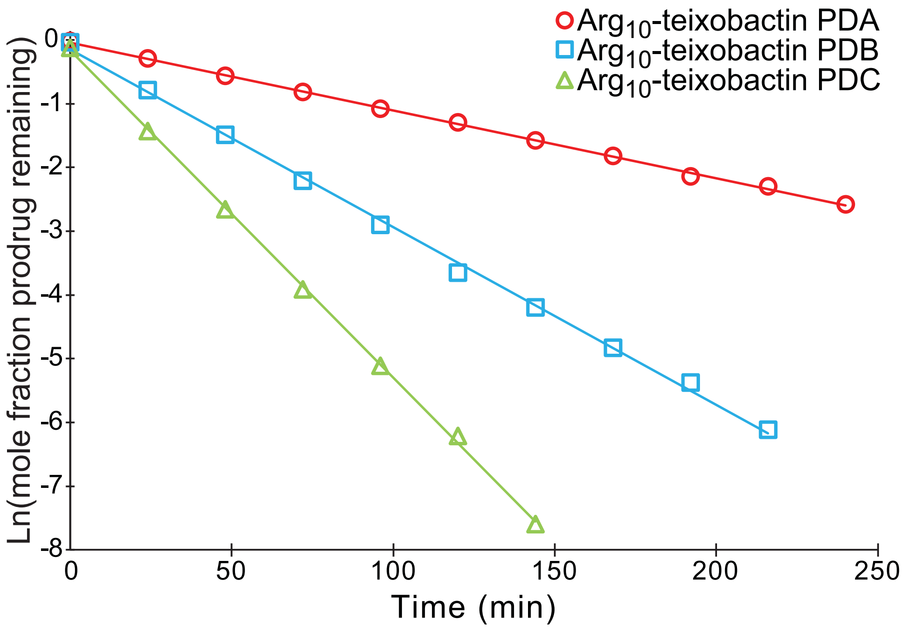

Figure S5. Conversion kinetics of Arg_{10}-teixobactin prodrugs A, B, and C, illustrating the disappearance of each prodrug over time. All reactions were run in 50 mM phosphate buffer at pH 7.4 and monitored by HPLC analysis on a C18 column with a gradient of 5–67% acetonitrile over 15 mins. Prodrugs A and B were run at 23 ± 1 °C; prodrug C was run at 25 ± 1 °C.

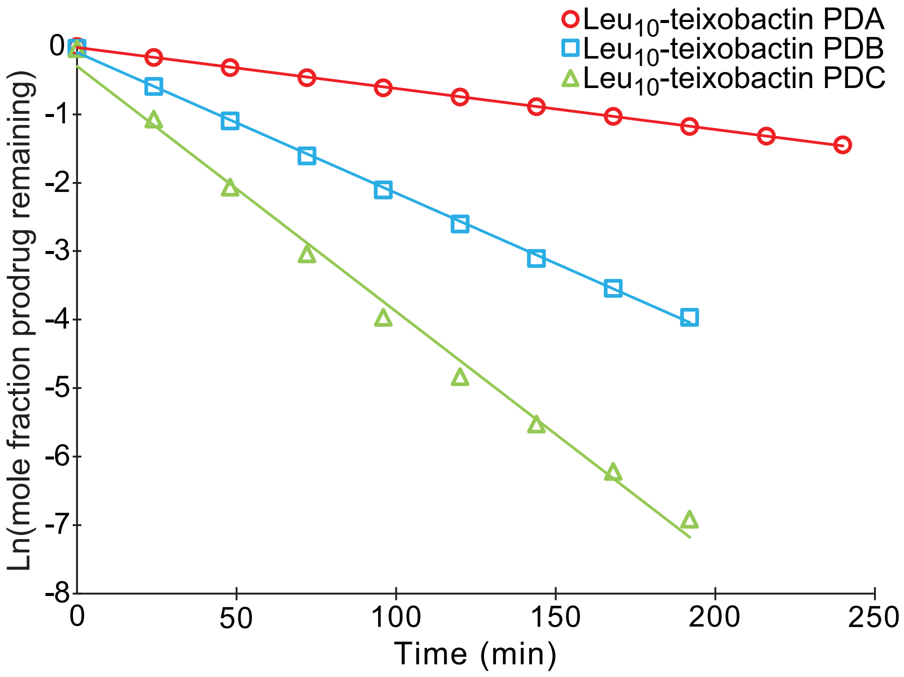

Figure S6. Conversion kinetics of Leu_{10}-teixobactin prodrugs A, B, and C, illustrating the disappearance of each prodrug over time. All reactions were run in 50 mM phosphate buffer at pH 7.4 and monitored by HPLC analysis on a C18 column with a gradient of 5–67% acetonitrile over 15 mins. Prodrugs A and B were run at 23 ± 1 °C; prodrug C was run at 25 ± 1 °C.

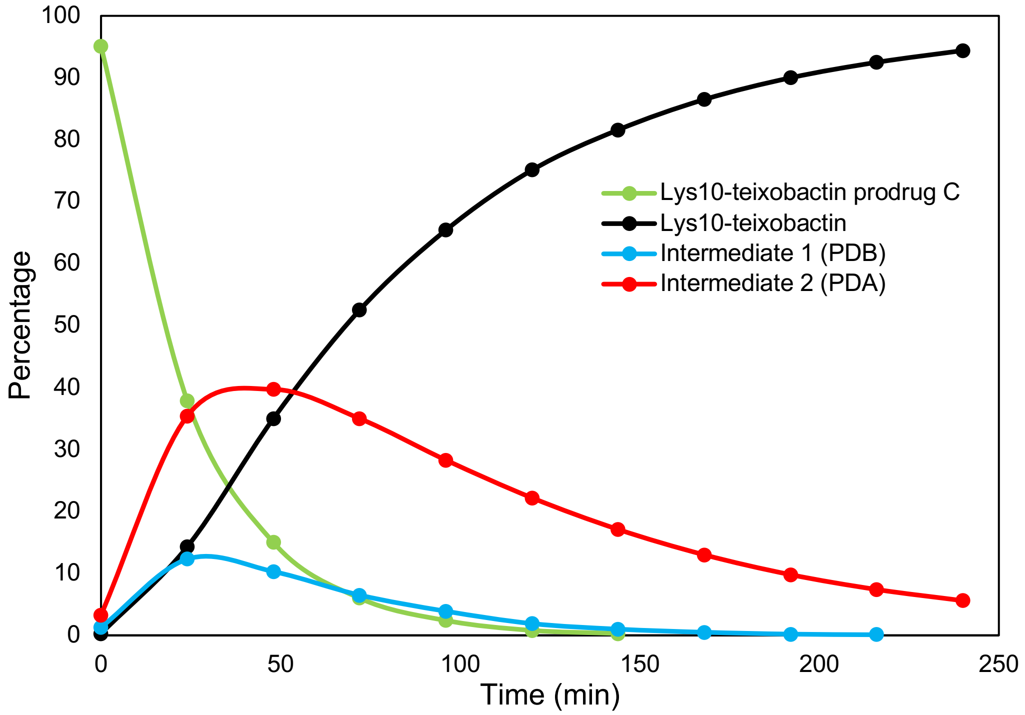

Figure S7. Conversion of Lys_{10}-teixobactin prodrug C (green) at 23 ± 2 °C, exhibiting the appearance of Lys_{10}-teixobactin (black) and intermediates corresponding to Lys_{10}-teixobactin prodrugs A (red) and B (blue).

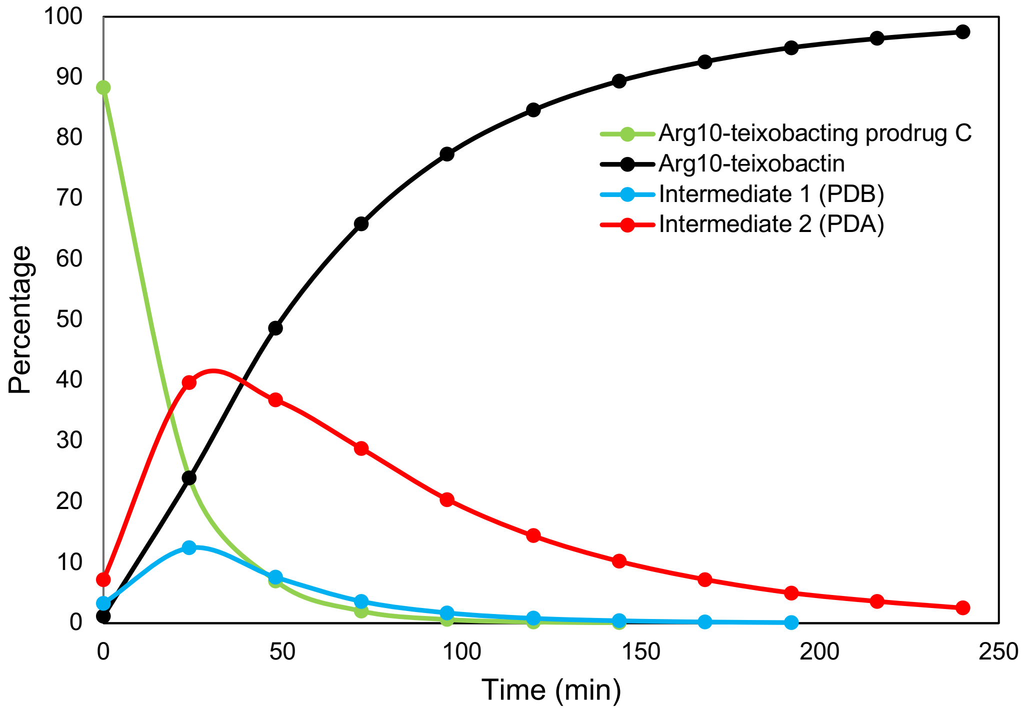

Figure S8. Conversion of Arg_{10}-teixobactin prodrug C (green) 25 ± 1 °C, exhibiting the appearance of Arg_{10}-teixobactin (black) and intermediates corresponding to Arg_{10}-teixobactin prodrugs A (red) and B (blue).

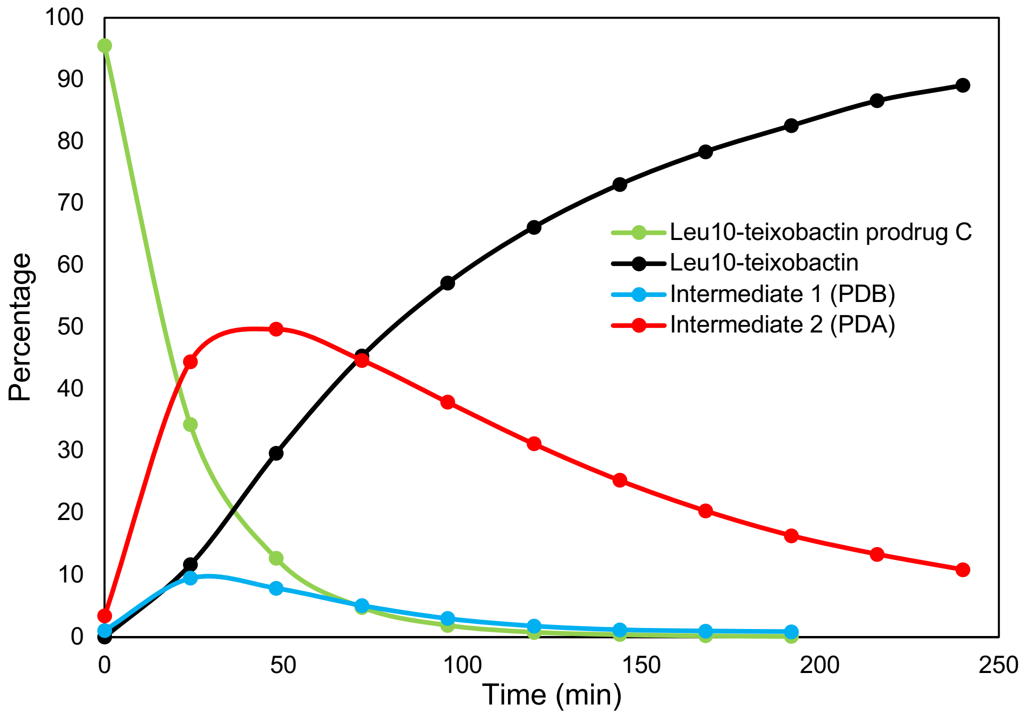

Figure S9. Conversion of Leu_{10}-teixobactin prodrug C (green) 25 ± 1 °C, exhibiting the appearance of Leu_{10}-teixobactin (black) and intermediates corresponding to Leu_{10}-teixobactin prodrugs A (red) and B (blue).

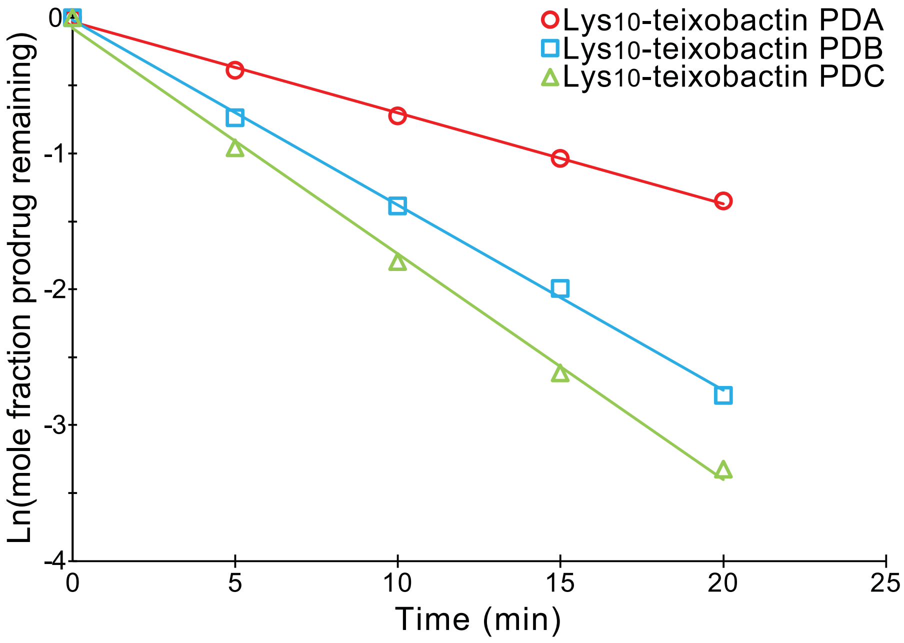

Figure S10. Conversion kinetics of Lys_{10}-teixobactin prodrugs A, B, and C, illustrating the disappearance of each prodrug over time at 37 °C. All reactions were run in 100 mM phosphate buffer at pH 7.4 and monitored by HPLC analysis on a C18 column with a gradient of 5–67% acetonitrile over 15 mins.

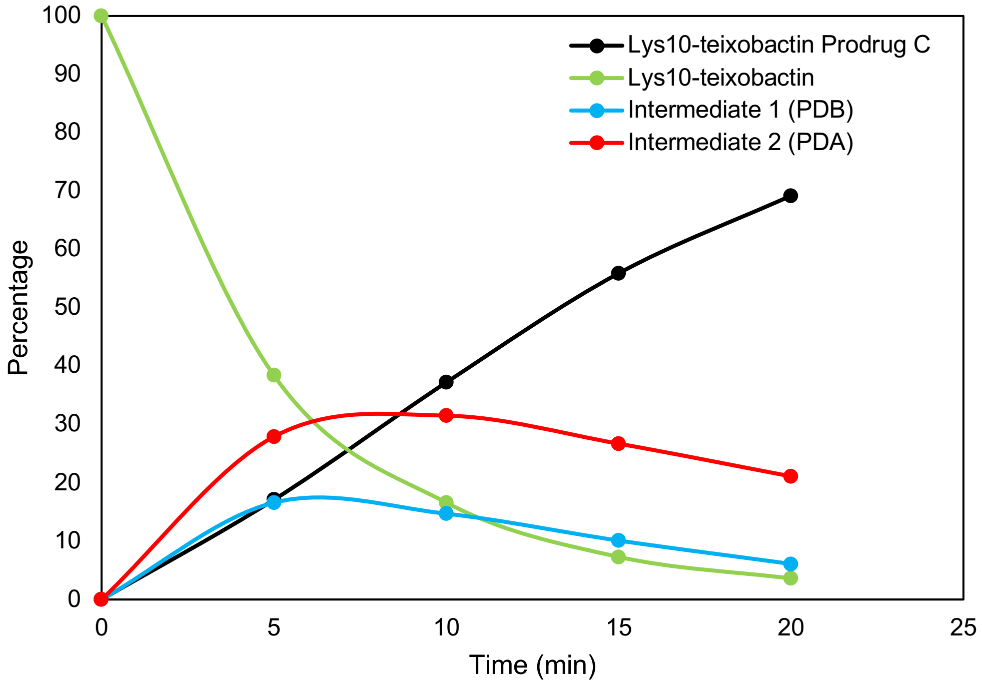

Figure S11. Conversion of Lys_{10}-teixobactin prodrug C (green) at 37 °C, exhibiting the appearance of Lys_{10}-teixobactin (black) and intermediates corresponding to Lys_{10}-teixobactin prodrugs A (red) and B (blue).

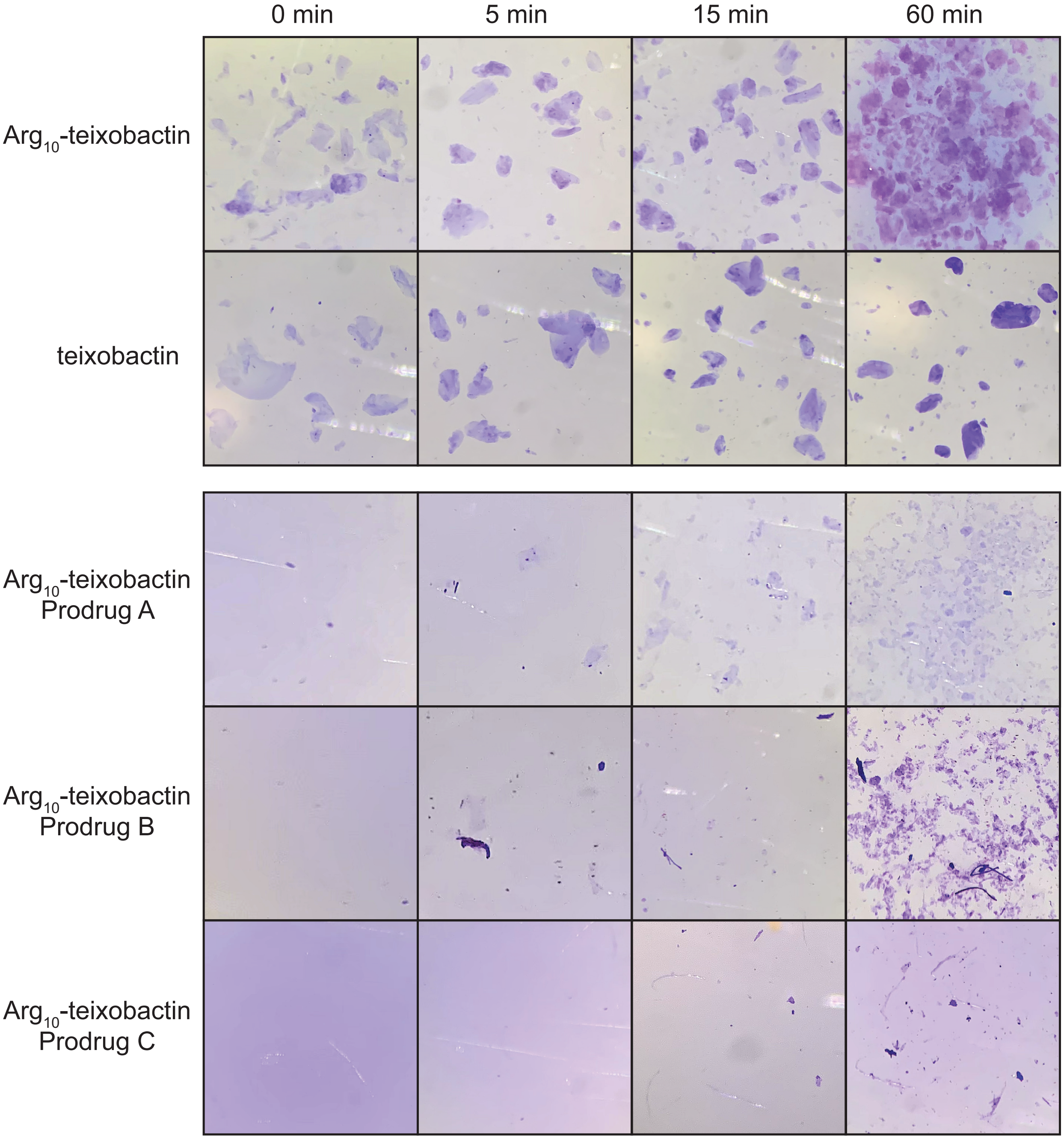

Figure S12. Gel formation of Arg_{10}-teixobactin and teixobactin and delayed gel formation of Arg_{10}-teixobactin prodrugs A, B, and C.

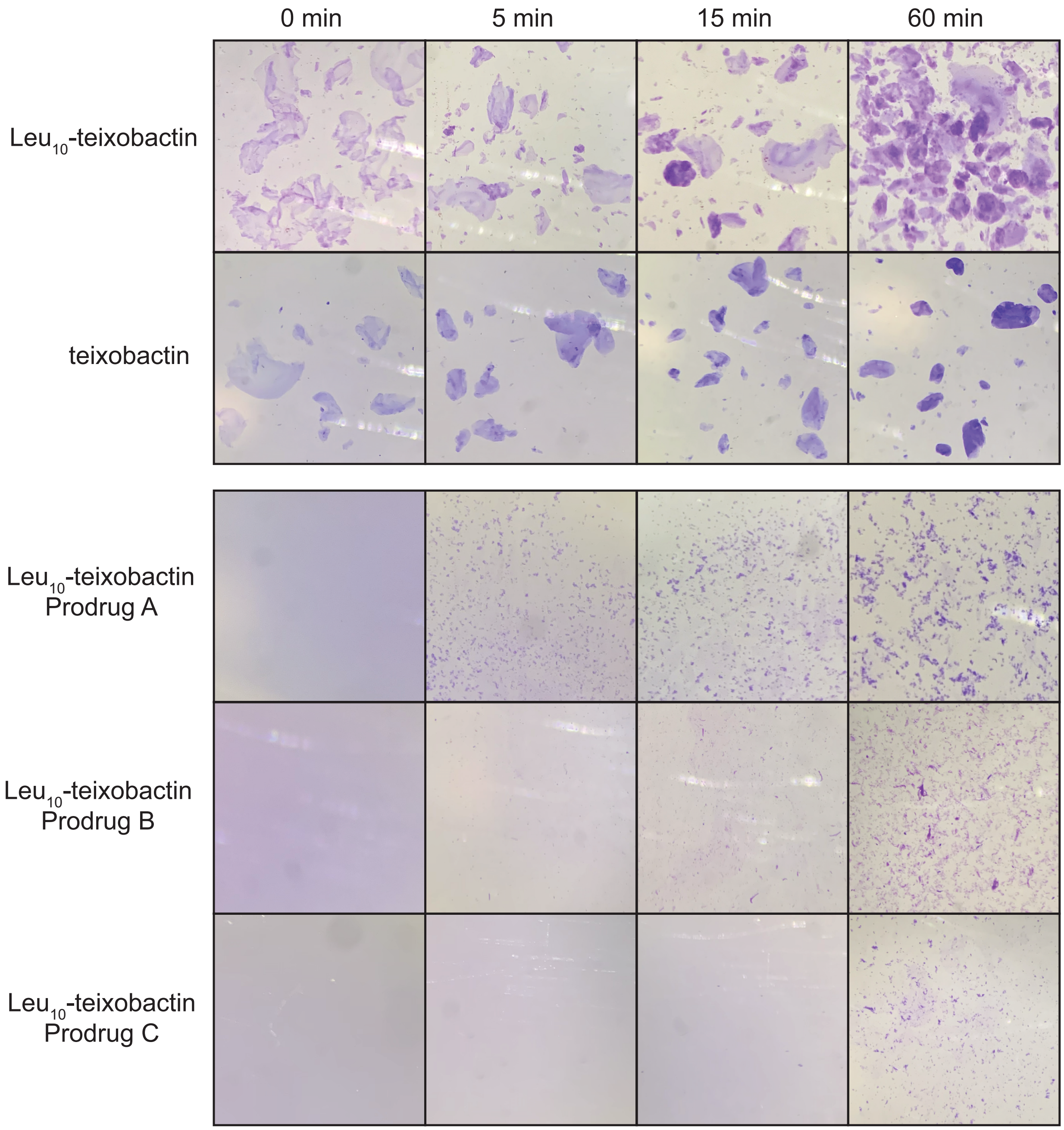

Figure S13. Gel formation of Leu$_{10}$-teixobactin and teixobactin and delayed gel formation of Leu$_{10}$-teixobactin prodrugs A, B, and C.

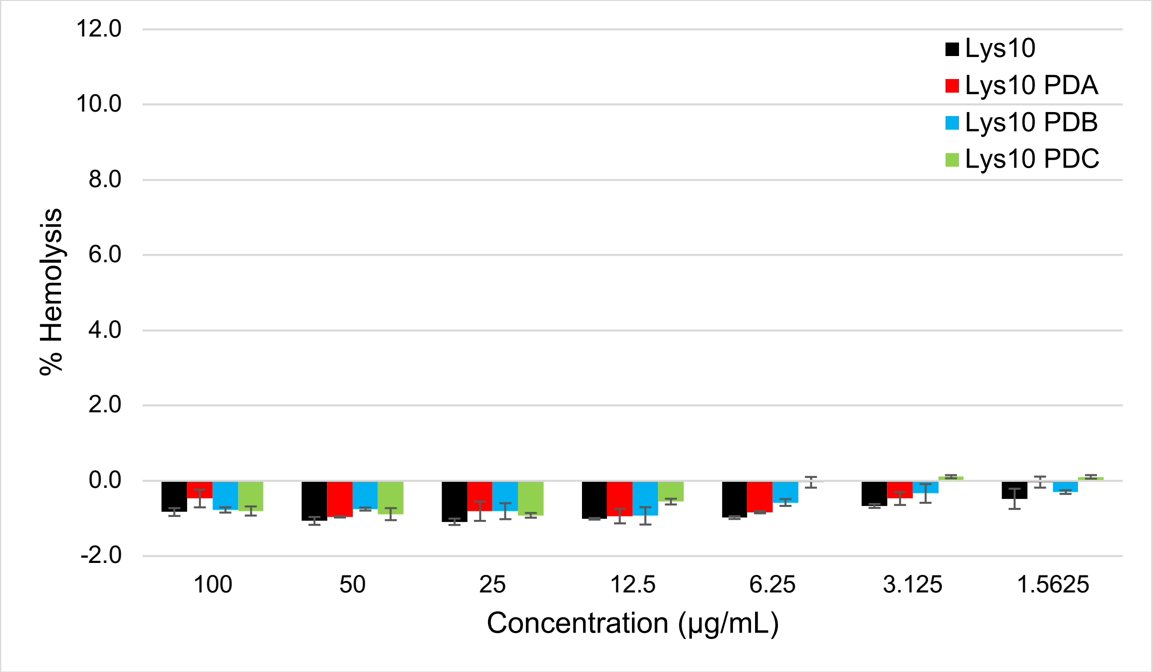

Figure S14. Hemolytic assay of Lys_{10}-teixobactin and the Lys_{10}-teixobactin prodrugs without polysorbate 80.

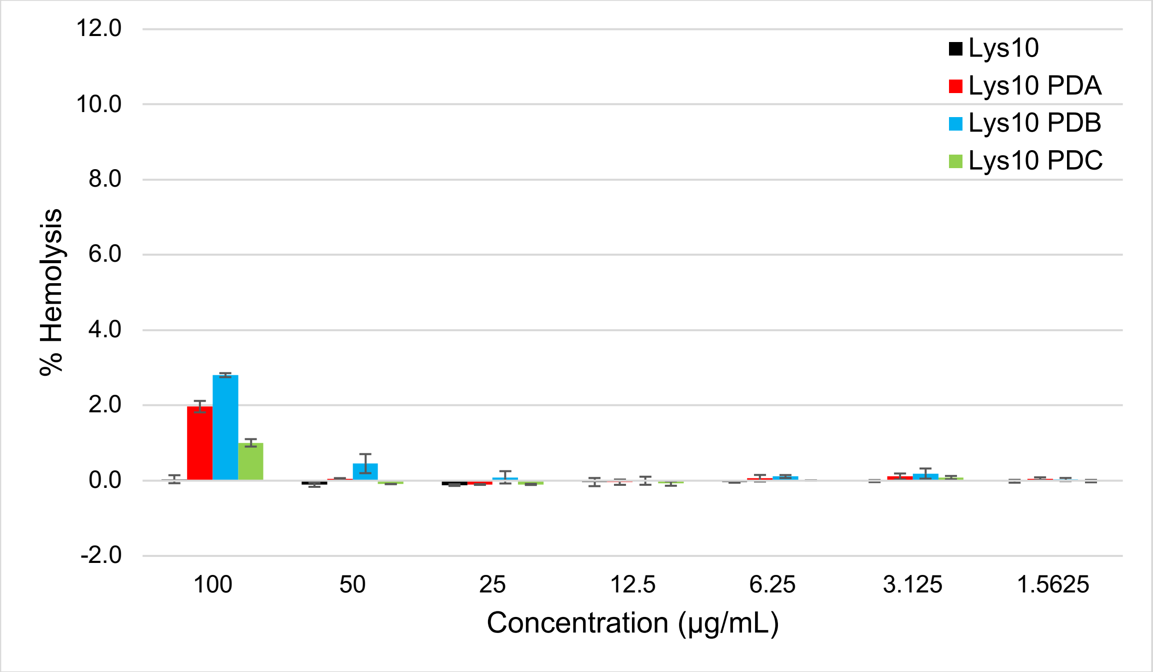

Figure S15. Hemolytic assay of Lys_{10}-teixobactin and the Lys_{10}-teixobactin prodrugs with 0.002% polysorbate 80.

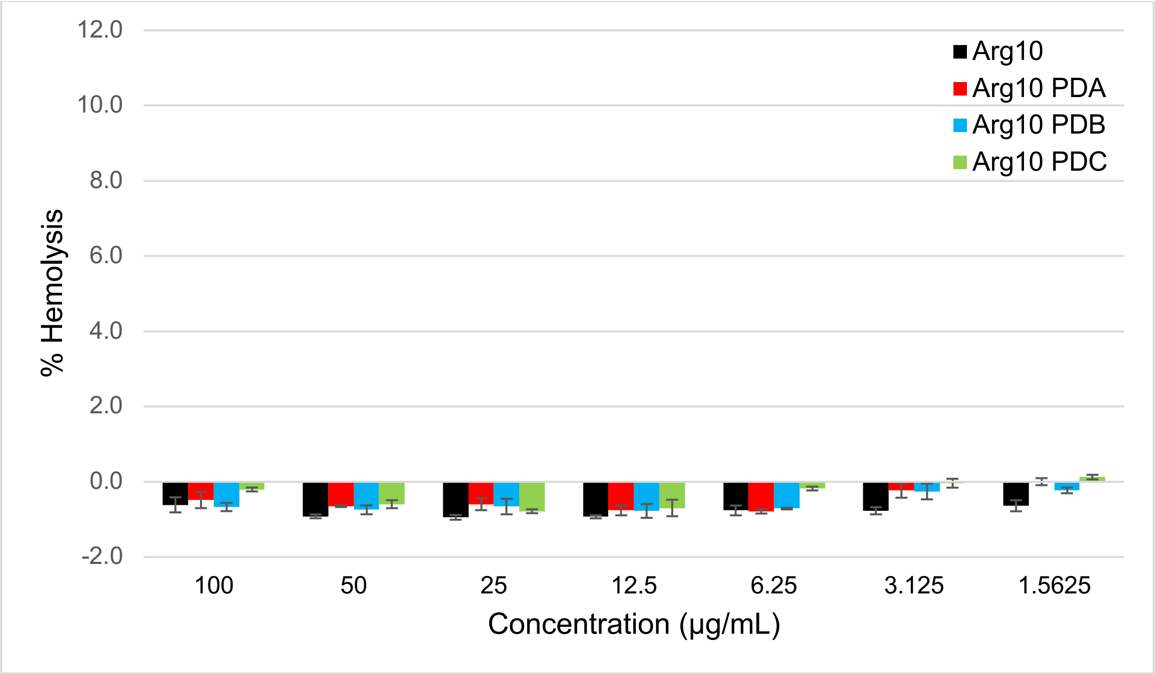

Figure S16. Hemolytic assay of Arg_{10}-teixobactin and the Arg_{10}-teixobactin prodrugs without polysorbate 80.

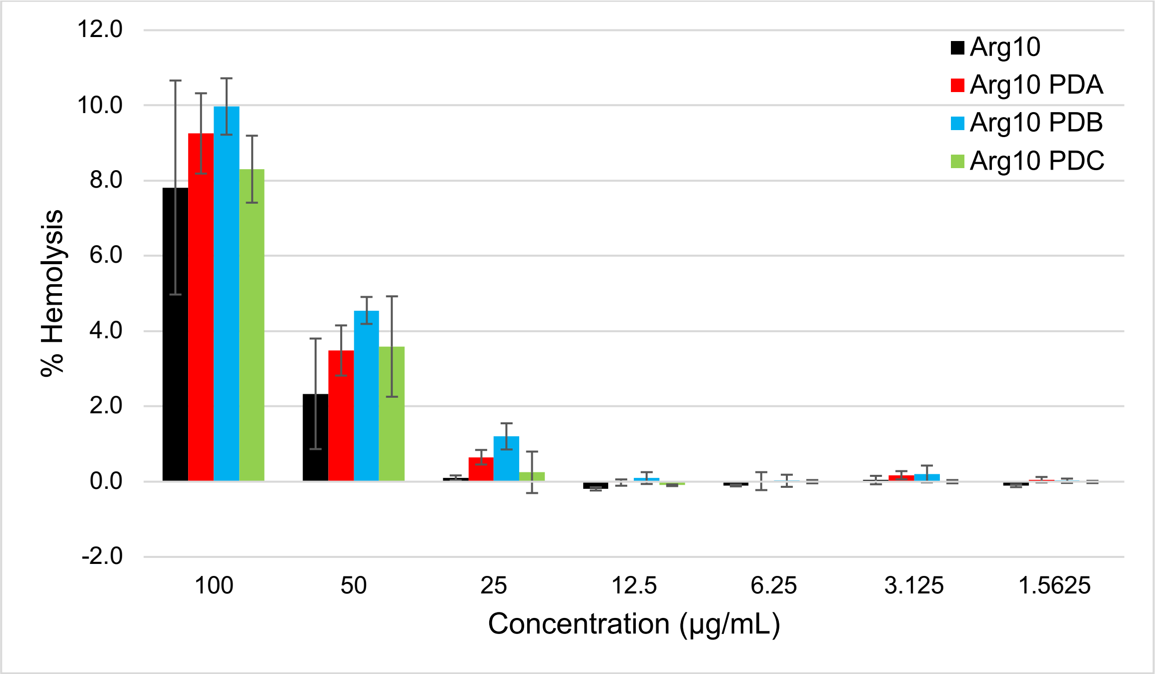

Figure S17. Hemolytic assay of Arg_{10}-teixobactin and the Arg_{10}-teixobactin prodrugs with 0.002% polysorbate 80.

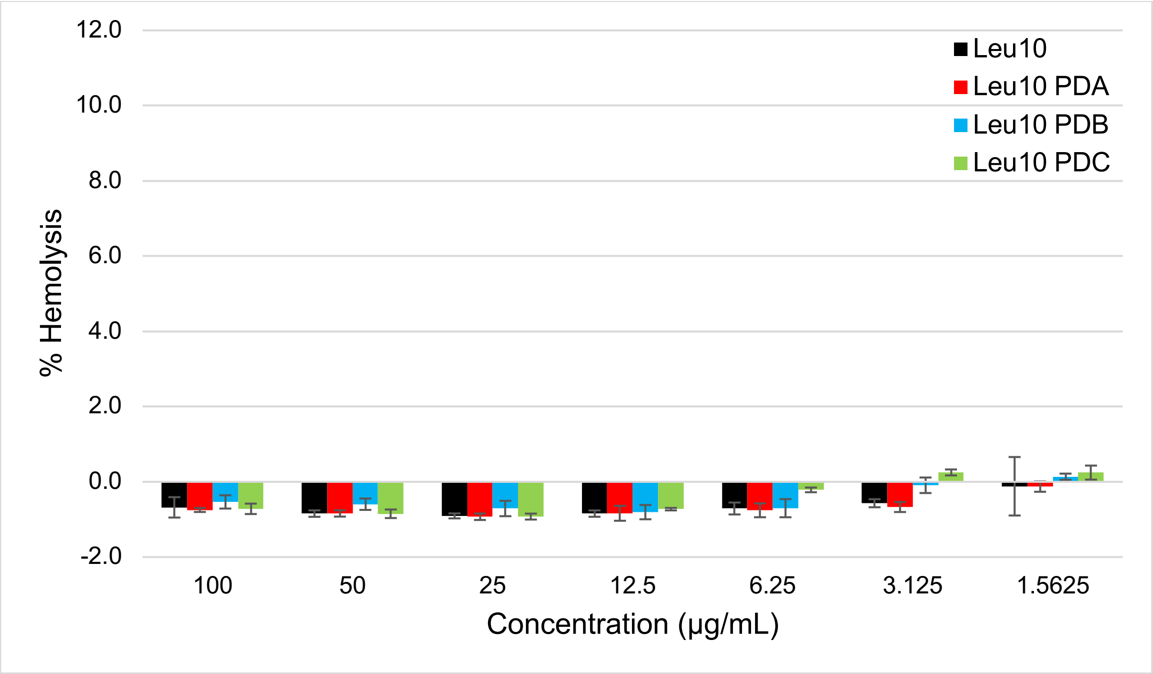

Figure S18. Hemolytic assay of Leu_{10}-teixobactin and the Leu_{10}-teixobactin prodrugs without polysorbate 80.

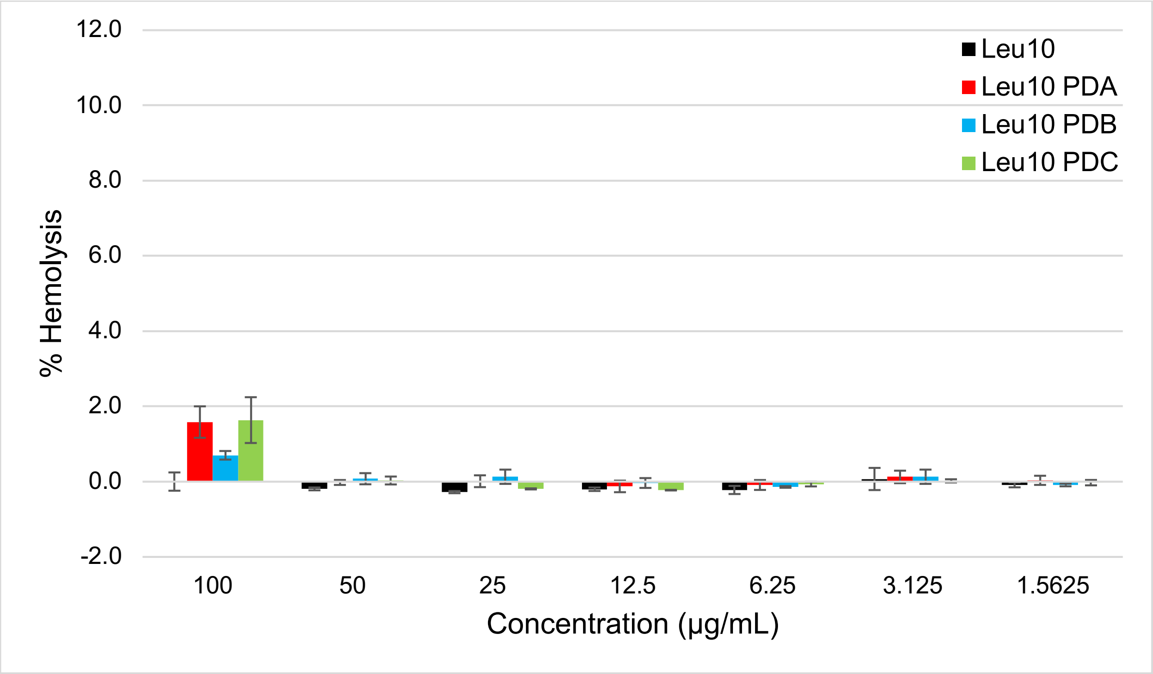

Figure S19. Hemolytic assay of Leu_{10}-teixobactin and the Leu_{10}-teixobactin prodrugs with 0.002% polysorbate 80.

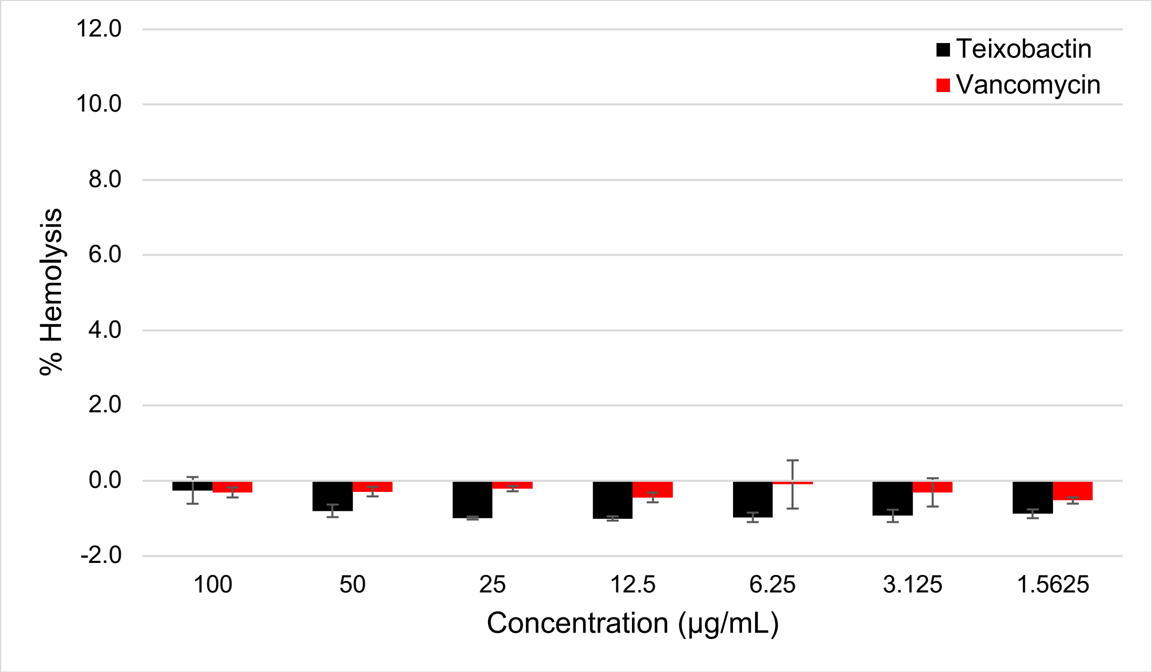

Figure S20. Hemolytic assay teixobactin and vancomycin without polysorbate 80.

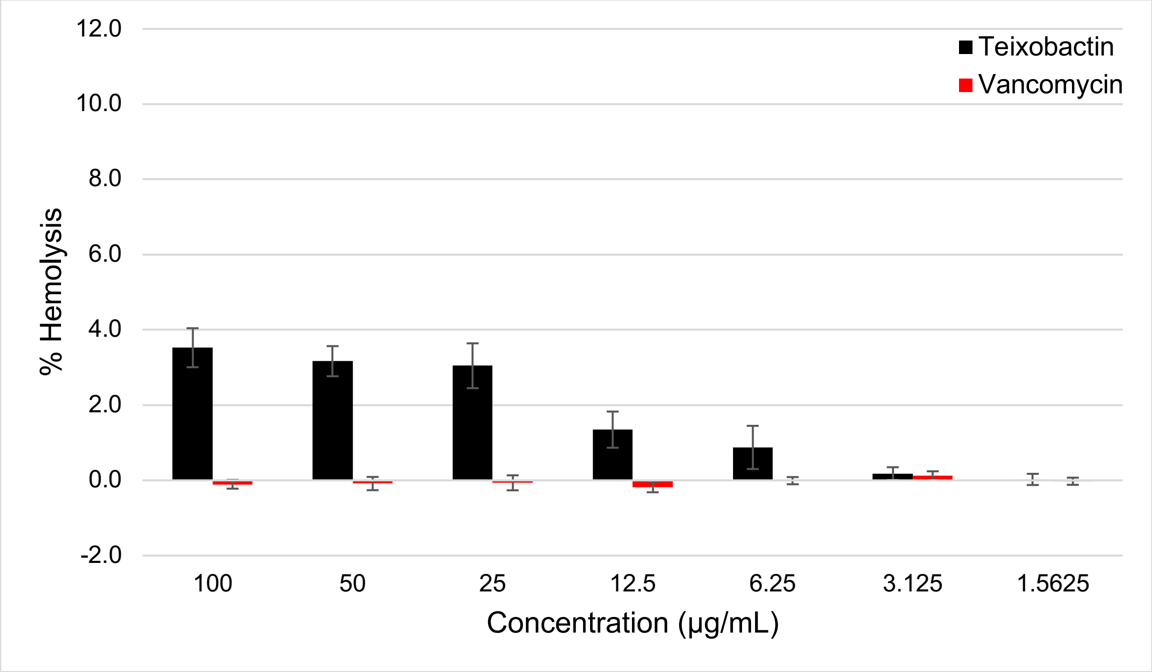

Figure S21. Hemolytic assay of teixobactin and vancomycin with 0.002% polysorbate 80.

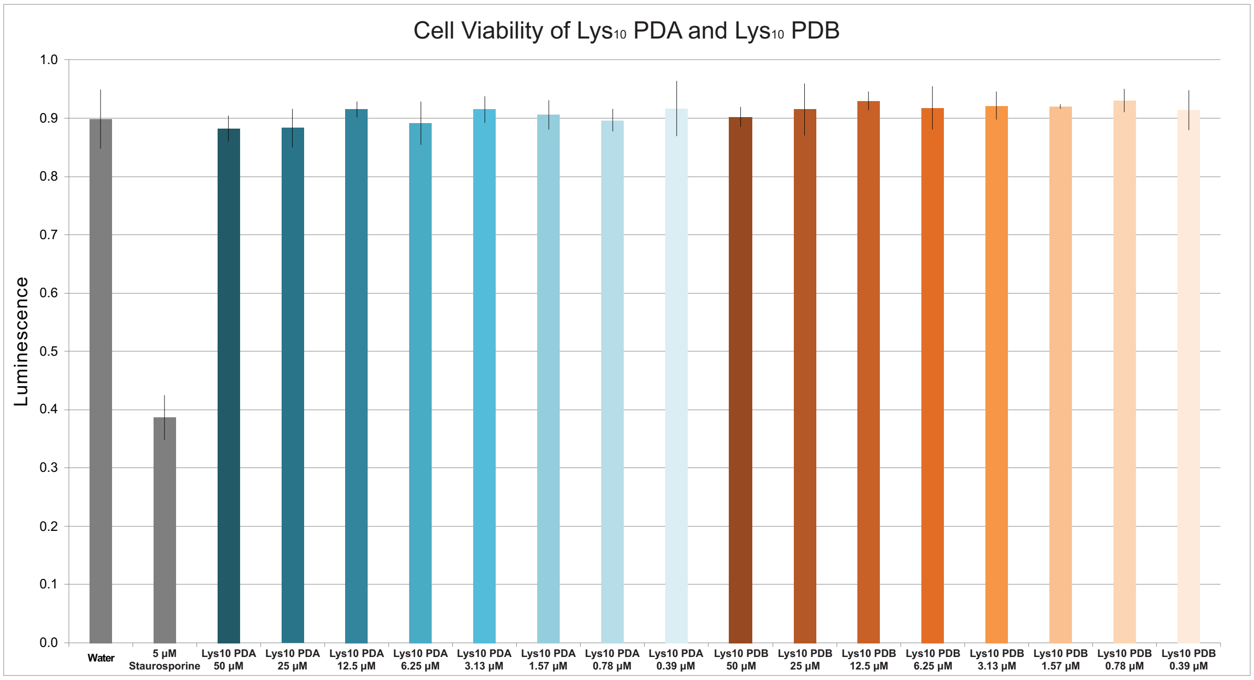

Figure S22. Cytotoxicity assay of Lys_{10}-teixobactin prodrug A and prodrug B with HeLa cells.

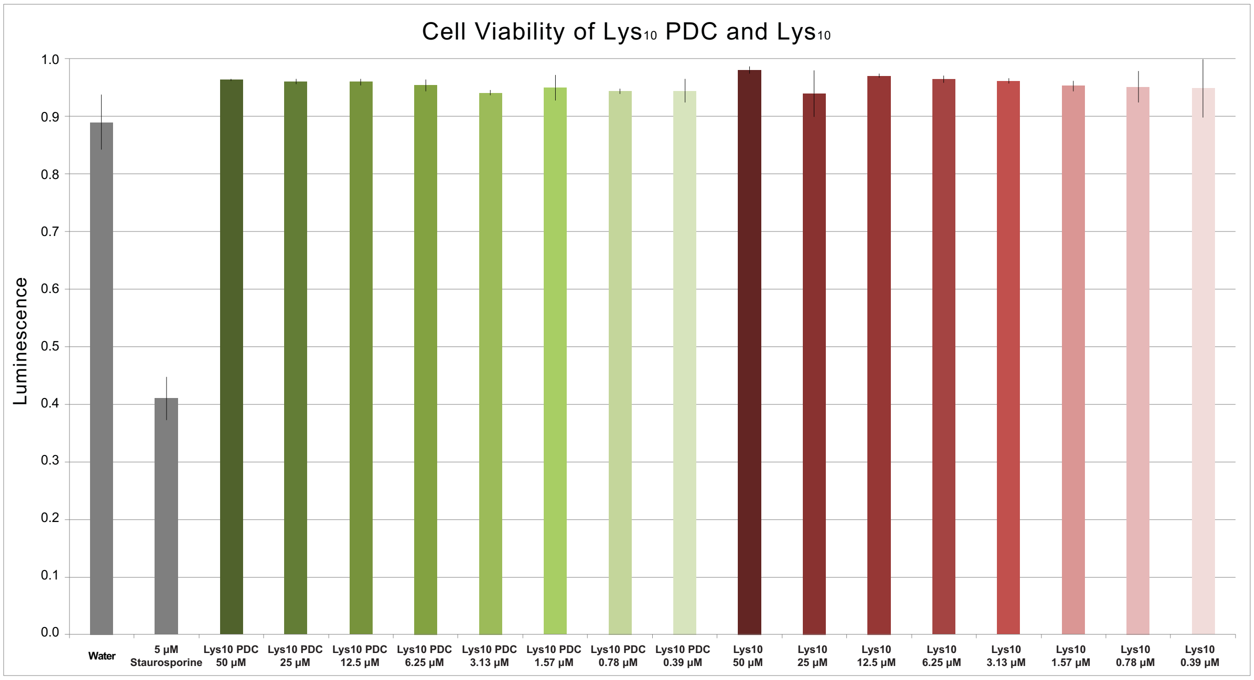

Figure S23. Cytotoxicity assay of Lys_{10}-teixobactin prodrug C and Lys_{10}-teixobactin with HeLa cells.

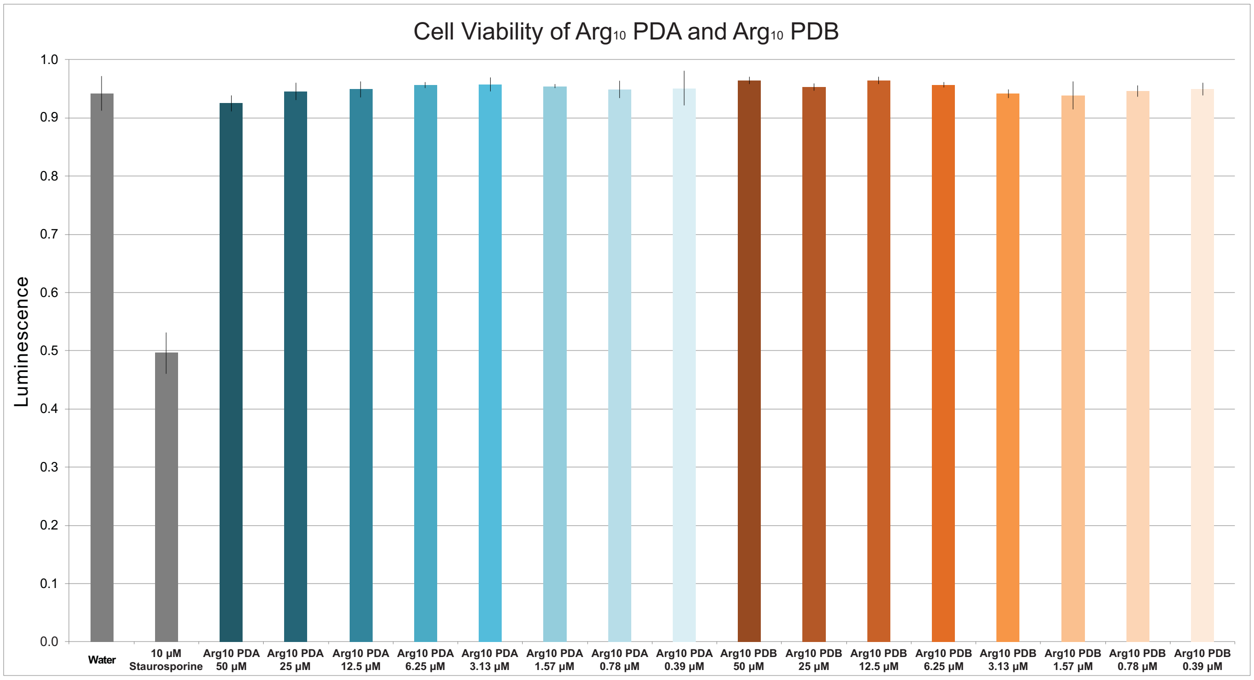

Figure S24. Cytotoxicity assay of Arg_{10}-teixobactin prodrug A and prodrug B with HeLa cells.

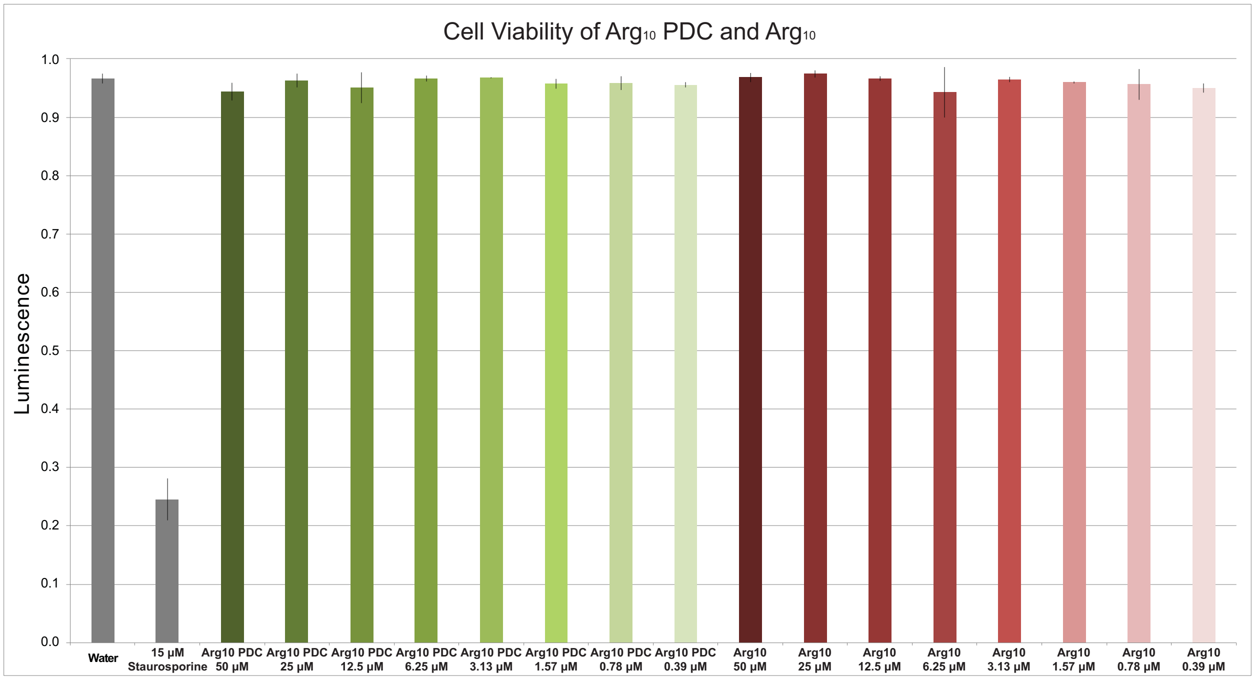

Figure S25. Cytotoxicity assay of Arg_{10}-teixobactin prodrug C and Arg_{10}-teixobactin with HeLa cells.

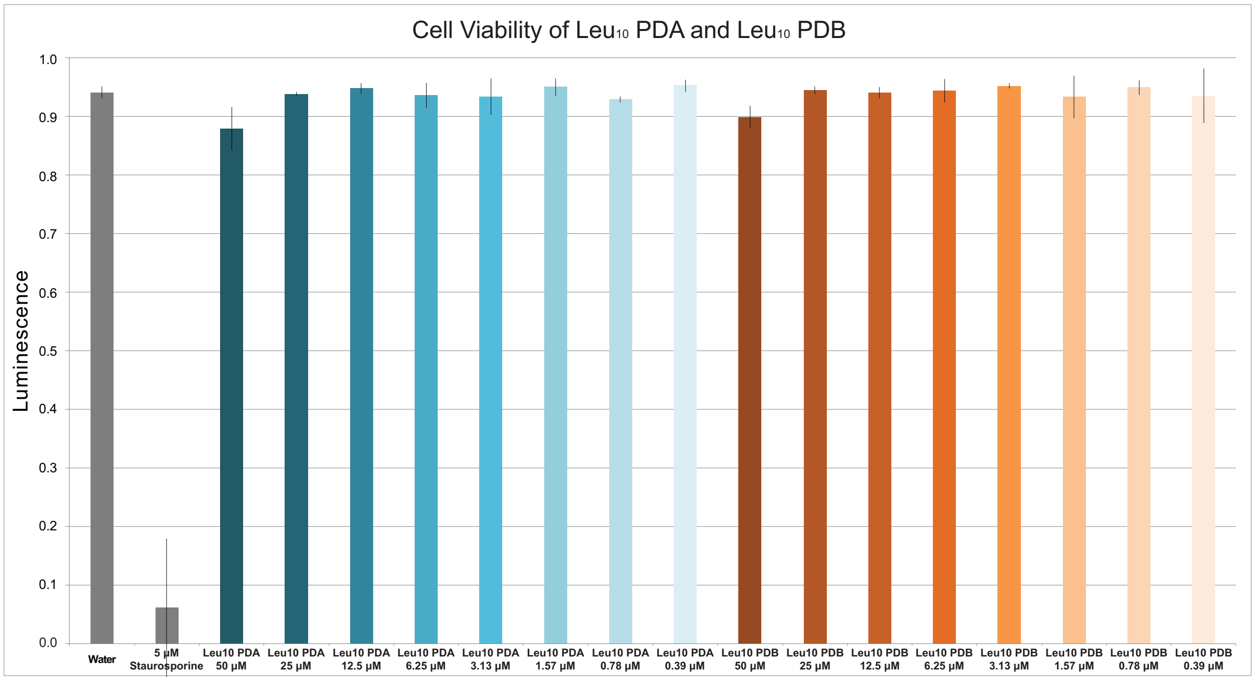

Figure S26. Cytotoxicity assay of Leu$_{10}$-teixobactin prodrug A and prodrug B with HeLa cells.

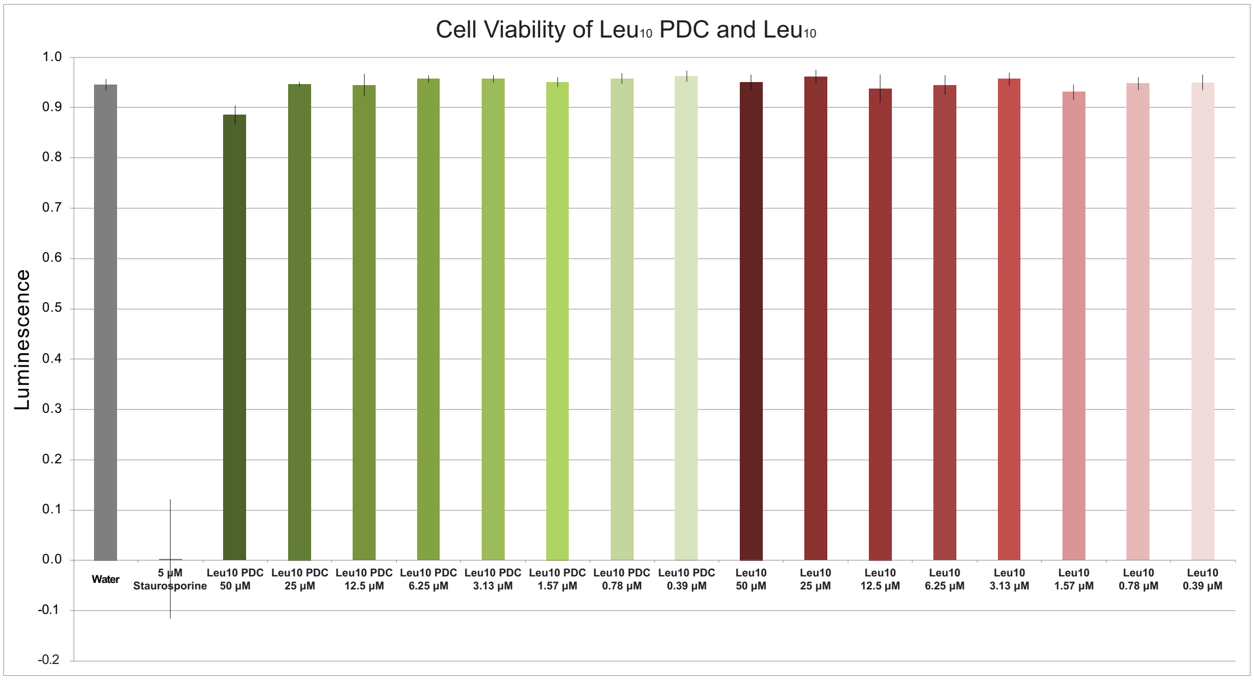

Figure S27. Cytotoxicity assay of Leu$_{10}$-teixobactin prodrug C and Leu$_{10}$-teixobactin with HeLa cells.

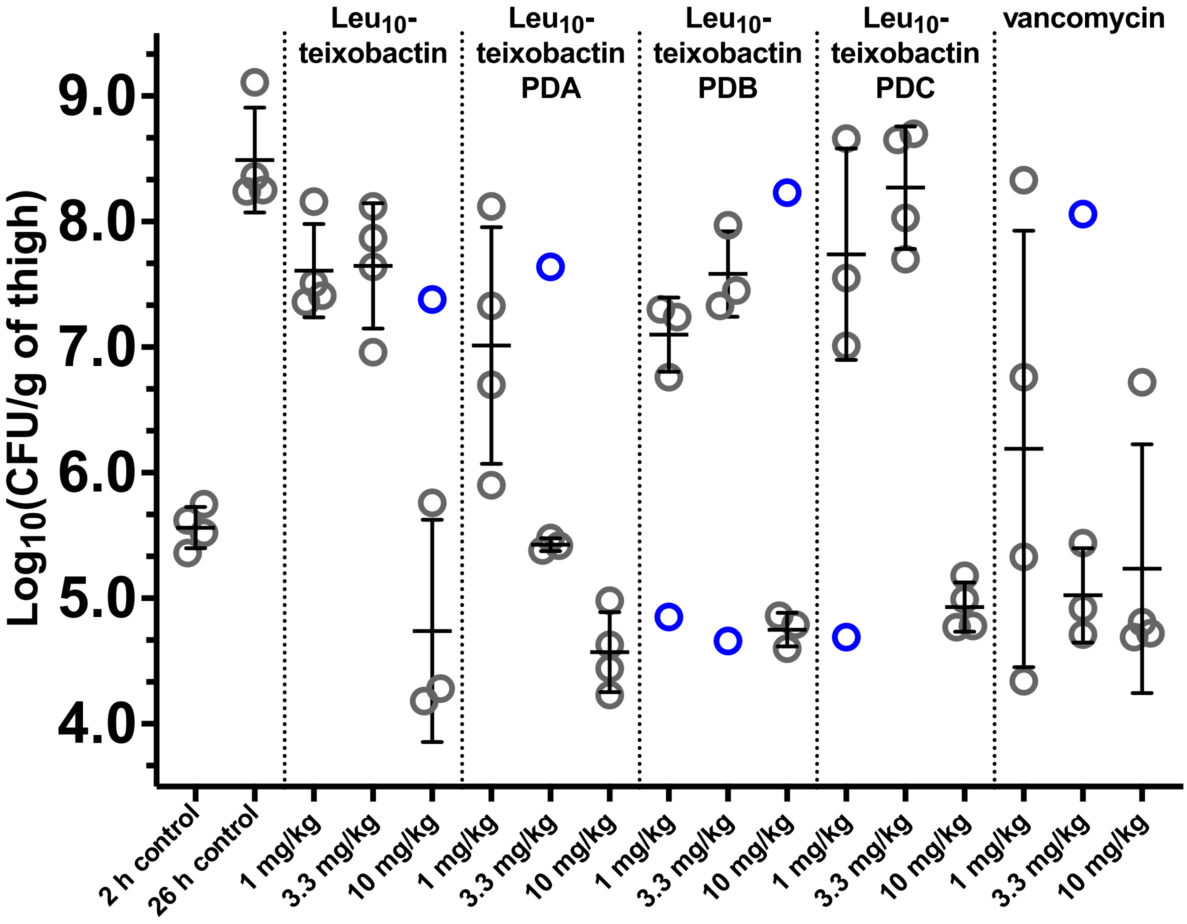

Figure S28. Graph of the complete data for the neutropenic mouse thigh infection efficacy model against MRSA (ATCC BAA-1717) of the Leu$_{10}$-teixobactin prodrugs and Leu$_{10}$-teixobactin, with vancomycin as a positive control. Data points in blue are the severe outliers that were removed from the data analysis in Figure 7 in the main text and not included in the error bar calculations for Figure 7 or Figure S28.

Materials and Methods[1]

Materials. Amino acids, coupling agents, 2-chlorotrityl chloride resin, DIC, and triisopropylsilane were purchased from Chem-Impex. Boc-Ser(Fmoc-Ile)-OH was purchased from AAPPTec. Vancomycin (hydrochloride salt) was purchased from Sigma-Aldrich. Teixobactin (hydrochloride salt) was provided as the by NovoBiotic Pharmaceuticals. DMF (amine-free), DIPEA, 2,4,6-collidine, and piperidine were purchased from Alfa-Aesar. DMAP and polysorbate 80 were purchased from Acros Organics. HPLC-grade acetonitrile, and dichloromethane were purchased from Fisher Scientific. TFA and hexafluoroisopropanol were purchased from Oakwood Chemical. Reagent-grade solvents, chemicals, amino acids, and resin were used as received, with the exception of dichloromethane, which was dried through an alumina column under argon, and DMF, which was dried through an alumina column and an amine scavenger resin column under argon.

Methods for Synthesis, Purification, and Analysis of Peptides. Solid-phase peptide synthesis was carried out manually in a solid phase reaction vessel. Analytical reverse-phase HPLC was performed on an Agilent 1260 instrument equipped with an Aeris PEPTIDE 2.6 μm XB-C18 column (Phenomonex). Preparative reverse-phase HPLC was performed on a Rainin Dynamax instrument equipped with a Zorbax SB-C18 column (Agilent) for all teixobactin analogues. All teixobactin prodrug analogues were first purified on a Biotage® Isolera™ One system equipped with a Biotage® Sfär Bio C18 – Duo 300 Å 20 μm column, before repurifying on the Rainin Dynamax instrument. UV detection (214 nm) was used for analytical and preparative HPLC. HPLC grade acetonitrile and 18 MΩ deionized water, each containing 0.1% trifluoroacetic acid, were used for analytical and preparative reverse-phase HPLC. Matrix-assisted laser desorption/ionization time-of-flight (MALDI-TOF) mass spectrometry was performed on an AB

SCIEX TOF/TOF 5800 system and α-cyano-4-hydroxycinnamic acid was used as the sample matrix. All peptides were prepared and used as the trifluoroacetate salts and were assumed to have one trifluoroacetate ion per ammonium group present in each peptide.

Synthesis of teixobactin *O*-acyl isopeptide prodrug analogues and their corresponding teixobactin analogues.

Lys_{10}-teixobactin, Arg_{10}-teixobactin, Leu_{10}-teixobactin, and all *O*-acyl isopeptide prodrug analogues were prepared as the trifluoroacetate salts by solid-phase peptide synthesis followed by solution phase cyclization, as previously described.[1,2] In the original procedure, HBTU and HOBt are used in the solution-phase cyclization step, but in the synthesis of these peptides, HATU and HOAt were used instead. For all *O*-acyl isopeptide prodrug analogues, Boc-Ser(Fmoc-Ile)-OH was coupled in place of the desired Ile and Ser residues. Syntheses on a 0.1–0.2 mmol scale afforded 5–39 mg (1.6-21%) of Lys_{10}-teixobactin, Arg_{10}-teixobactin, Leu_{10}-teixobactin and the *O*-acyl isopeptide prodrug analogues.

Representative synthesis of Lys_{10}-teixobactin prodrug A. *Resin Loading.* 2-chlorotrityl chloride resin (300 mg, 1.6 mmol/g) was added to a 10-mL Bio-Rad Poly-Prep chromatography column. The resin was suspended in dry DCM (8 mL) and allowed to swell for 10 mins. The DCM was drained and a solution of Fmoc-Lys(Boc)-OH (150 mg, 0.32 mmol, 1.8 equiv) and 2,4,6-collidine (300 μL) in dry DCM (7 mL) was added. The suspension was gently agitated for 5 h. The solution was drained, and the resin was washed with dry DCM (3X). After washing, a solution of DCM/MeOH/DIPEA (17:2:1, 8 mL) was added to the resin and agitated for 1 h to cap any unreacted 2-chlorotrityl chloride sites. The solution was drained, and the resin was washed with DCM (3X) and dried with a flow of nitrogen. The resin loading was determined to be 0.18 mmol

(0.60 mmol/g, 57% loading) based on UV analysis (290 nm) of the Fmoc cleavage product.

Solid-phase amino acid couplings. The loaded resin was suspended in dry DMF and transferred to a solid-phase peptide synthesis reaction vessel for manual peptide synthesis. Fmoc-Ala-OH, Fmoc-D-Thr-OH, Boc-Ser(Fmoc-Ile)-OH, Fmoc-D-*allo*-Ile-OH, Fmoc-D-Gln(Trt)-OH, Fmoc-Ser(t-Bu)-OH, Fmoc-Ile-OH, and Boc-*N*-methyl-D-Phe-OH were coupled through the following cycles: (1) Fmoc deprotection with 20% (v/v) piperidine in dry DMF (5 mL) for 5 min (2X), (2) resin washing with dry DMF (7X), (3) coupling of amino acid (0.72 mmol, 4.0 equiv) with HCTU (0.72 mmol, 4.0 equiv) in 20% (v/v) collidine in dry DMF (5 mL) for 30 min, and (4) resin washing with dry DMF (7X). After completing the linear synthesis, the resin was transferred to a 10-mL Bio-Rad Poly-Prep chromatography column and washed with dry DMF (3X) and DCM (3X).

Esterification. In a test tube, Fmoc-Ile-OH (630 mg, 1.8 mmol, 9.9 equiv) and diisopropylcarbodiimide (280 µL, 1.8 mmol, 10 equiv) were dissolved in dry DCM (5 mL). The solution was filtered through a 0.20-µm nylon filter into a test tube containing 4-dimethylaminopyridine (21.8 mg, 0.18 mmol, 1.00 equiv). The filtrate was transferred to the resin and gently agitated for 1 h. The solution was drained, and the resin washed with dry DCM (3X) and DMF (3X).

Fmoc deprotection of Ile_{11}. The Fmoc protecting group on Ile_{11} was removed with a solution of 20% (v/v) piperidine in DMF (5 mL) for 15 mins. The solution was drained, and the resin was washed with dry DMF (3X) and DCM (3X).

Cleavage of the linear peptide from the resin. The linear peptide was cleaved from resin by subjecting the resin to a cleavage solution of 20% (v/v) HFIP in dry DCM (7.5 mL) and agitating for 1 h. The filtrate was collected in a 250-mL round-bottom flask. The HFIP treatment

was repeated for 30 mins and the filtrate was added to the first in the round-bottom flask. The resin was washed with dry DCM (3X). The combined filtrates and DCM washes were concentrated under reduced pressure to afford a colorless oil.

Solution-phase cyclization. The oil was dissolved in DMF (125 mL) in the same 250 round-bottom flask as the previous step. HATU (410 mg, 1.1 mmol, 6.0 equiv) and HOAt (150 mg, 1.1 mmol, 6.1 equiv) were added to the solution. The reaction mixture was then stirred under nitrogen for 10 mins. DIPEA (100 µL, 0.6 mmol, 3.2 equiv) was added dropwise to the solution and the mixture was stirred under nitrogen at room temperature for 12 h. The reaction mixture was concentrated under reduced pressure to afford the cyclized peptide as a yellow solid. The solid was placed under vacuum (≤60 mTorr) to remove any residual solvents.

Global Deprotection and Ether Precipitation. The crude protected peptide was dissolved in a mixture of TFA/TIPS/H_2O (90:5:5, 10 mL), and the solution was stirred for 1.5 h. The deprotection mixture was transferred to two 50-mL conical tubes, each containing 35 mL ice-cold diethyl ether, with a precipitate forming immediately. The 50-mL conical tubes were centrifuged (2500 x g) for 10 min to pellet the crude peptide. The diethyl ether supernatant was decanted into a 125-mL Erlenmeyer flask. This process was repeated 2X, adding additional ice-cold ether followed by centrifugation and decantation. The pellet was then dried under nitrogen.

Purifications. The dried peptide pellet was dissolved in 10% (v/v) MeCN in H_2O (10 mL) and purified on a Biotage® Isolera™ One system equipped with a Biotage® Sfär Bio C18 – Duo 300 Å 20 µm column using a H_2O/MeCN (10%-55%) gradient. The fractions were analyzed by MALDI-TOF and analytical HPLC. Fractions containing the desired peptide were combined and lyophilized for repurification. The lyophilized material from the first purification were dissolved in 20% (v/v) MeCN in H_2O (4 mL) and purified by reverse-phase HPLC with H_2O/MeCN

(gradient elution of 20-40% with 0.1% TFA over 120 min) on a C18 column. Fractions were analyzed by MALDI-TOF and analytical HPLC. The pure fractions were combined and lyophilized to give 39 mg (14% yield based on resin loading) of Lys_{10}-teixobactin prodrug A trifluoroacetate (TFA) salt as a white powder.

Table S1. Yields of purified teixobactin *O*-acyl isopeptide prodrug analogues and their corresponding teixobactin analogues.

Teixobactin Analogue	Yield (mg)	% Yield	Calculated MW as TFA salt
Lys_{10}-teixobactin	7	2.1%	1443.7 (•2 TFA)
Lys_{10}-teixobactin Prodrug A	39	14%	1557.7 (•3 TFA)
Lys_{10}-teixobactin Prodrug B	9	5.4%	1557.7 (•3 TFA)
Lys_{10}-teixobactin Prodrug C	7	3.2%	1671.7 (•4 TFA)
Arg_{10}-teixobactin	7	2.6%	1471.7 (•2 TFA)
Arg_{10}-teixobactin Prodrug A	20	15%	1585.7 (•3 TFA)
Arg_{10}-teixobactin Prodrug B	9	6.6%	1585.7 (•3 TFA)
Arg_{10}-teixobactin Prodrug C	21	21%	1699.7 (•4 TFA)
Leu_{10}-teixobactin	8	4.1%	1314.7 (•1 TFA)
Leu_{10}-teixobactin Prodrug A	18	10%	1428.7 (•2 TFA)
Leu_{10}-teixobactin Prodrug B	5	1.6%	1428.7 (•2 TFA)
Leu_{10}-teixobactin Prodrug C	10	6.0%	1542.7 (•3 TFA)

Conversion kinetics studies of teixobactin *O*-acyl isopeptide prodrug analogues at room temperature and 37 °C. A 1 mL analytical HPLC vial was charged with 300 µL of 50 mM phosphate buffer followed by 300 µL of a 1 mg/mL stock solution of the peptide in H_2O. An aliquot was then immediately injected onto an Agilent 1260 instrument equipped with an Aeris PEPTIDE 2.6 µm XB-C18 column (Phenomonex). Additional aliquots were injected every 24 minutes for 4 h. Each sample was run on a gradient of 5–67% acetonitrile over 15 min, with monitoring of absorbance at 214 nm. The temperature in the HPLC sample chamber was recorded. Conversion kinetics of all reactions were run at 23–25 ± 1 °C. Peaks corresponding to the prodrug analogue, intermediates (prodrugs C only), and the teixobactin analogue product were integrated using the Agilent software, and the relative areas were recorded for kinetic analysis.

A 1 mL analytical HPLC vial was charged with 400 µL of 100 mM sodium phosphate

buffer at pH 7.4. To a second analytical HPLC vial, 400 μL of a 1 mg/mL stock solution of the peptide in H_2O was added. Both vials were placed in a water bath set to 37 °C and allowed to preheat for ca. 15 min. To an additional vial in the 37 °C water bath, 300 μL of the sodium phosphate buffer and 300 μL of the 1 mg/mL stock solution of peptide were added. Aliquots of 100 μL were removed every 5 minutes (5, 10, 15, and 20) into a low-volume analytical HPLC vials and placed immediately on dry ice. After all aliquots were taken, the aliquots were analyzed on the same HPLC instrument, using the same gradient and wavelength as the conversion studies at room temperature (described above). Each sample was thawed immediately before injection but kept at a low enough temperature to minimize further conversion of the prodrug analogues. Peaks corresponding to the prodrug analogue, intermediates (prodrugs C only), and the teixobactin analogue product were integrated using the Agilent software, and the relative areas were recorded for kinetic analysis.

MIC assays of teixobactin *O*-acyl isopeptide prodrug analogues and their corresponding teixobactin analogues. *Bacillus subtilis* (ATCC 6051), *Staphylococcus epidermidis* (ATCC 14990), *Staphylococcus aureus* (ATCC 29213), and *Escherichia coli* (ATCC 10798) were cultured from glycerol stocks in Mueller-Hinton broth overnight in a shaking incubator at 37 °C. *Staphylococcus aureus* (ATCC 700698) was cultured from a glycerol stock in brain heart infusion broth overnight in a shaking incubator at 37 °C. An aliquot of a 1 mg/mL antibiotic stock solution in DMSO was diluted with appropriate culture media to make a 64 μg/mL solution. A 200-μL aliquot of the 64 μg/mL solution was transferred to a sterile, untreated 96-well plate. Two-fold serial dilutions were made with media across a 96-well plate to achieve a final volume of 100 μL in each well. These solutions had the following concentrations: 64, 32, 16, 8, 4, 2, 1, 0.5, 0.25, 0.125, and 0.0625 μg/mL. The overnight cultures of each bacterium were diluted

with Mueller-Hinton broth to an OD_{600} of 0.075 as measured for 200 µL in a 96-well plate. The diluted mixture was further diluted to a 1×10^6 CFU/mL with Mueller-Hinton media. A 100-µL aliquot of the 1×10^6 CFU/mL bacterial solution was added to each well in the 96-well plates, resulting in final bacteria concentrations of 5×10^5 CFU/mL in each well. As 100-µL of bacteria were added to each well, the teixobactin analogues and teixobactin prodrug analogues were also diluted to the following concentrations: 32, 16, 8, 4, 2, 1, 0.5, 0.25, 0.125, 0.0625, and 0.03125 µg/mL. The plate was covered with a lid and incubated at 37 °C for 16 h. The OD_{600} were measured using a 96-well UV/vis plate reader (MultiSkan GO, Thermo Scientific). The MIC values were taken as the lowest concentration that had no bacteria growth. Each MIC assay was run in quadruplicate (technical replicates). MIC assays were performed in test media without polysorbate 80 or containing 0.002% polysorbate 80. For MIC assays performed with 0.002% polysorbate 80, the antibiotic stock solution was diluted with appropriate culture media to make a 16 µg/mL solution. Several of the MIC assays were repeated to ensure reproducibility.

Figure S29. MIC assay plate layout.[9]

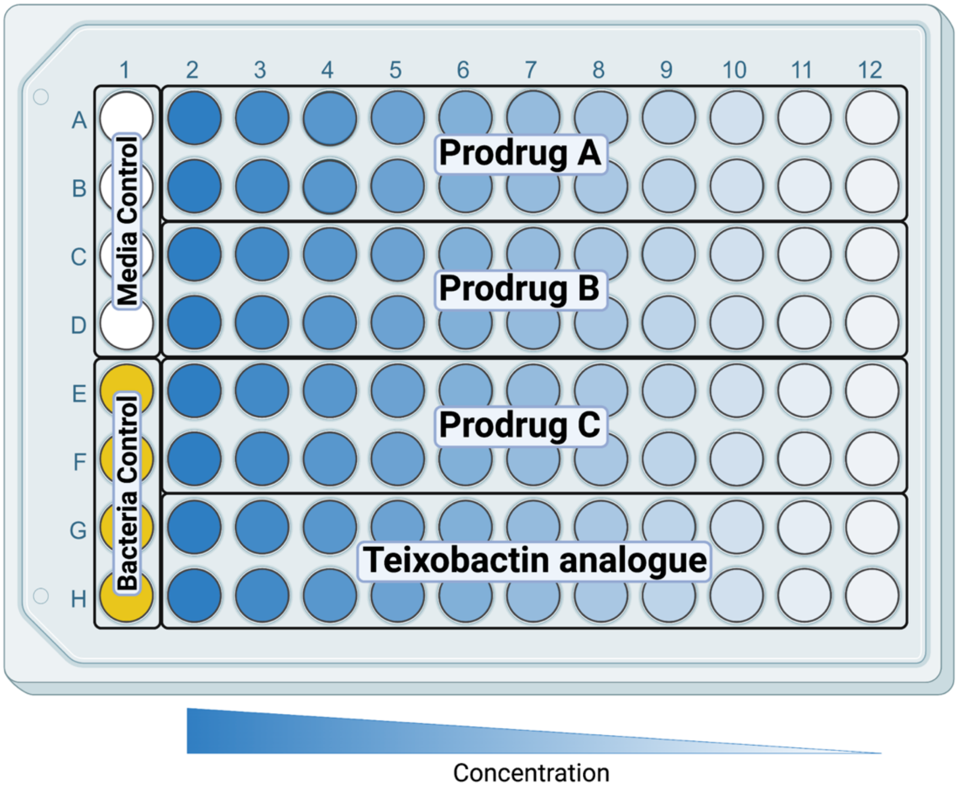

Gel formation studies of teixobactin *O*-acyl isopeptide prodrug analogues and their corresponding teixobactin analogues.[3] A small amount of crystal violet was added to 1X PBS buffer at pH 7.4. The buffer was centrifuged to pellet any undissolved crystal violet. A 20 µL drop of the PBS buffer was placed onto a glass depression well microscope slide. A 1-µL aliquot of the 10 mg/mL peptide stock solution in DMSO was added to the drop of PBS buffer. The mixture was stirred with the pipet tip to form a homogenous solution. A low magnification stereoscopic microscope was used to visually observe the drop. Gel formation was observed and photographed over the course of 60 minutes with stirring of the drop every 10–15 mins.

Hemolytic assay of teixobactin *O*-acyl isopeptide prodrug analogues and their corresponding teixobactin analogues.[4,5] *Preparation of Phosphate-Buffered Saline (PBS) Buffers.* A 10X PBS buffer was prepared by dissolving 8.9 g of Na_2HPO_4, 1.2 g KH_2PO_4, 40 g

NaCl, and 1 g KCl in 500 mL of 18 MΩ deionized water. The solution was stirred until the buffer salts were completely dissolved. The pH of the 10X PBS buffer was adjusted to 7.4 using either 1 M HCl or 1 M NaOH and was subsequently sterile filtered. To create a 1X PBS buffer, the 10X PBS buffer was diluted 10-fold using 18 MΩ deionized water. Another 1X PBS buffer was made, supplemented with 0.002% polysorbate 80.

Preparation of human red blood cells. Whole human blood was stored in a 4 °C in K2 EDTA to prevent coagulation. On the day of cell treatment, the blood was centrifuged at 800 x g for 5 min at 4 °C to isolate red blood cells (RBCs). The plasma layer was then removed and discarded. Approximately 3 mL of 150 mM NaCl solution was added to the RBCs and mixed gently by inversion. The RBCs were centrifuged at 800 x g for 8 min at 4 °C and the supernatant was discarded. An additional wash with 150 mM NaCl was performed, centrifuged at 800 x g for 8 min at 4 °C and the supernatant was discarded. 2 mL of whole RBCs were transferred to a 15-mL conical tube. Approximately 4 mL of 1X PBS was added to the RBCs and inverted gently to mix. The RBCs were centrifuged at 800 x g for 8 min at 4 °C. The supernatant was discarded, and the cells were washed 2–3 more times to ensure that the supernatant was visibly transparent and free of any color from pre-existing lysed RBCs. After the PBS washes, a 5% v/v RBC suspension was prepared by adding 500 μL of the RBCs to 9.5 mL of the desired 1X PBS.

Hemolytic assay procedure. Experiments were performed in triplicate (three technical replicates) in untreated V-bottom 96-well plates. An aliquot of a 1 mg/mL antibiotic stock solution in H_2O was diluted with the proper 1X PBS to make a 200 μg/mL solution. A 100-μL aliquot of the 200 μg/mL solution was transferred to a V-bottom 96-well plate. Two-fold serial dilutions were made with the desired 1X PBS down the V-bottom 96-well plate to achieve a final volume of 50 μL in each well. These solutions had the following concentrations: 200, 100, 50, 25, 12.5,

6.25, 3.125 μg/mL. The final row was used for controls, with each well receiving a 50-μL aliquot of the appropriate control. Four wells were used for a positive control with 4% Triton X-100 solution in 1X PBS. Four wells were used for a peptidic positive control with a 2.5 μM melittin solution in 1X PBS. Four wells were used for a vehicle control with 0.98X PBS (1X PBS diluted with 2% 18 MΩ deionized water). A 50-μL aliquot of the 5% RBC suspension was added to each well in the V-bottom 96-well plates. After addition of the RBCs to each well, the concentrations of the peptides were: 100, 50, 25, 12.5, 6.25, 3.125, and 1.5625 μg/mL, and the concentrations of the controls were 2% Triton X-100 and 1.25 μM melittin. The plates were sealed with a Axygen AxySeal Sealing Film and incubated at 37 °C for 1 h.

Hemolytic assay readout. A replica plate was prepared by adding a 50-μL aliquot of 1X PBS to all wells of a flat-bottomed 96-well plate. After the 1 h incubation period, the V-bottom 96-well plate was centrifuged at 1000 x g for 10 min at 4 °C to pellet the RBCs. A 50-μL aliquot of the supernatant from each well was transferred to the replica plate. The transfer was performed quickly, but very carefully to not disturb the RBC pellet. [If any RBCs were disturbed, the V-bottom 96-well plate should be centrifuged again to re-pellet the RBCs.] The final volume of each well in the flat-bottom 96-well plate was 100 μL. The OD_{540} of each well was measured using a 96-well UV/vis plate reader (MultiSkan GO, Thermo Scientific). The data were processed by comparing those values to the Triton X-100 controls and vehicle controls:

$$\% \text{ hemolytic activity} = [(A_{540})_{\text{compound}} - (A_{540})_{\text{vehicle}}] / [(A_{540})_{\text{triton}} - (A_{540})_{\text{vehicle}}] \times 100$$

Figure S30. Hemolytic assay plate layout.[9]

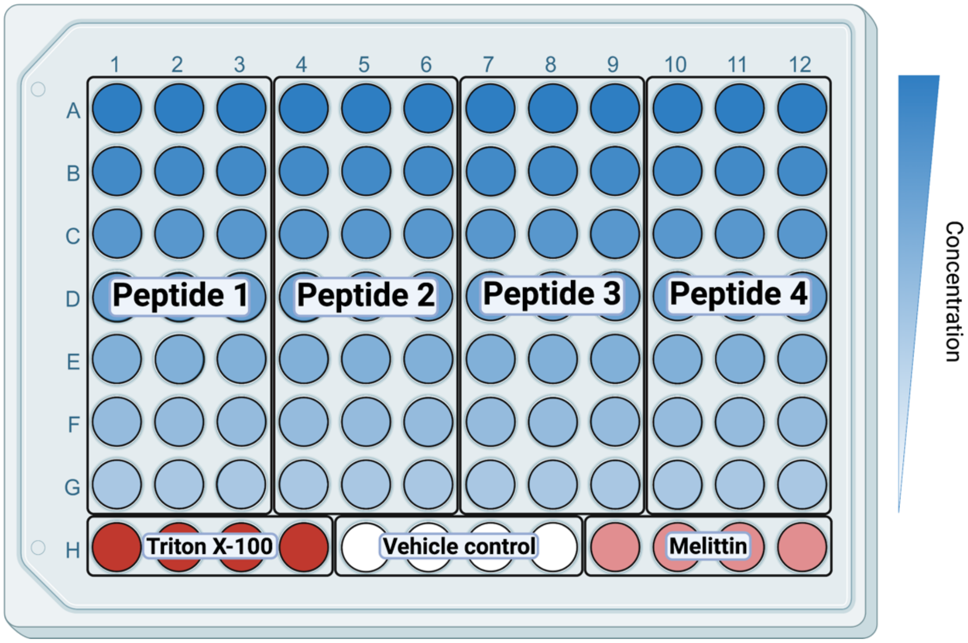

Cell culture and cytotoxicity assays of teixobactin *O*-acyl isopeptide prodrug analogues and their corresponding teixobactin analogues. *Cell culture.* HeLa cell cultures were maintained in complete media of Eagle's Minimum Essential Medium (EMEM) supplemented with 10% heat-inactivated fetal bovine serum (FBS), 100 μg/mL penicillin, and 100 μg/mL streptomycin at pH 7.4 in a humidified 5% CO_2 atmosphere at 37 °C using a Fischer Scientific Forma Series 3 Water Jacketed CO_2 Incubator. All experiments were performed in triplicate in sterile half-area 96-well plates that were cell-culture treated.

Plating cells. HeLa cells were seeded at 2,500 cells per well in the inner 60 wells of half-area 96-well plates to a total volume of 50 μL using complete media. The outer wells of the plate were filled with 100 μL of EMEM without any cells. The plates were incubated in a 5% CO_2 atmosphere at 37 °C for 24 h after plating. Prior to treatment with peptide, the media was removed by pipet from the cells.

Treatment of cells with peptide. An aliquot of a 1 mg/mL antibiotic stock solution in H_2O

was diluted with EMEM to make a 50 µM solution. A 100-µL aliquot of the 50 µM solution was transferred to the sterile, half-area 96-well plate. Two-fold serial dilutions were made with EMEM across a 96-well plate to achieve a final volume of 50 µL in each well. Each treatment was run in triplicate (technical replicates). An additional six wells were used as controls. Three wells received 50 µL of a 7% solution of 18 MΩ deionized water in EMEM (vehicle control) and the other three wells received 50 µL of either a 5, 10, or 15 µM staurosporine in EMEM solution (positive control). The plates were then incubated in a 5% CO_2 atmosphere at 37 °C for 48 h.

Cytotoxicity assay plate readout. The CytoTox-Glo Assay (CytoTox-Glo™ Cytotoxicity Assay, Promega) was performed according to manufacturer's instructions. Luminescence was measured using a microplate reader (GloMax(R) Discover System, Promega).

Figure S31. Cytotoxicity assay plate layout. All outer wells are filled with media.[9]

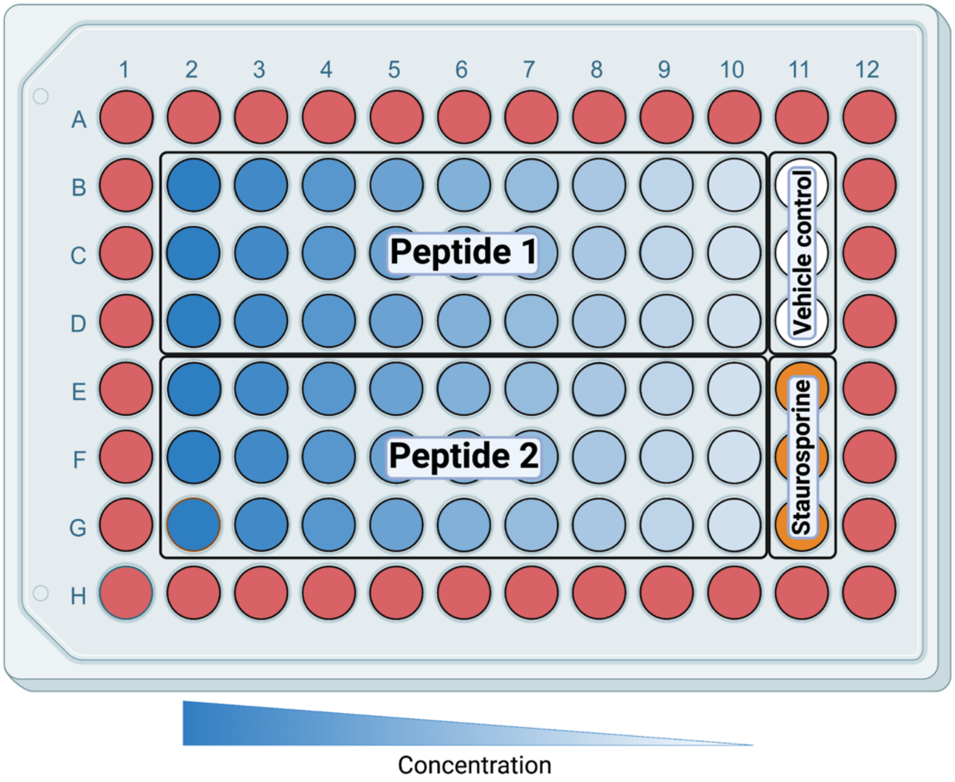

Conversion of the peptide TFA salts to HCl salts.[6] Leu_{10}-teixobactin and Leu_{10}-teixobactin prodrugs A, B, and C were converted from the trifluoroacetate (TFA) salts to the hydrochloride (HCl) salts for use in *in vivo* studies by lyophilization three times in 100 mM aqueous HCl in 18 MΩ deionized water. The lyophilized peptides (ca. 15 mg) as the TFA salts were dissolved in a 100 mM aqueous HCl (ca. 10 mL) and allowed to incubate for 5 min. The solutions were then frozen in liquid nitrogen and lyophilized. The treatment with 100 mM aqueous HCl and lyophilization was repeated two additional times. Finally, the peptides were lyophilized from 18 MΩ deionized water to remove excess HCl.

Neutropenic mouse thigh infection efficacy study against MRSA.[7,8] All *in vivo* efficacy experiments were performed at NeoSome Life Sciences, LLC. NeoSome maintains both PHS/OLAW assurance (D16-00934) and USDA certification (14-R-0215). NeoSome follows all regulatory guidelines and conforms to established NIH animal research guidelines. All animal research is reviewed by the NeoSome IACUC committee.

Female CD-1 mice were rendered neutropenic by cyclophosphamide on days -4 and -1 with 150 mg/kg and 100 mg/kg, delivered IP, respectively. *S. aureus* strain ATCC BAA-1717 was prepared for infection from an overnight plate culture. A portion of the plate was resuspended in sterile saline and adjusted to an OD of 0.10 at 625 nm. The adjusted bacterial suspension was further diluted to the predetermined infection inoculum of $1.0x10^5$ CFU/mouse thigh. Plate counts of the inoculum were performed to confirm inoculum concentration, the actual inoculum size was $1.05x10^5$ CFU/mouse thigh. Mice were infected with 100 μL of the prepared bacterial inoculum into the right thigh muscle. A total of 68 mice were infected (four mice per group). At 2 and 14 h post-infection, mice received treatment with Leu_{10}-teixobactin or the Leu_{10}-teixobactin prodrugs (as the HCl salts) at 1, 3.3, or 10 mg/kg in D5W (5% dextrose in water) administered via IV tail

vein injection in volumes of 10 mL/kg. Another group of four mice received treatment with vancomycin following the same dosing strategy.

At 2 and 26 h post-infection groups of 4 mice were euthanized. The right thighs from each animal were aseptically explanted, weighed, and homogenized to a uniform consistency. Homogenized samples were serially diluted and plated on bacterial growth media for CFU determination. CFUs were enumerated after overnight incubation at 37 °C. The CFUs were adjusted for dilution and tissue weight for each thigh with averages and standard deviation calculated for each group. Severe outliers were excluded from the data analysis.

References for Supporting Information

1 Taken verbatim or adapted from: M. A. Morris, M. Malek, M. H. Hashemian, B. T. Nguyen, S. Manuse, K. Lewis and J. S. Nowick, *ACS Chem. Biol.*, 2020, **15**, 1222–1231.

2 H. Yang, K. H. Chen and J. S. Nowick, *ACS Chem. Biol.*, 2016, **11**, 1823–1826.

3 Taken verbatim or adapted from: K. H. Chen, S. P. Le, X. Han, J. M. Frias and J. S. Nowick, *Chem. Commun.*, 2017, **53**, 11357–11359.

4 Adapted from: B. C. Evans, C. E. Nelson, S. S. Yu, K. R. Beavers, A. J. Kim, H. Li, H. M. Nelson, T. D. Giorgio and C. L. Duvall, *J. Vis. Exp.*, 2013, 50166.

5 Adapted from: A. Oddo and P. R. Hansen, in *Antimicrobial Peptides: Methods and Protocols*, ed. P. R. Hansen, Springer, New York, NY, 2017, pp. 427–435.

6 Taken verbatim or adapted from: K. Sikora, D. Neubauer, M. Jaśkiewicz and W. Kamysz, *Int. J. Pept. Res. Ther.*, 2018, **24**, 265–270.

7 Taken verbatim or adapted from: L. L. Ling, T. Schneider, A. J. Peoples, A. L. Spoering, I. Engels, B. P. Conlon, A. Mueller, T. F. Schäberle, D. E. Hughes, S. Epstein, M. Jones, L. Lazarides, V. A. Steadman, D. R. Cohen, C. R. Felix, K. A. Fetterman, W. P. Millett, A. G. Nitti, A. M. Zullo, C. Chen and K. Lewis, *Nature*, 2015, **517**, 455–459.

8 Taken verbatim or adapted from procedure provided by NeoSome Life Sciences, LLC.

9 All plate layout figures were created using BioRender.com.

Characterization data

Lys$_{10}$-teixobactin Analytical HPLC Trace and MALDI-TOF

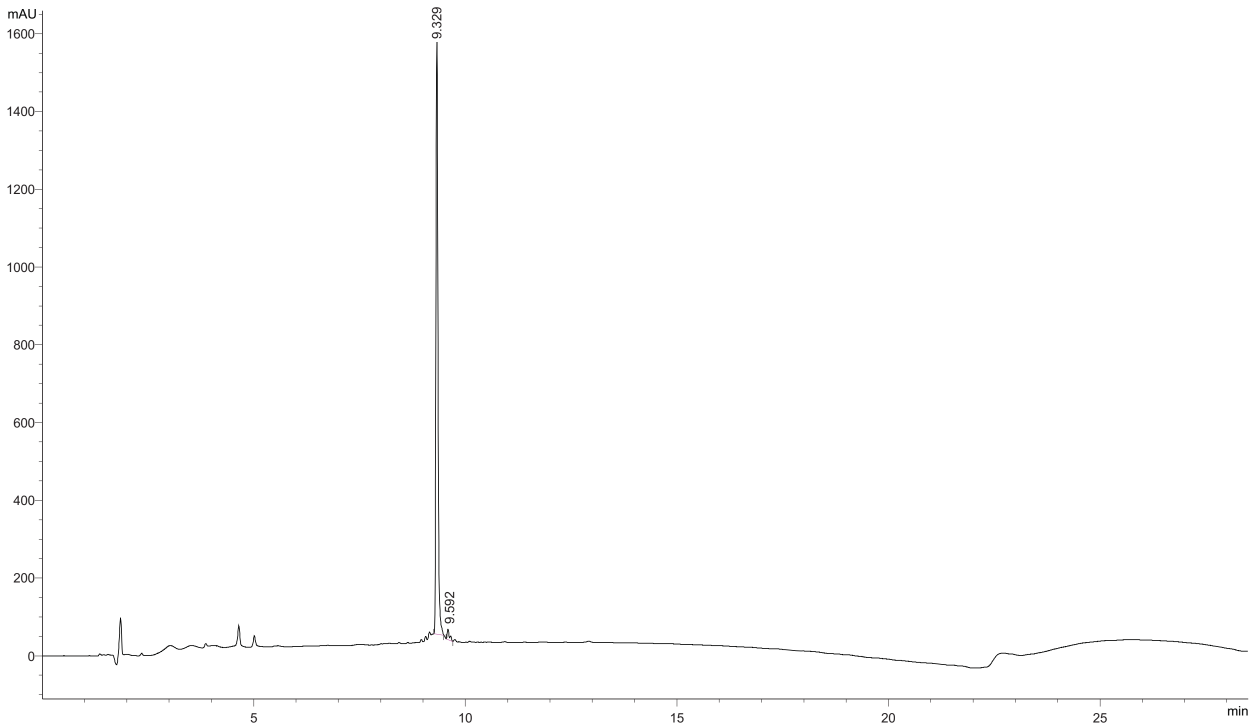

Peak #	RetTime [min]	Type	Width [min]	Area [mAU*s]	Height [mAU]	Area %
1	9.329	MM	0.0555	5090.91113	1529.24243	98.0622
2	9.592	MM	0.0627	100.60265	26.76234	1.9378

Totals : 5191.51379 1556.00477

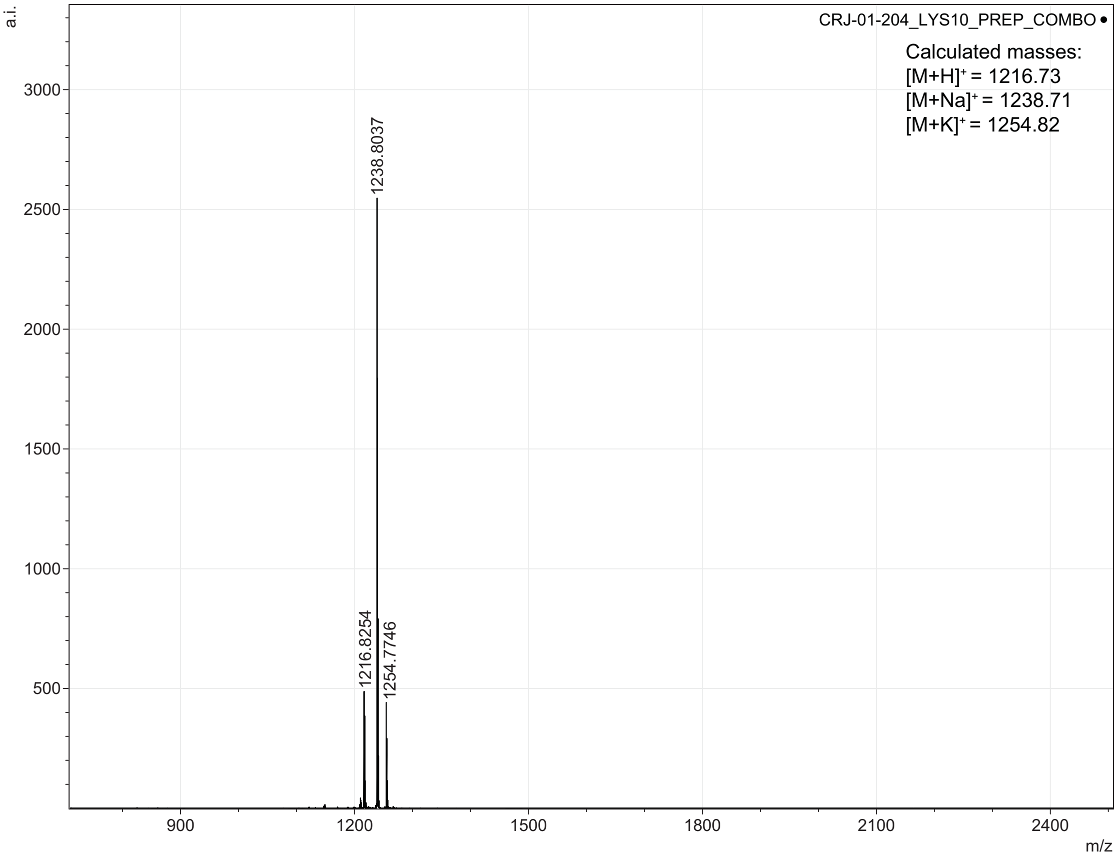

CRJ-01-204_LYS10_PREP_COMBO
Calculated masses:
[M+H]+ = 1216.73
[M+Na]+ = 1238.71
[M+K]+ = 1254.82
1238.8037
1216.8254
1254.7746
a.i.
m/z

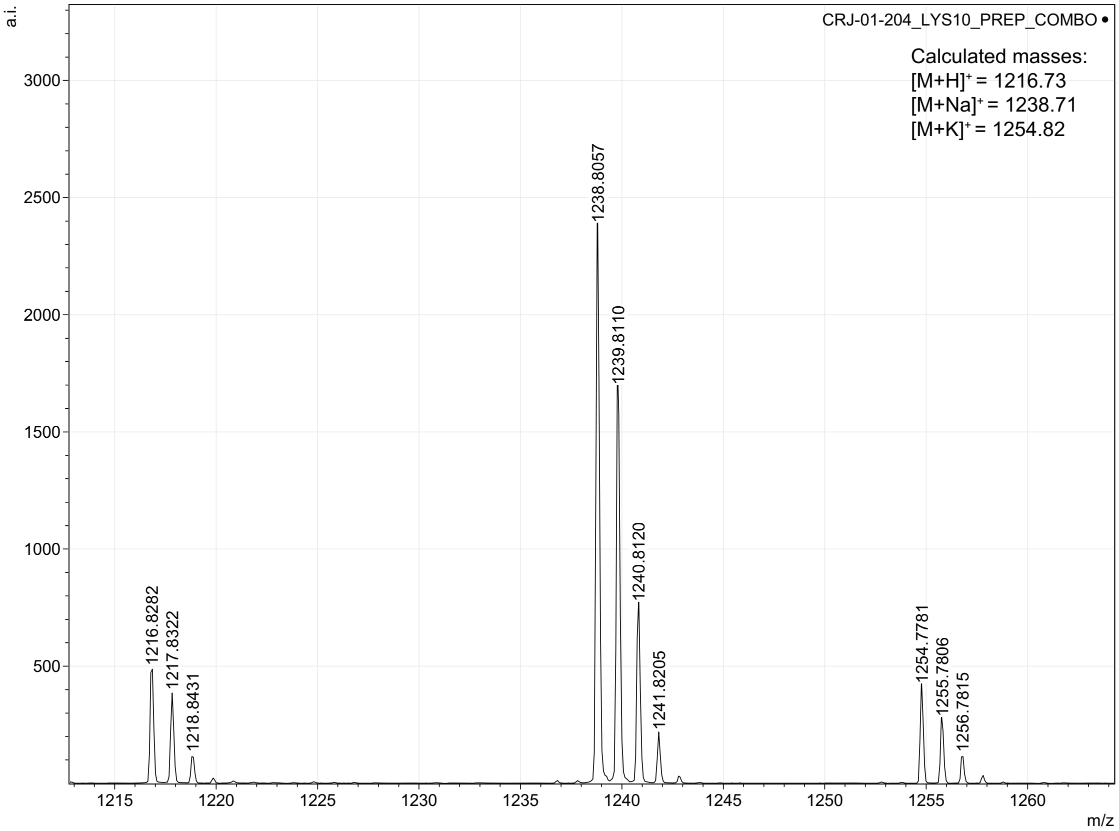

CRJ-01-204_LYS10_PREP_COMBO
Calculated masses:
[M+H]+ = 1216.73
[M+Na]+ = 1238.71
[M+K]+ = 1254.82
1216.8282
1217.8322
1218.8431
1238.8057
1239.8110
1240.8120
1241.8205
1254.7781
1255.7806
1256.7815
a.i.
m/z

Lys$_{10}$-teixobactin Prodrug A Analytical HPLC Trace and MALDI-TOF

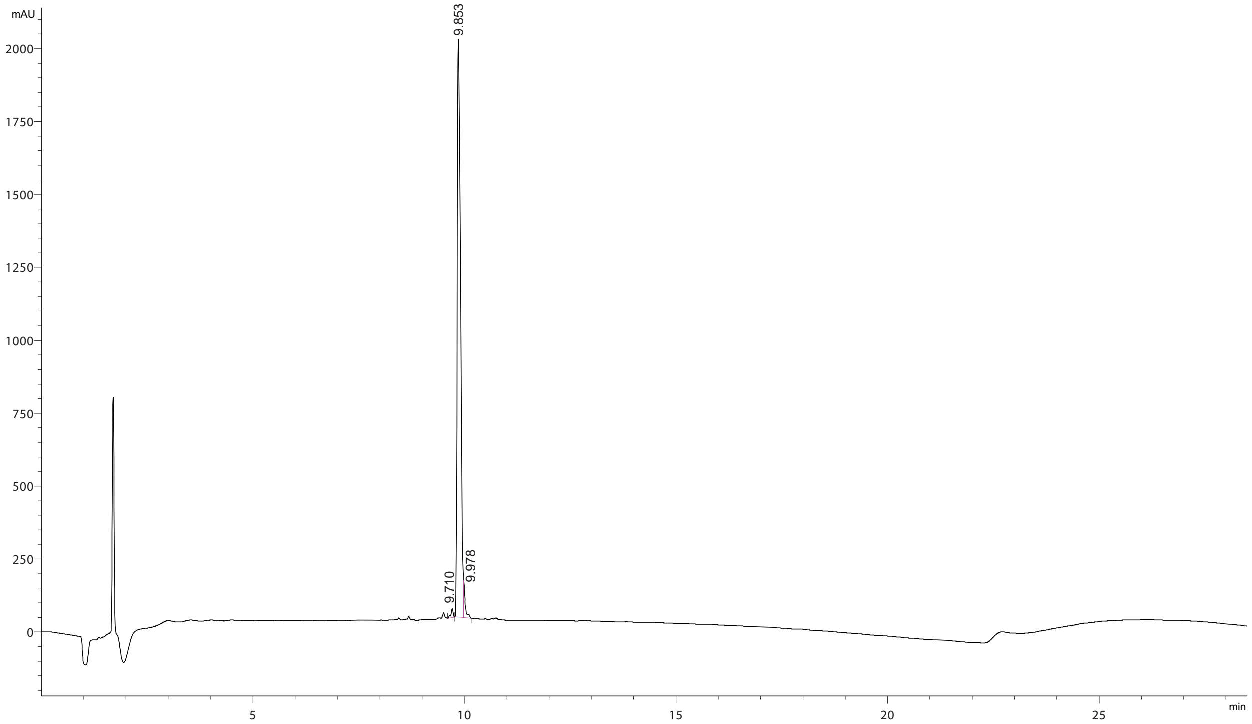

Peak #	RetTime [min]	Type	Width [min]	Area [mAU*s]	Height [mAU]	Area %
1	9.710	MM	0.0603	107.60345	29.74174	0.9415
2	9.853	MF	0.0924	1.10231e4	1988.32227	96.4500
3	9.978	FM	0.0382	298.12329	130.14198	2.6085

Totals : 1.14288e4 2148.20599

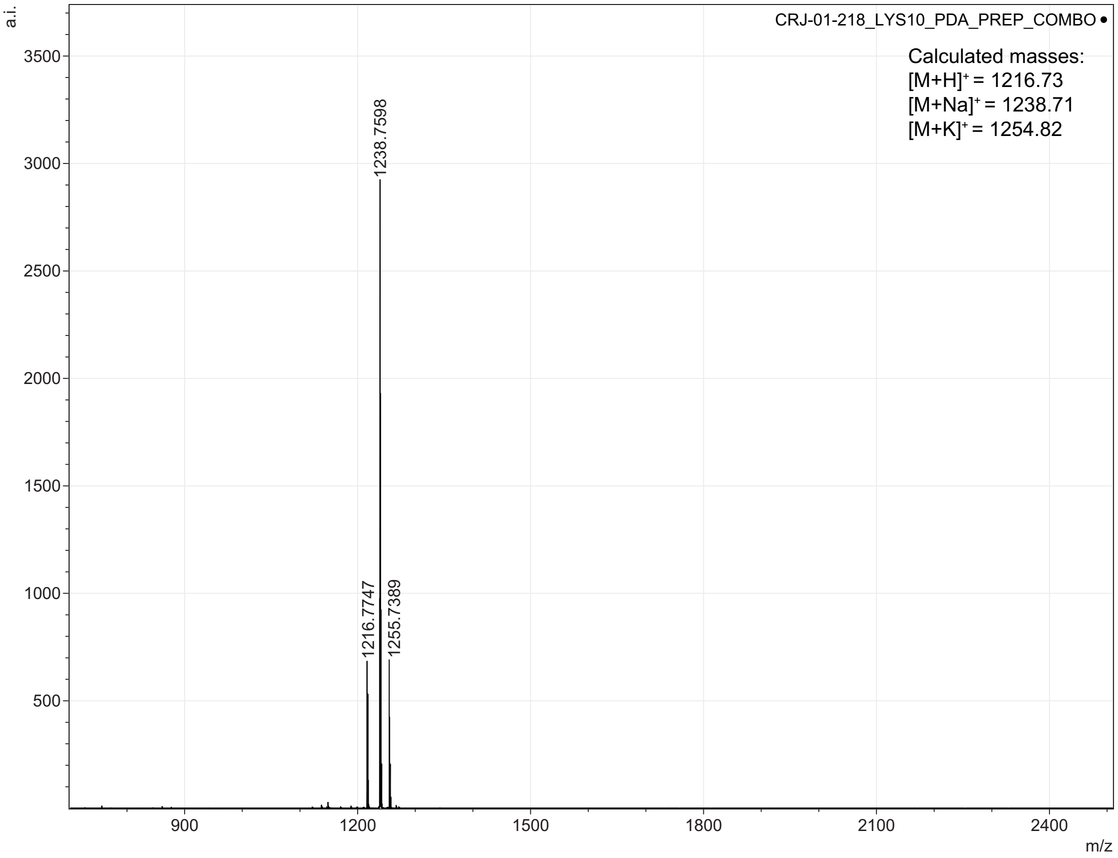

CRJ-01-218_LYS10_PDA_PREP_COMBO
Calculated masses:
[M+H]+ = 1216.73
[M+Na]+ = 1238.71
[M+K]+ = 1254.82
1238.7598
1216.7747
1255.7389
a.i.
m/z

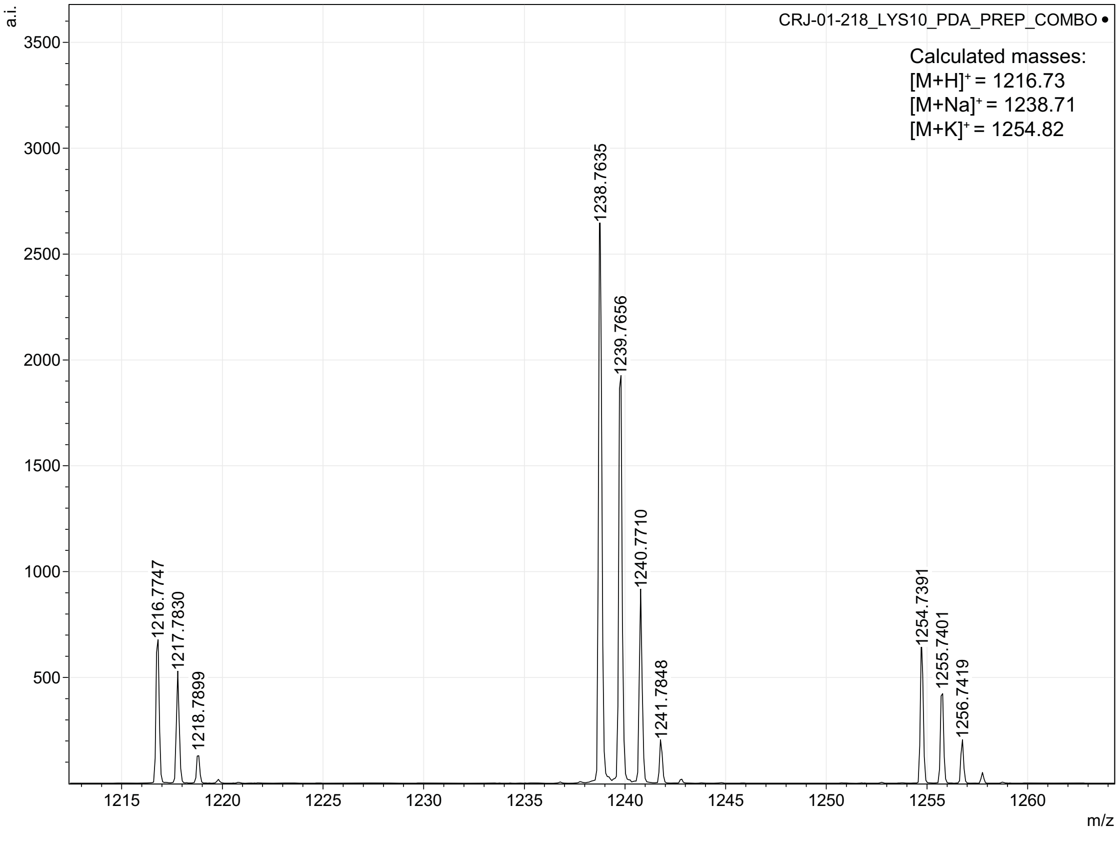

CRJ-01-218_LYS10_PDA_PREP_COMBO
Calculated masses:
[M+H]+ = 1216.73
[M+Na]+ = 1238.71
[M+K]+ = 1254.82
1238.7635
1239.7656
1240.7710
1241.7848
1216.7747
1217.7830
1218.7899
1254.7391
1255.7401
1256.7419
a.i.
m/z

Lys$_{10}$-teixobactin Prodrug B Analytical HPLC Trace and MALDI-TOF

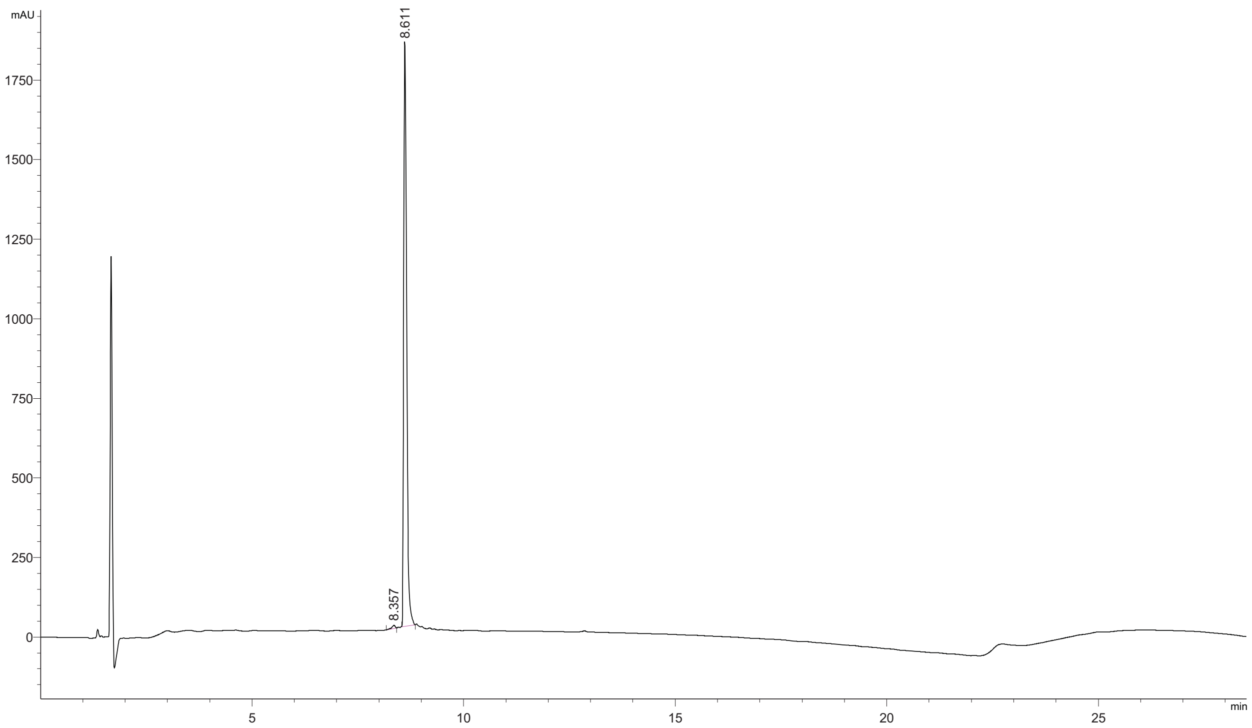

Peak #	RetTime [min]	Type	Width [min]	Area [mAU*s]	Height [mAU]	Area %
1	8.357	MM	0.1002	62.18367	10.33866	0.6828
2	8.611	MM	0.0815	9045.53809	1848.98840	99.3172

Totals : 9107.72176 1859.32707

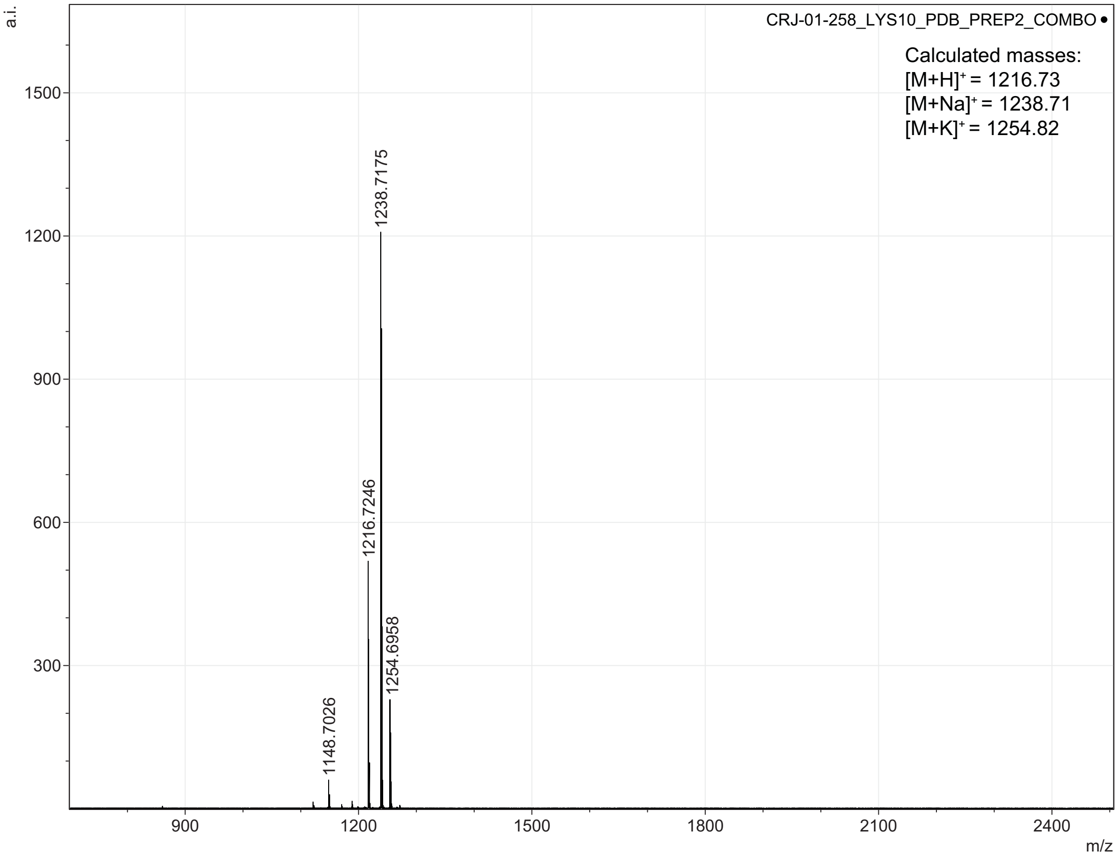
CRJ-01-258_LYS10_PDB_PREP2_COMBO
Calculated masses:
[M+H]+ = 1216.73
[M+Na]+ = 1238.71
[M+K]+ = 1254.82
a.i.
1500
1200
900
600
300
1238.7175
1216.7246
1254.6958
1148.7026
900
1200
1500
1800
2100
2400
m/z

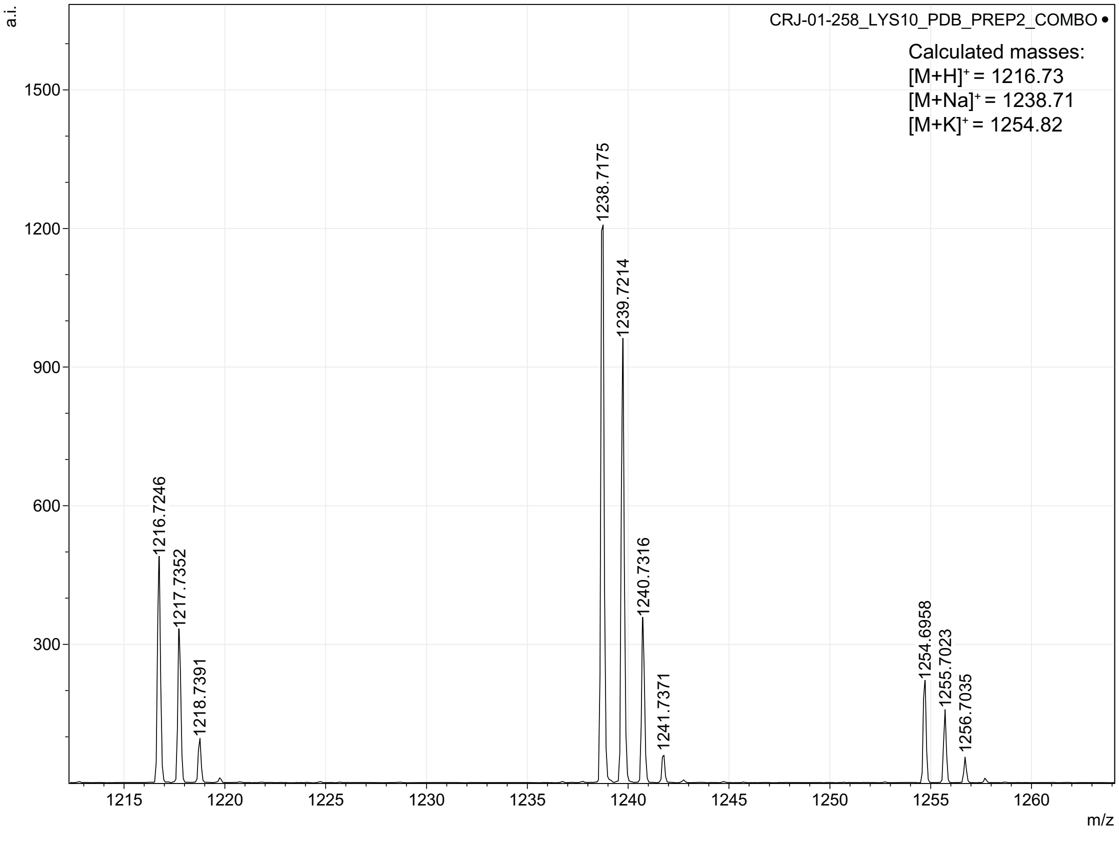
CRJ-01-258_LYS10_PDB_PREP2_COMBO
Calculated masses:
[M+H]+ = 1216.73
[M+Na]+ = 1238.71
[M+K]+ = 1254.82
a.i.
1500
1200
900
600
300
1238.7175
1239.7214
1216.7246
1217.7352
1240.7316
1218.7391
1241.7371
1254.6958
1255.7023
1256.7035
1215
1220
1225
1230
1235
1240
1245
1250
1255
1260
m/z

Lys$_{10}$-teixobactin Prodrug C Analytical HPLC Trace and MALDI-TOF

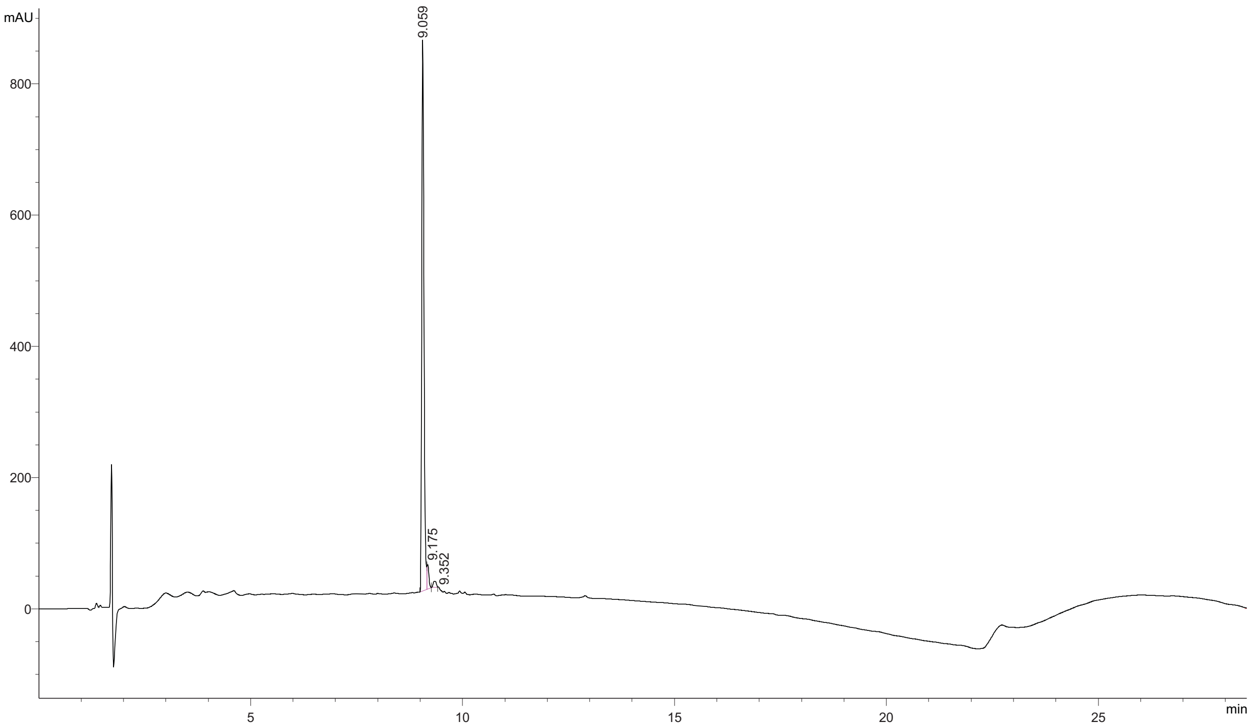

Peak #	RetTime [min]	Type	Width [min]	Area [mAU*s]	Height [mAU]	Area %
1	9.059	MF	0.0602	3045.64526	843.11633	95.0579
2	9.175	FM	0.0479	108.82409	37.82890	3.3965
3	9.352	MM	0.0870	49.51868	9.48839	1.5455

Totals : 3203.98803 890.43363

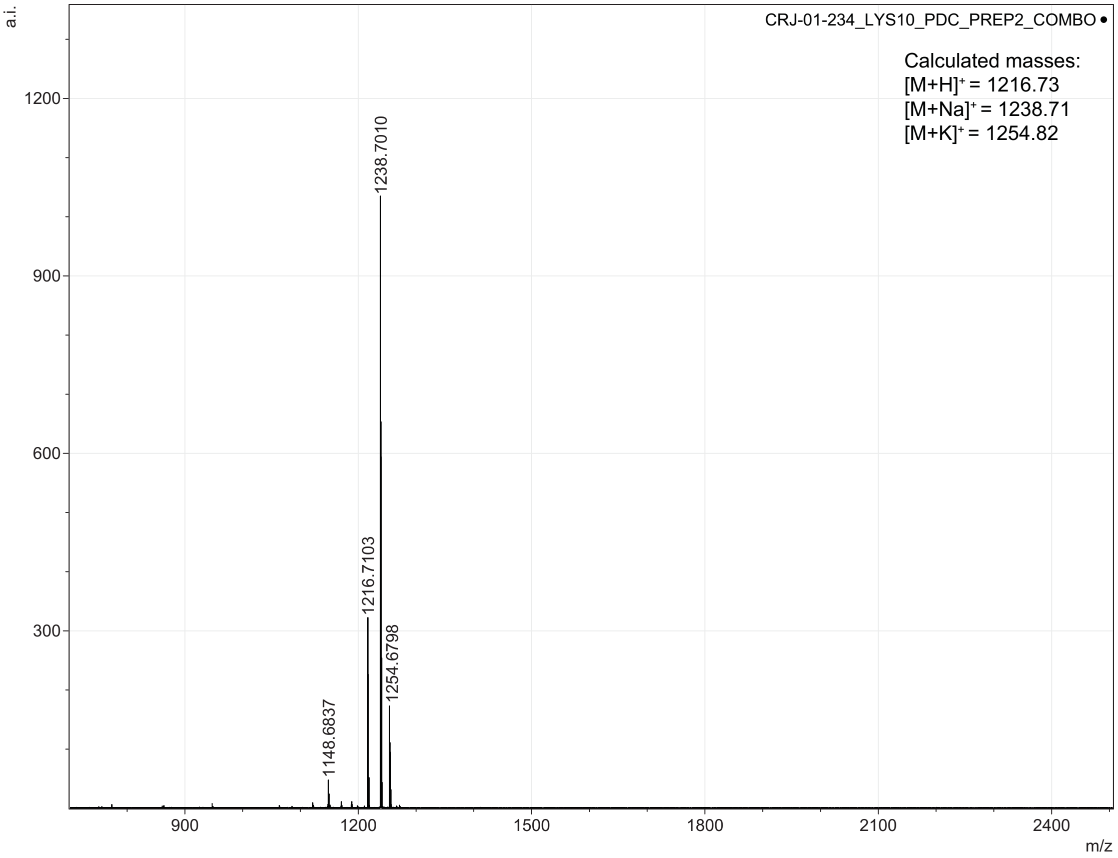

CRJ-01-234_LYS10_PDC_PREP2_COMBO
Calculated masses:
[M+H]+ = 1216.73
[M+Na]+ = 1238.71
[M+K]+ = 1254.82
a.i.
1200
900
600
300
1238.7010
1216.7103
1254.6798
1148.6837
900
1200
1500
1800
2100
2400
m/z

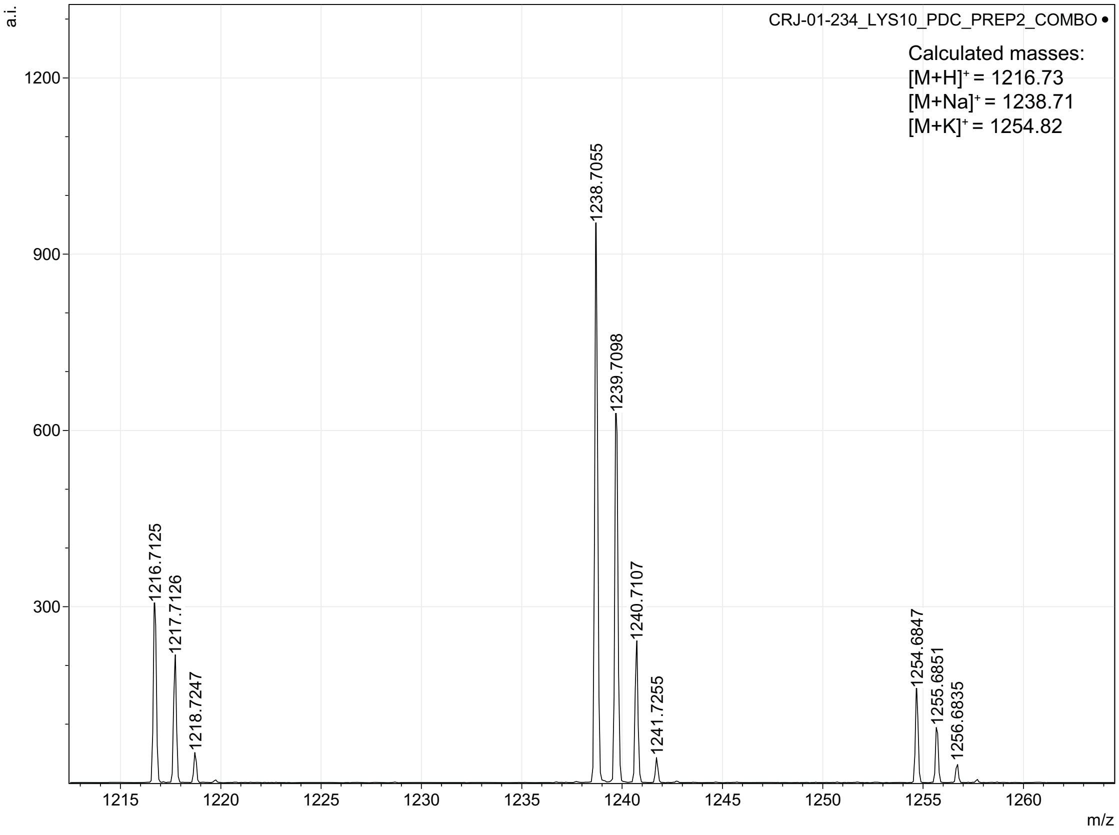

CRJ-01-234_LYS10_PDC_PREP2_COMBO
Calculated masses:
[M+H]+ = 1216.73
[M+Na]+ = 1238.71
[M+K]+ = 1254.82
a.i.
1200
900
600
300
1216.7125
1217.7126
1218.7247
1238.7055
1239.7098
1240.7107
1241.7255
1254.6847
1255.6851
1256.6835
1215
1220
1225
1230
1235
1240
1245
1250
1255
1260
m/z

Arg$_{10}$-teixobactin Analytical HPLC Trace and MALDI-TOF

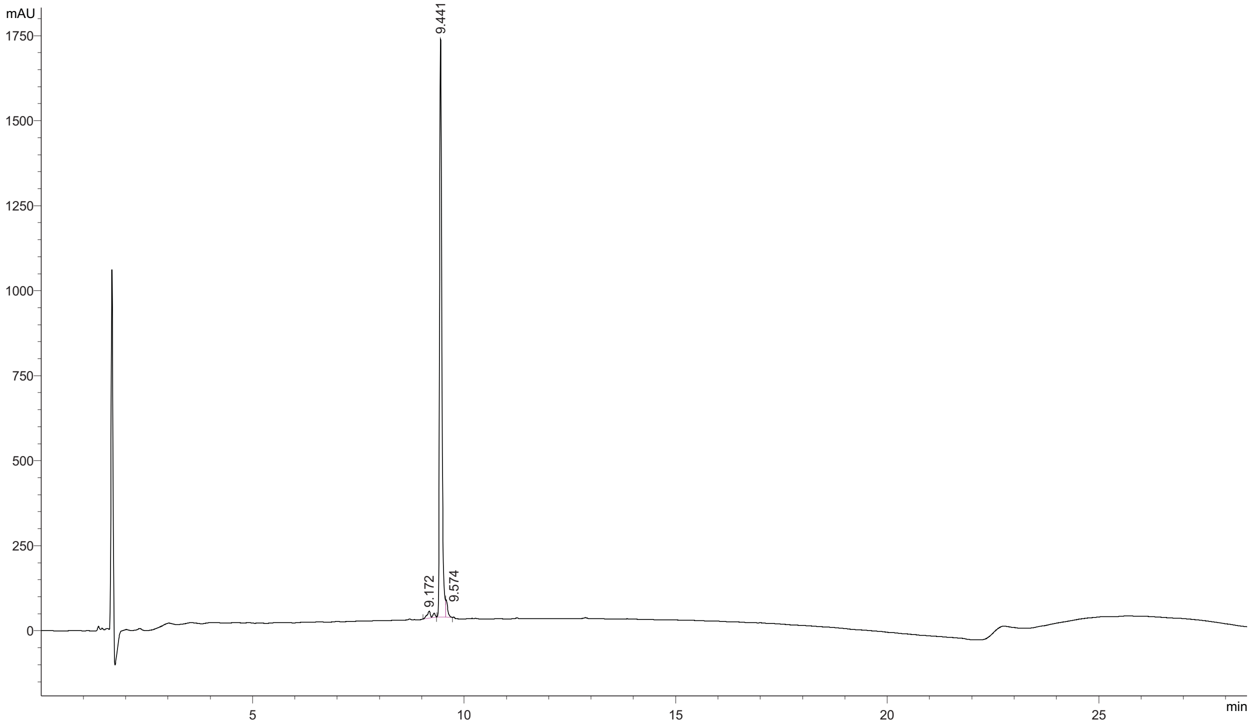

Peak #	RetTime [min]	Type	Width [min]	Area [mAU*s]	Height [mAU]	Area %
1	9.172	MM	0.1281	162.81050	21.17930	2.4426
2	9.441	MF	0.0614	6344.79980	1722.18896	95.1874
3	9.574	FM	0.0517	157.97453	50.89952	2.3700
Totals :				6665.58484	1794.26778	

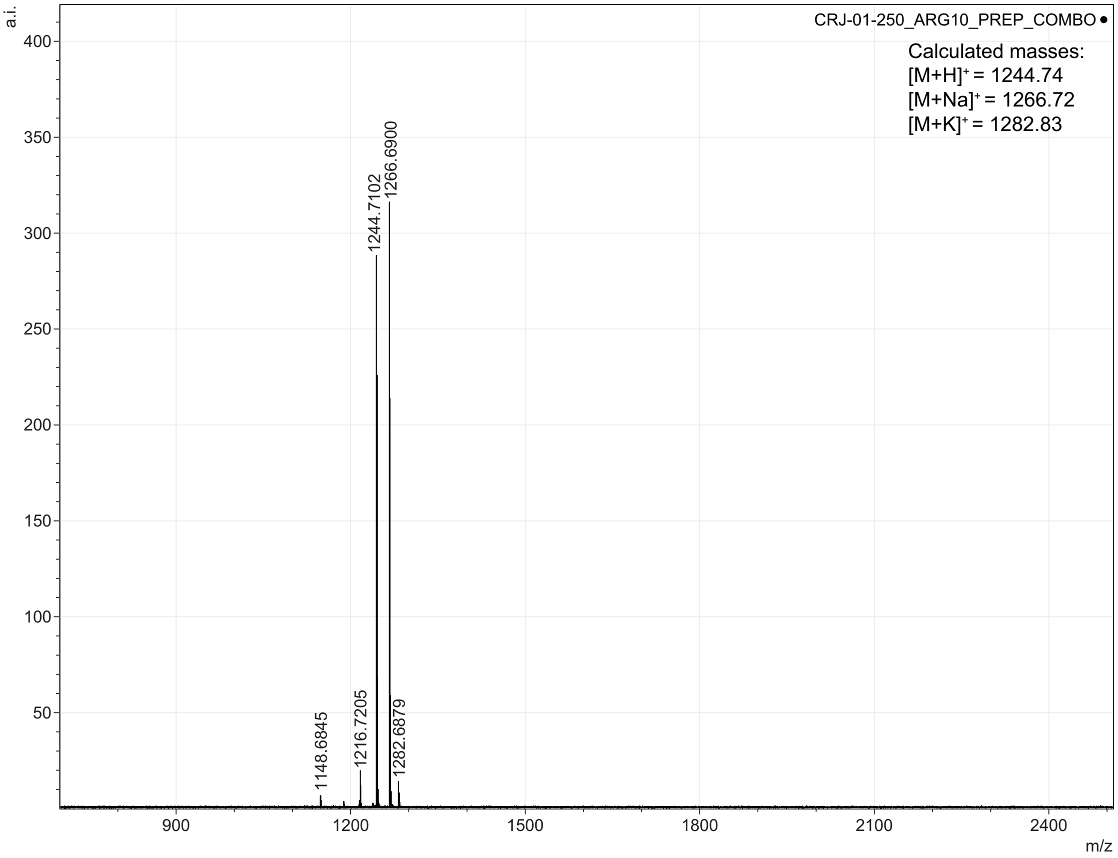
CRJ-01-250_ARG10_PREP_COMBO
Calculated masses:
[M+H]+ = 1244.74
[M+Na]+ = 1266.72
[M+K]+ = 1282.83
a.i.
m/z
1148.6845
1216.7205
1244.7102
1266.6900
1282.6879

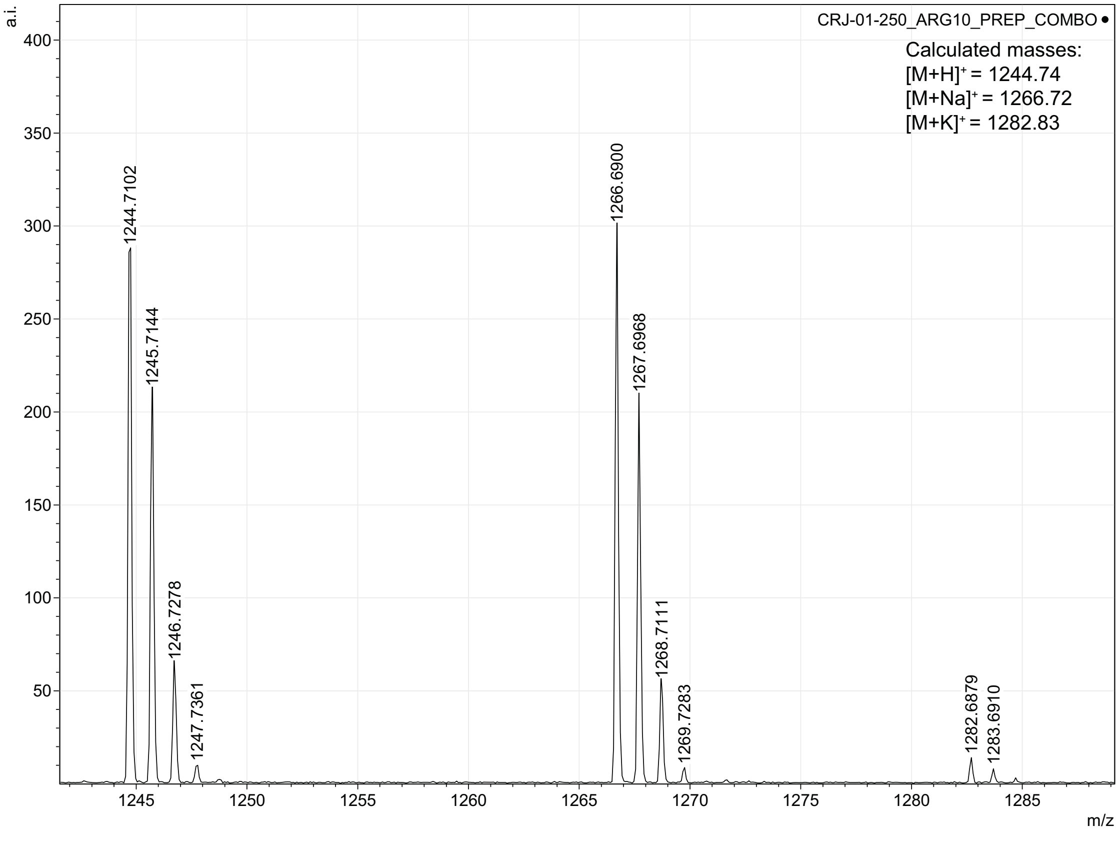
CRJ-01-250_ARG10_PREP_COMBO
Calculated masses:
[M+H]+ = 1244.74
[M+Na]+ = 1266.72
[M+K]+ = 1282.83
a.i.
m/z
1244.7102
1245.7144
1246.7278
1247.7361
1266.6900
1267.6968
1268.7111
1269.7283
1282.6879
1283.6910

Arg$_{10}$-teixobactin Prodrug A Analytical HPLC Trace and MALDI-TOF

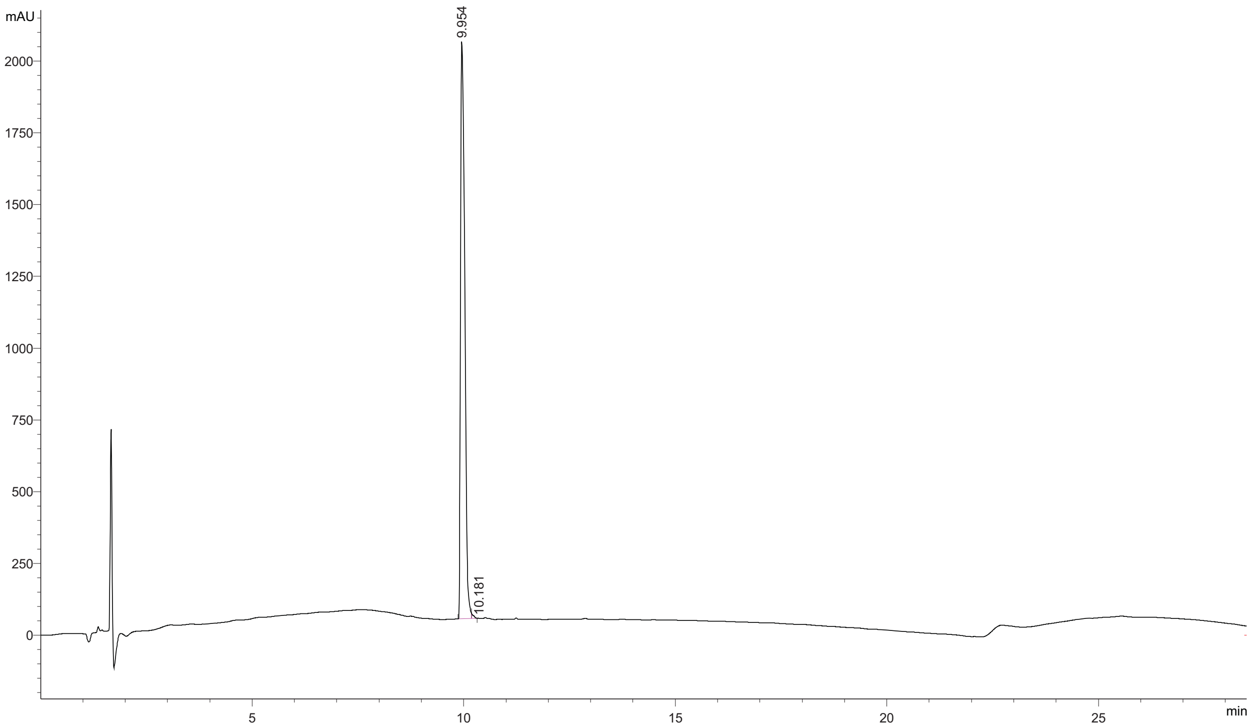

Peak #	RetTime [min]	Type	Width [min]	Area [mAU*s]	Height [mAU]	Area %
1	9.954	MF	0.1167	1.41014e4	2013.23596	99.6042
2	10.181	FM	0.0521	56.02947	17.92281	0.3958

Totals : 1.41574e4 2031.15877

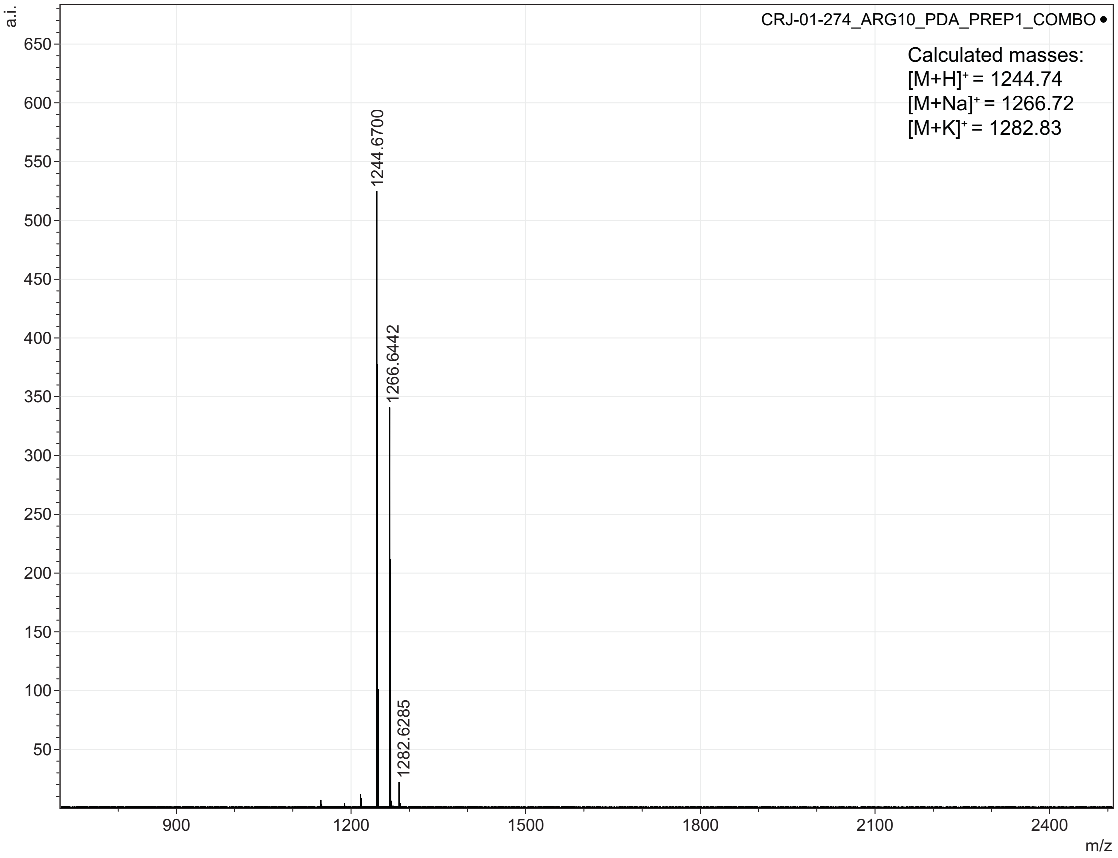
CRJ-01-274_ARG10_PDA_PREP1_COMBO
Calculated masses:
[M+H]+ = 1244.74
[M+Na]+ = 1266.72
[M+K]+ = 1282.83
1244.6700
1266.6442
1282.6285
a.i.
m/z

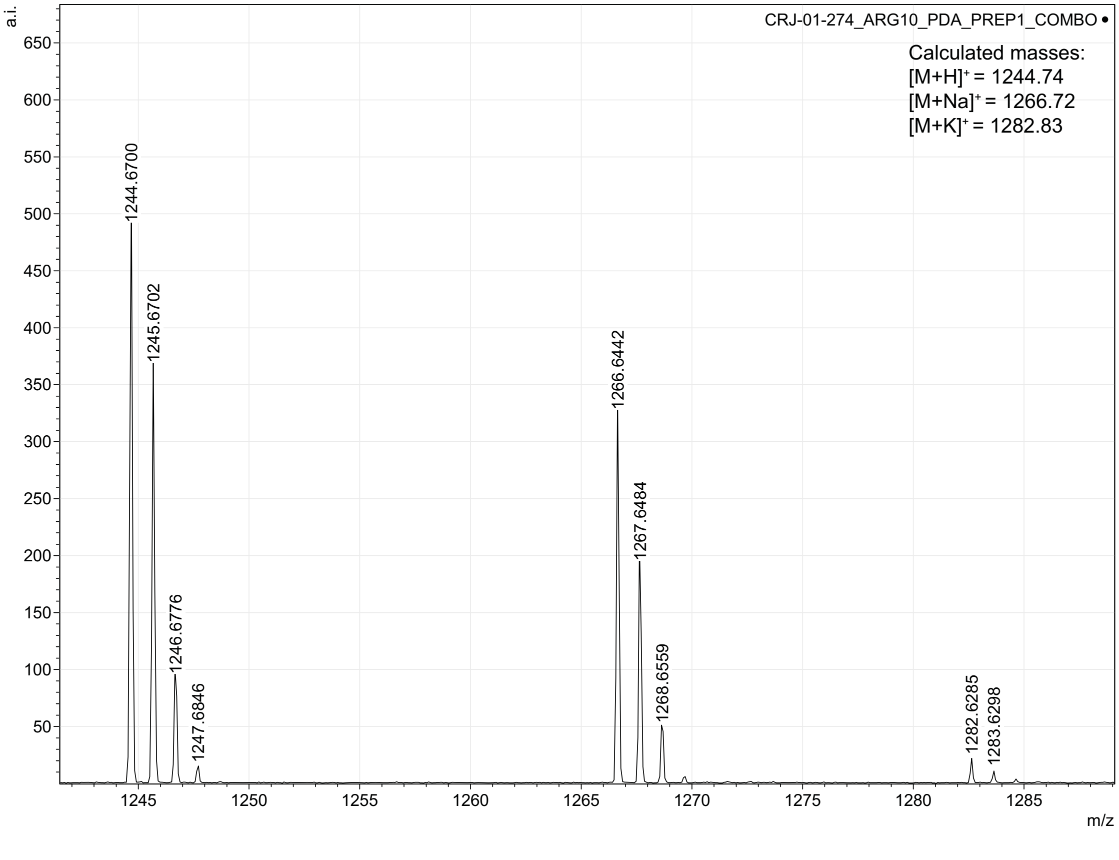
CRJ-01-274_ARG10_PDA_PREP1_COMBO
Calculated masses:
[M+H]+ = 1244.74
[M+Na]+ = 1266.72
[M+K]+ = 1282.83
1244.6700
1245.6702
1246.6776
1247.6846
1266.6442
1267.6484
1268.6559
1282.6285
1283.6298
a.i.
m/z

Arg$_{10}$-teixobactin Prodrug B Analytical HPLC Trace and MALDI-TOF

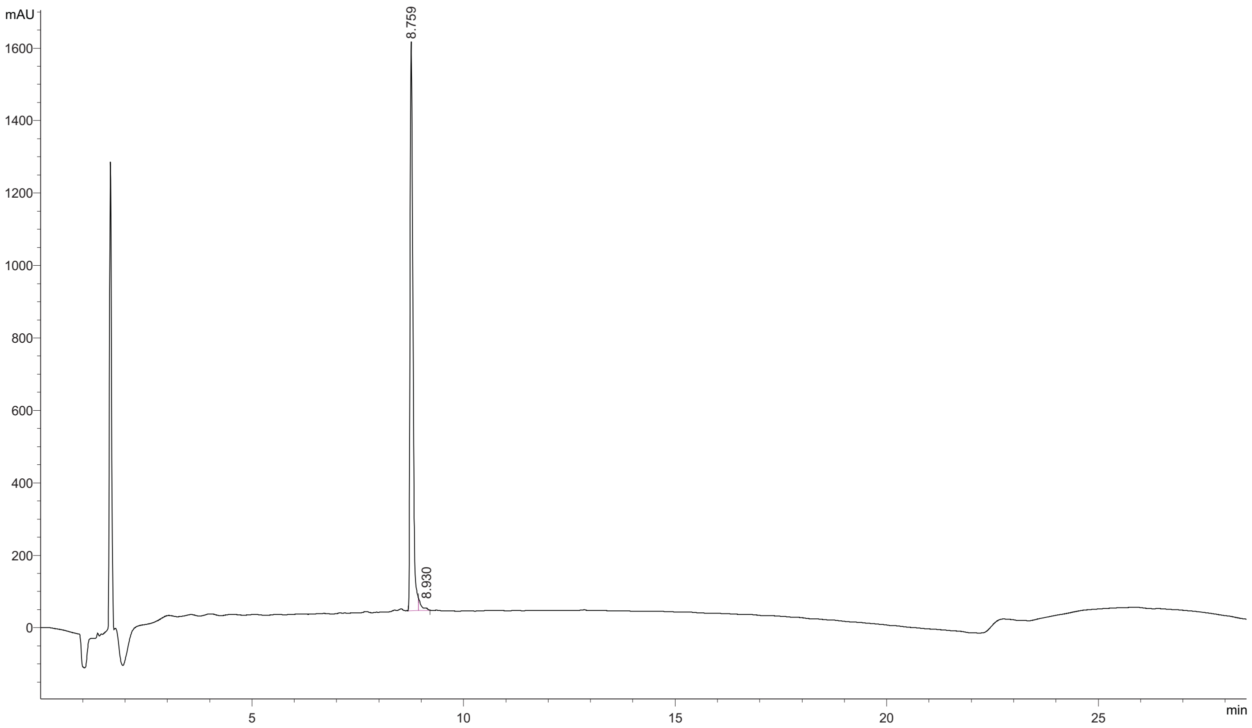

Peak #	RetTime [min]	Type	Width [min]	Area [mAU*s]	Height [mAU]	Area %
1	8.759	MF	0.0775	7334.88721	1576.97510	97.7861
2	8.930	FM	0.0803	166.06308	34.45243	2.2139

Totals : 7500.95029 1611.42752

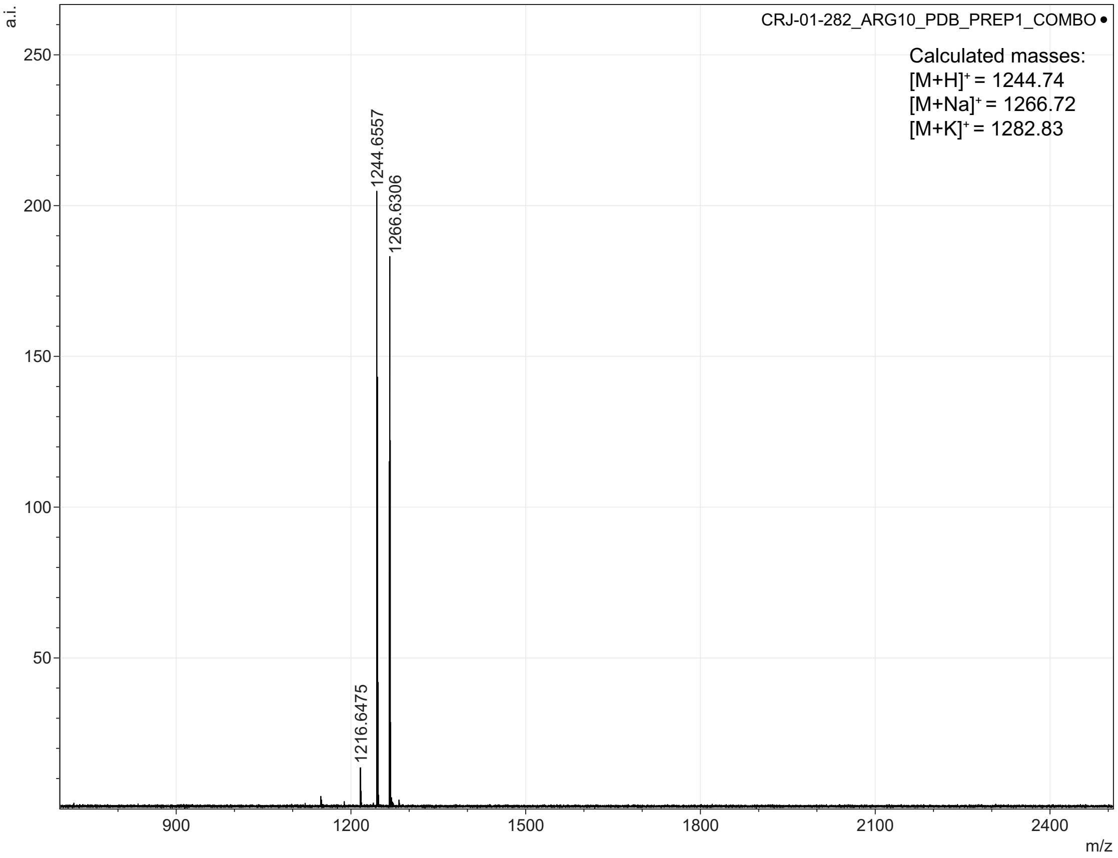

a.i.
CRJ-01-282_ARG10_PDB_PREP1_COMBO
Calculated masses:
[M+H]+ = 1244.74
[M+Na]+ = 1266.72
[M+K]+ = 1282.83
1244.6557
1266.6306
1216.6475
m/z

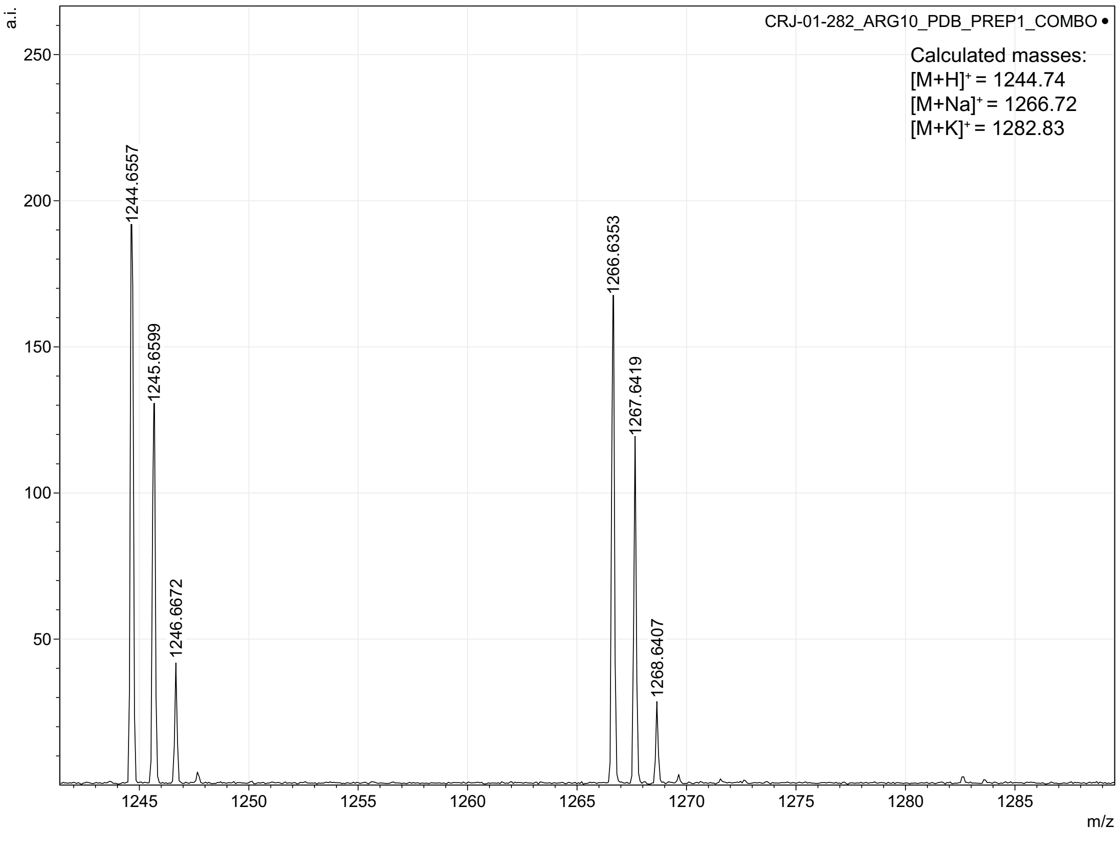

a.i.
CRJ-01-282_ARG10_PDB_PREP1_COMBO
Calculated masses:
[M+H]+ = 1244.74
[M+Na]+ = 1266.72
[M+K]+ = 1282.83
1244.6557
1245.6599
1246.6672
1266.6353
1267.6419
1268.6407
m/z

Arg$_{10}$-teixobactin Prodrug C Analytical HPLC Trace and MALDI-TOF

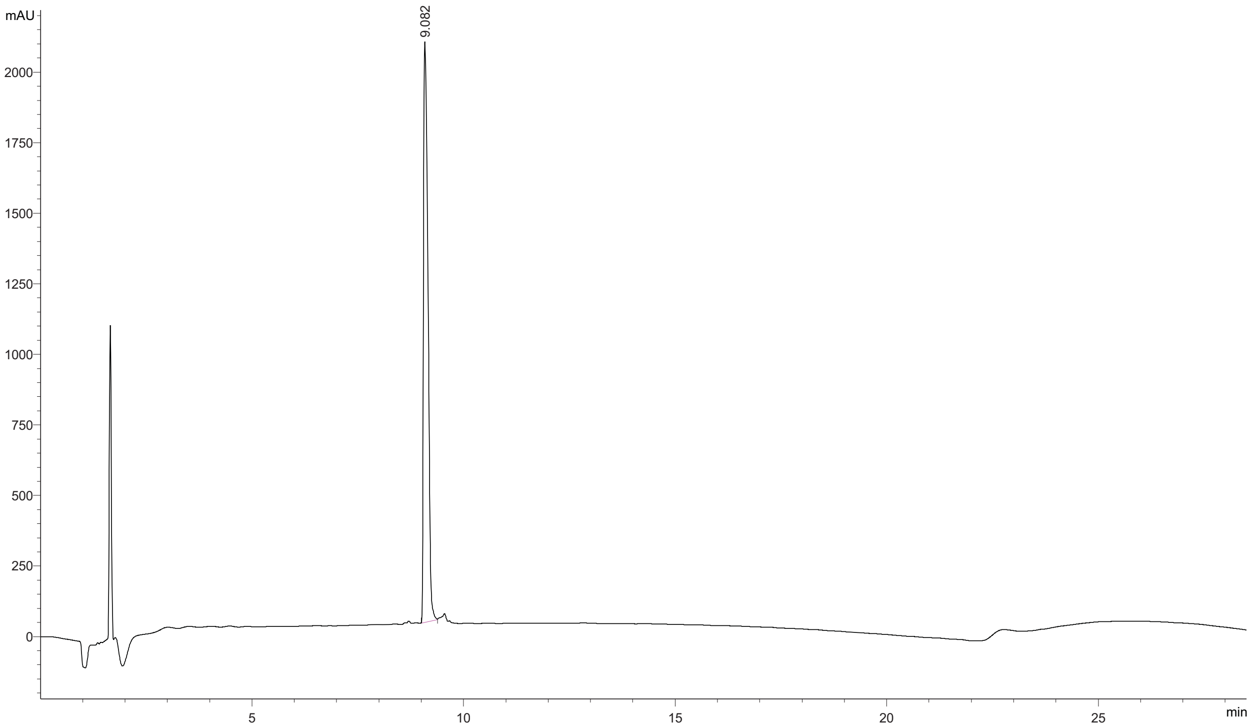

Peak #	RetTime [min]	Type	Width [min]	Area [mAU*s]	Height [mAU]	Area %
1	9.082	MM	0.1211	1.49948e4	2064.42993	100.0000
Totals :				1.49948e4	2064.42993	

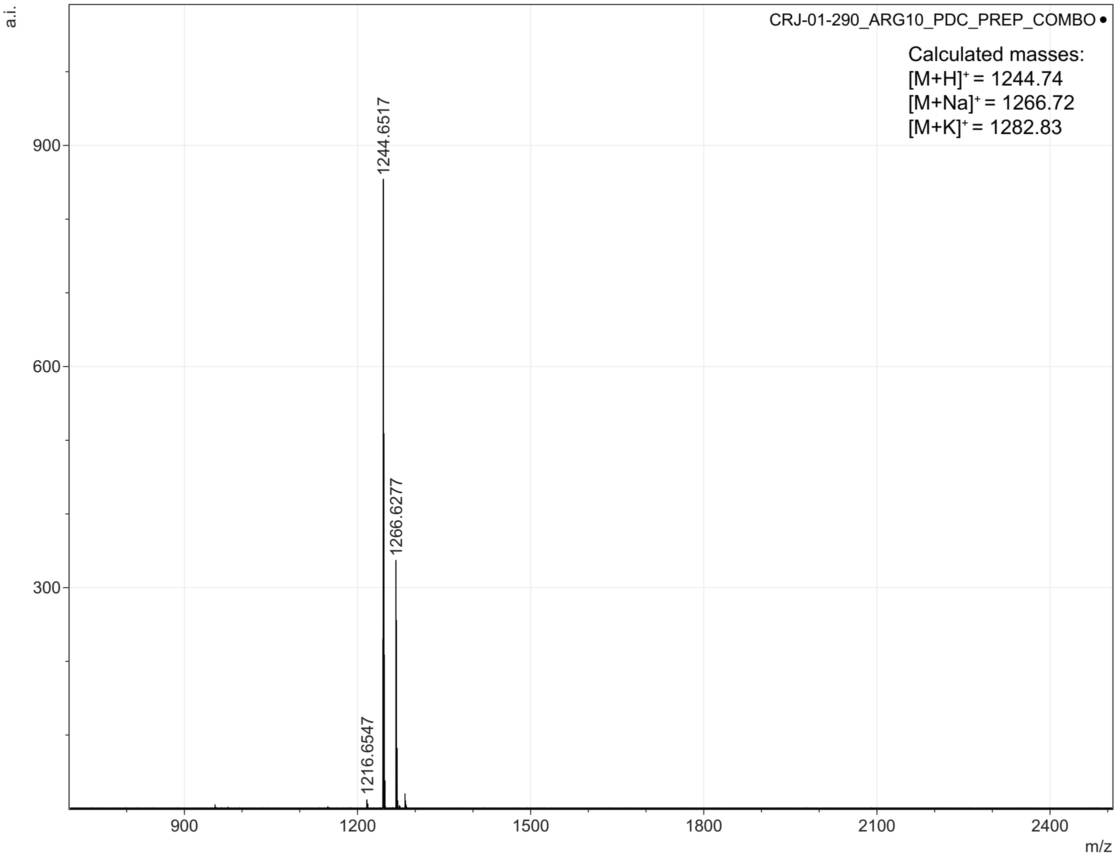
CRJ-01-290_ARG10_PDC_PREP_COMBO
Calculated masses:
[M+H]+ = 1244.74
[M+Na]+ = 1266.72
[M+K]+ = 1282.83
a.i.
m/z
1244.6517
1266.6277
1216.6547

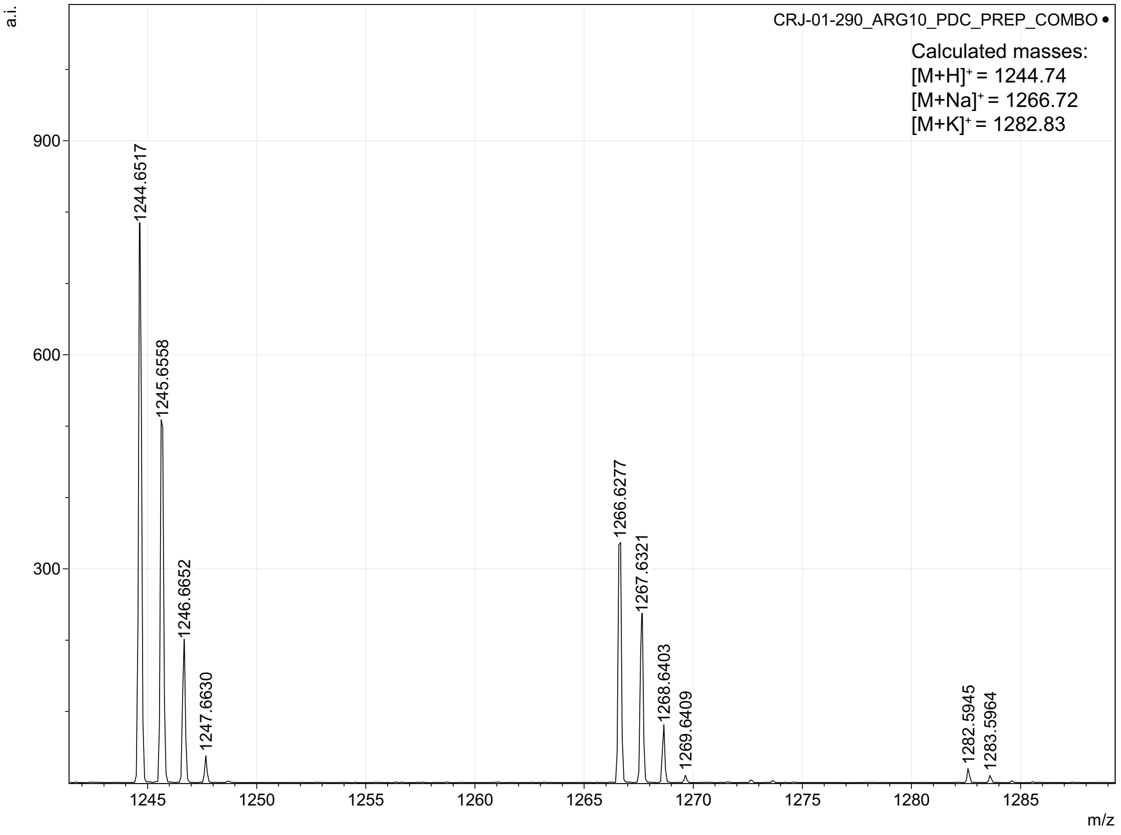
CRJ-01-290_ARG10_PDC_PREP_COMBO
Calculated masses:
[M+H]+ = 1244.74
[M+Na]+ = 1266.72
[M+K]+ = 1282.83
a.i.
m/z
1244.6517
1245.6558
1246.6652
1247.6630
1266.6277
1267.6321
1268.6403
1269.6409
1282.5945
1283.5964

Leu$_{10}$-teixobactin Analytical HPLC Trace and MALDI-TOF

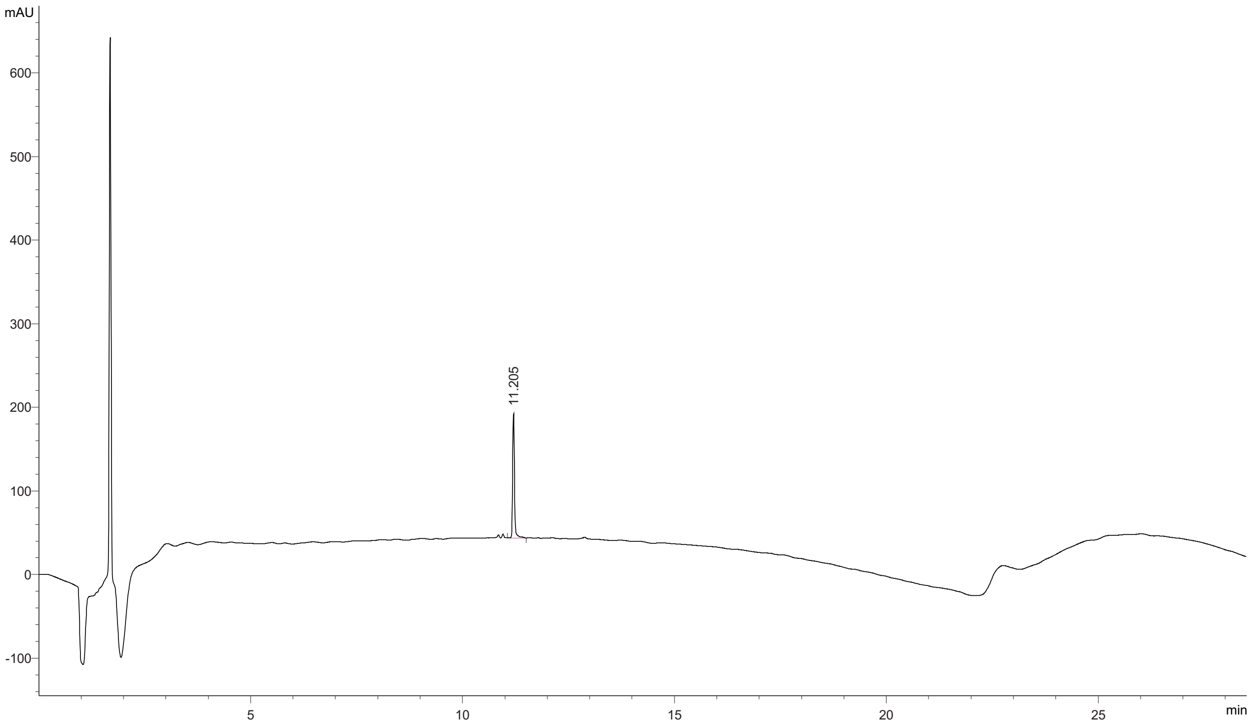

Peak #	RetTime [min]	Type	Width [min]	Area [mAU*s]	Height [mAU]	Area %
1	11.205	VV R	0.0475	456.96954	150.00790	100.0000
Totals :				456.96954	150.00790	

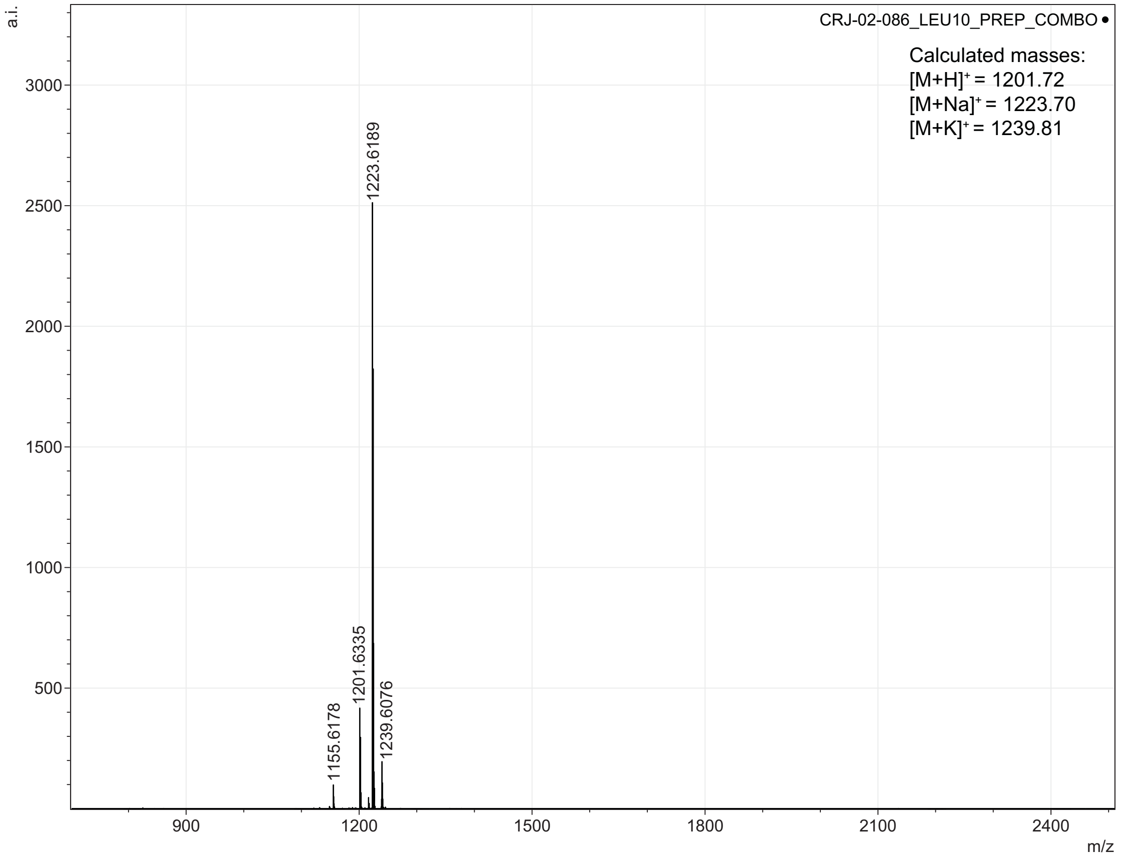
CRJ-02-086_LEU10_PREP_COMBO
Calculated masses:
[M+H]+ = 1201.72
[M+Na]+ = 1223.70
[M+K]+ = 1239.81
1223.6189
1201.6335
1239.6076
1155.6178
a.i.
m/z

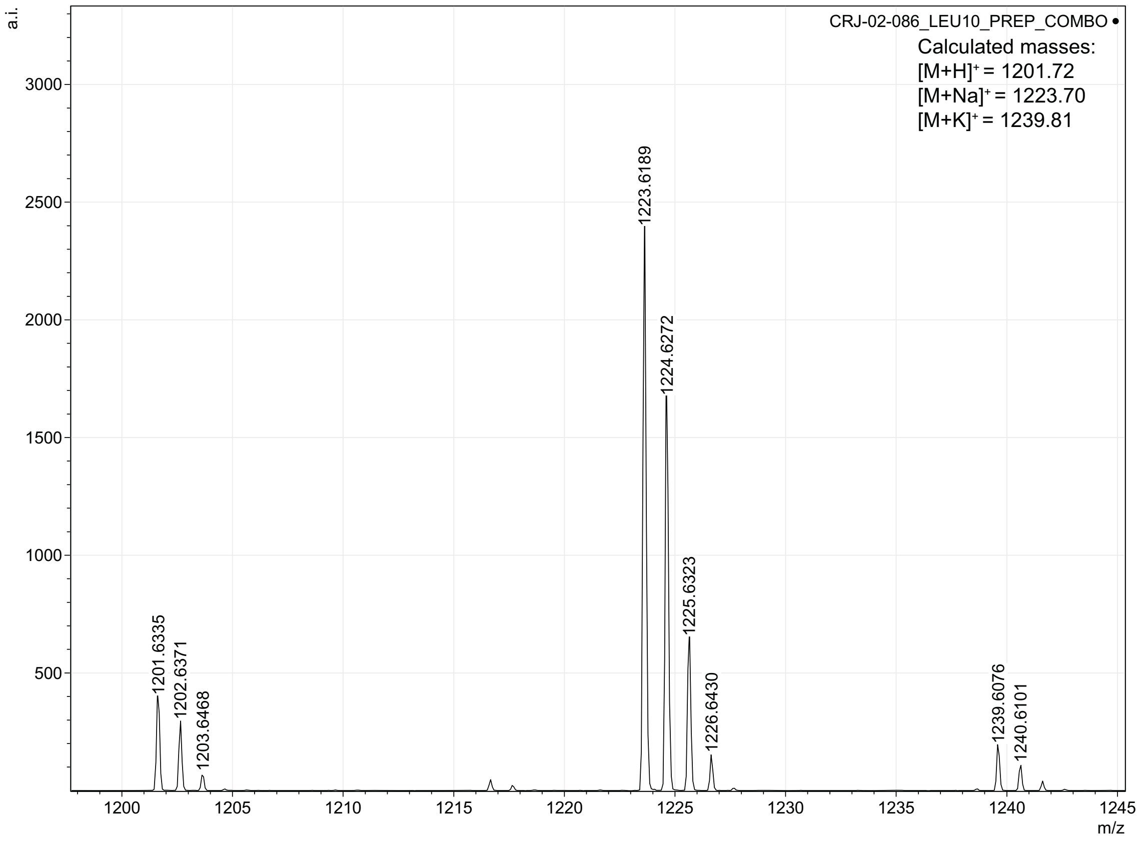
CRJ-02-086_LEU10_PREP_COMBO
Calculated masses:
[M+H]+ = 1201.72
[M+Na]+ = 1223.70
[M+K]+ = 1239.81
1223.6189
1224.6272
1225.6323
1226.6430
1201.6335
1202.6371
1203.6468
1239.6076
1240.6101
a.i.
m/z

Leu$_{10}$-teixobactin Prodrug A Analytical HPLC Trace and MALDI-TOF

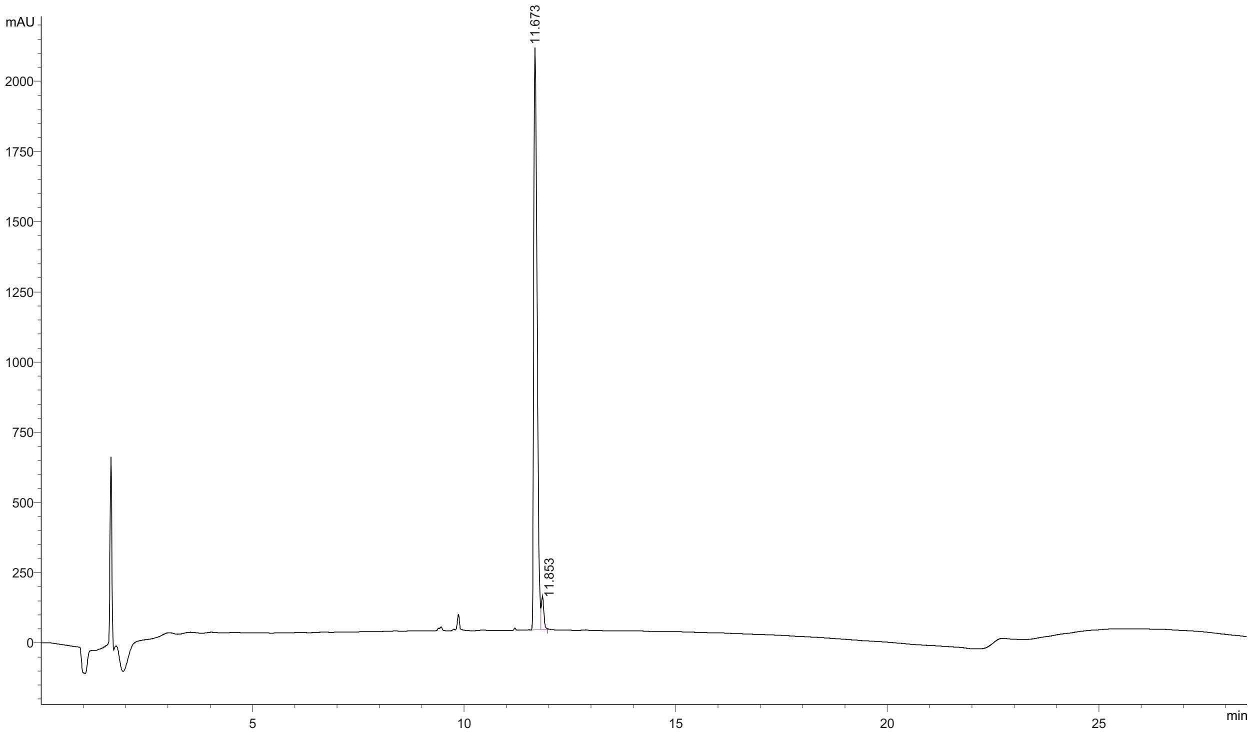

Peak #	RetTime [min]	Type	Width [min]	Area [mAU*s]	Height [mAU]	Area %
1	11.673	MF	0.0931	1.15965e4	2076.78662	96.1550
2	11.853	FM	0.0652	463.71695	118.46477	3.8450

Totals : 1.20603e4 2195.25139

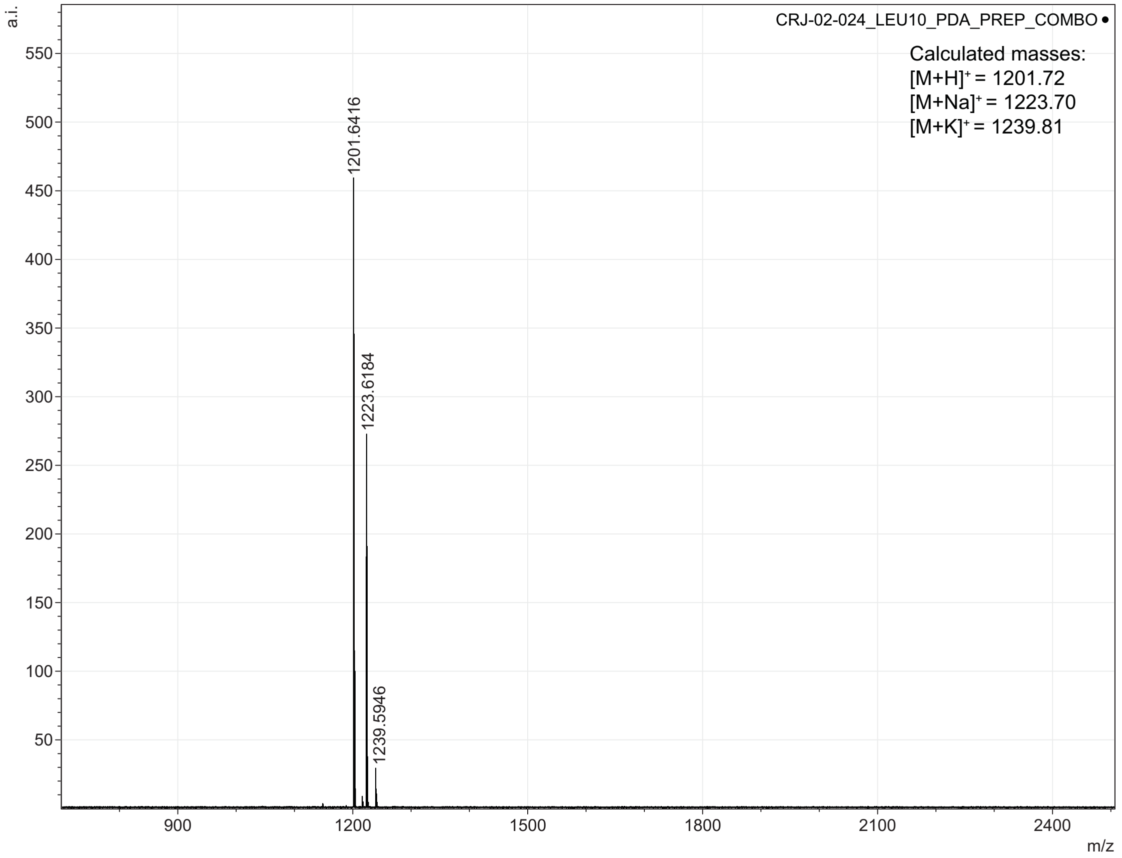
CRJ-02-024_LEU10_PDA_PREP_COMBO
Calculated masses:
[M+H]+ = 1201.72
[M+Na]+ = 1223.70
[M+K]+ = 1239.81
1201.6416
1223.6184
1239.5946
a.i.
m/z

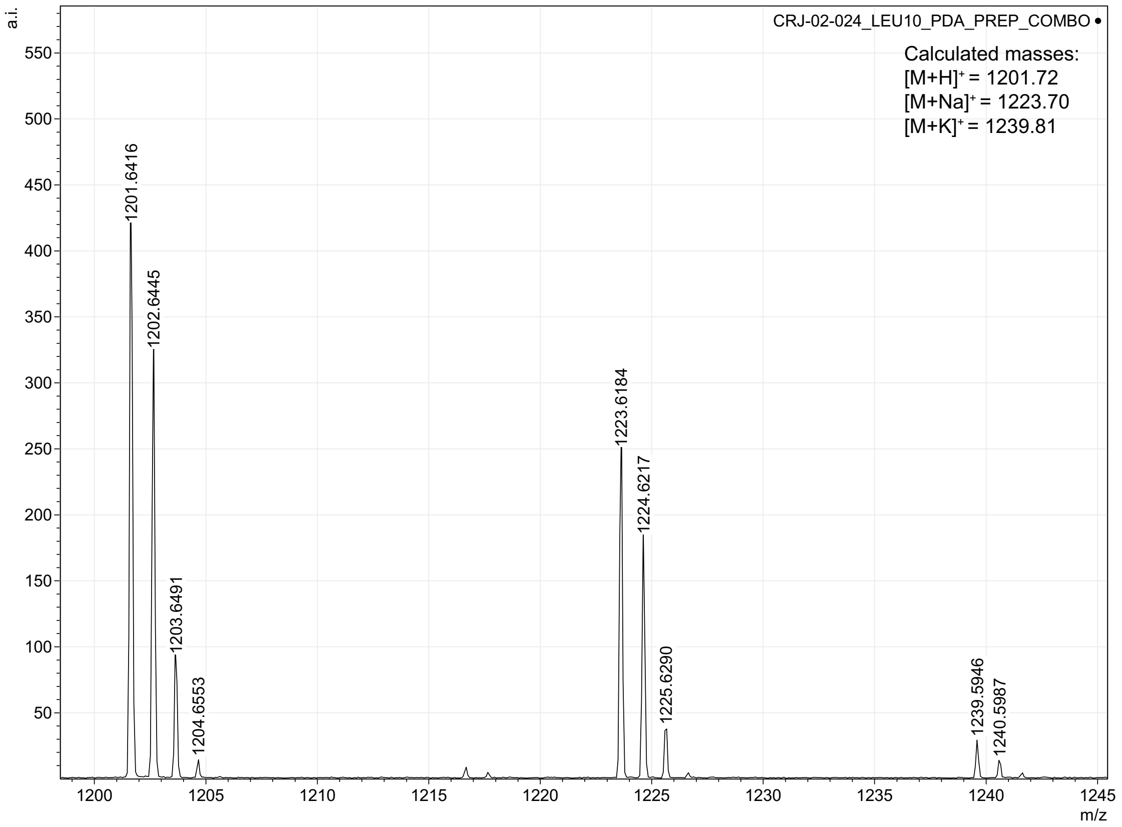
CRJ-02-024_LEU10_PDA_PREP_COMBO
Calculated masses:
[M+H]+ = 1201.72
[M+Na]+ = 1223.70
[M+K]+ = 1239.81
1201.6416
1202.6445
1203.6491
1204.6553
1223.6184
1224.6217
1225.6290
1239.5946
1240.5987
a.i.
m/z

Leu$_{10}$-teixobactin Prodrug B Analytical HPLC Trace and MALDI-TOF

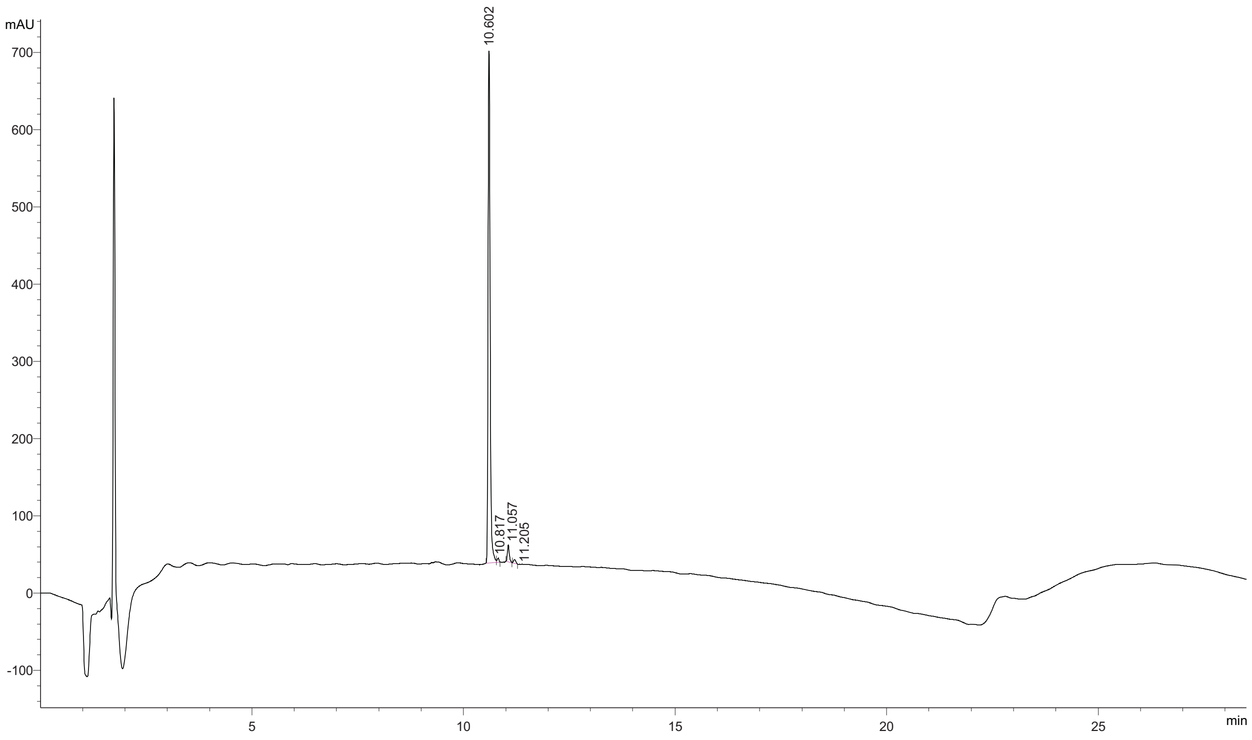

Peak #	RetTime [min]	Type	Width [min]	Area [mAU*s]	Height [mAU]	Area %
1	10.602	MF	0.0548	2208.07813	671.77911	95.6761
2	10.817	FM	0.0498	16.49998	5.52031	0.7149
3	11.057	MM	0.0499	65.42590	21.83677	2.8349
4	11.205	MM	0.0614	17.86347	4.85005	0.7740

Totals : 2307.86747 703.98624

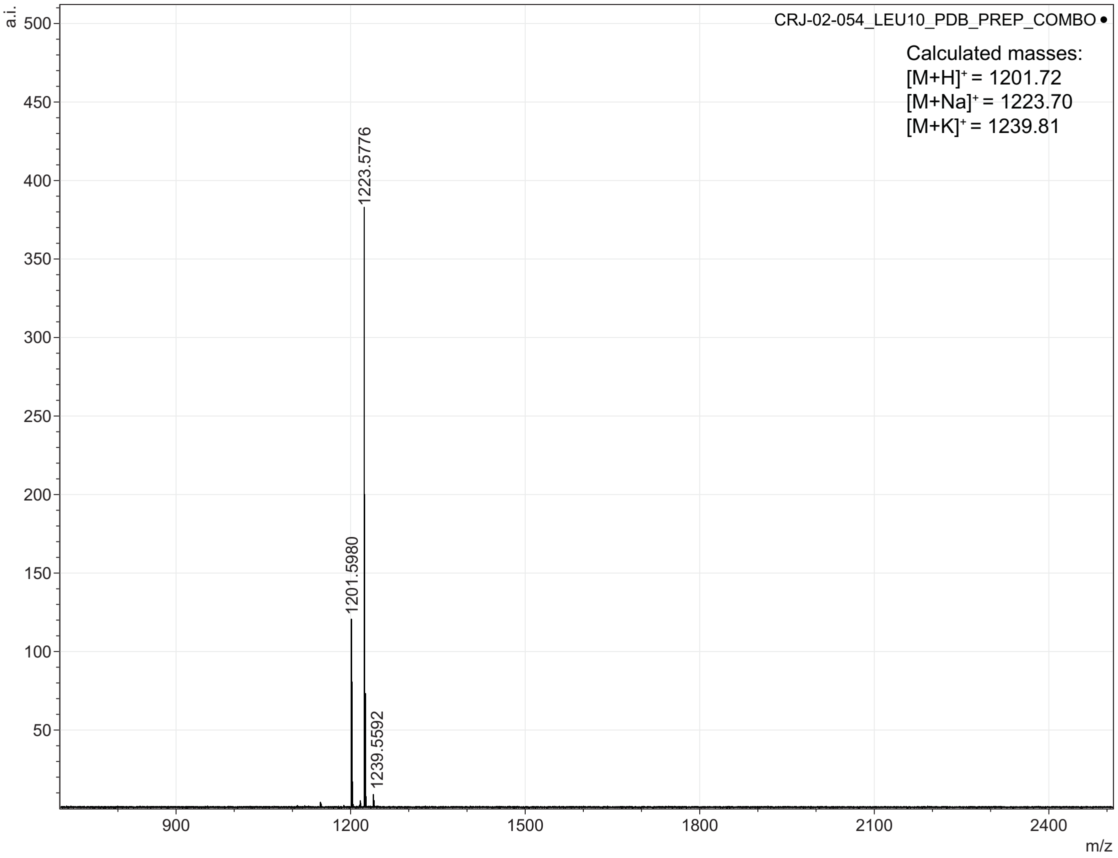
a.i.
CRJ-02-054_LEU10_PDB_PREP_COMBO
Calculated masses:
[M+H]+ = 1201.72
[M+Na]+ = 1223.70
[M+K]+ = 1239.81
1223.5776
1201.5980
1239.5592
m/z

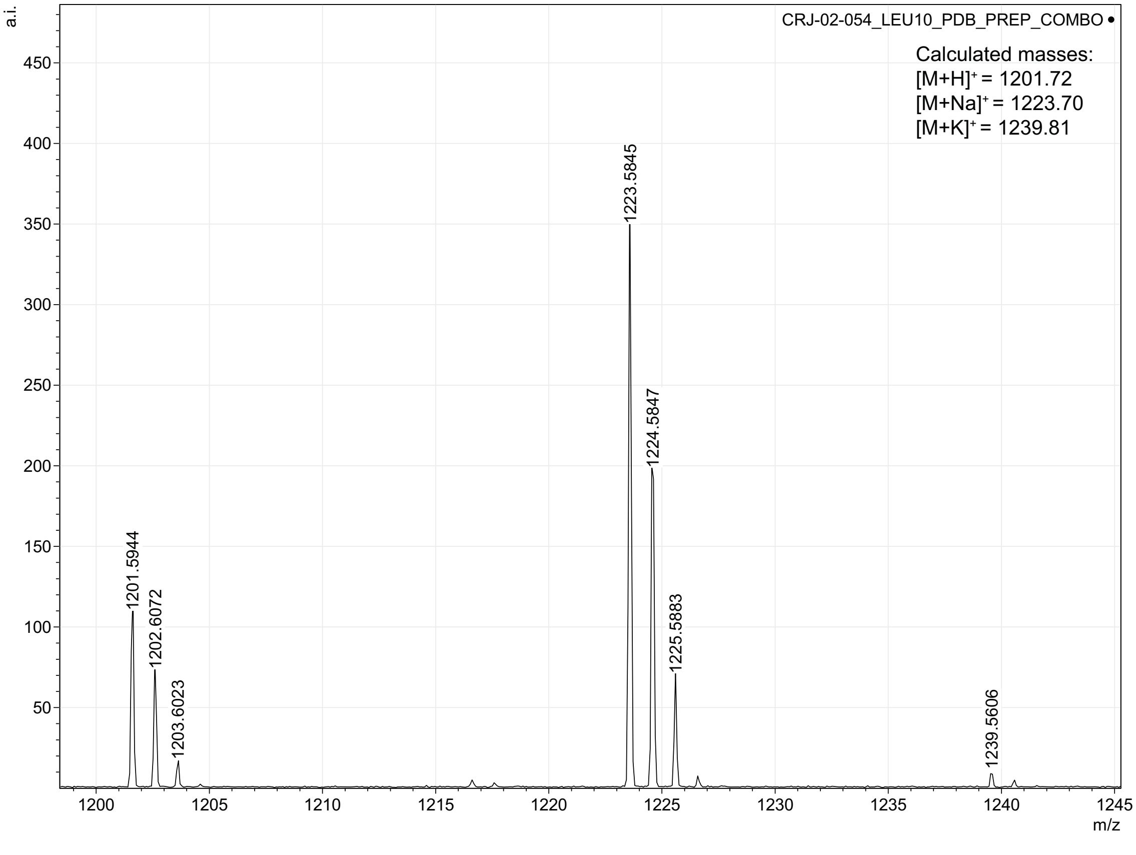
a.i.
CRJ-02-054_LEU10_PDB_PREP_COMBO
Calculated masses:
[M+H]+ = 1201.72
[M+Na]+ = 1223.70
[M+K]+ = 1239.81
1223.5845
1224.5847
1225.5883
1201.5944
1202.6072
1203.6023
1239.5606
m/z

Leu$_{10}$-teixobactin Prodrug C Analytical HPLC Trace and MALDI-TOF

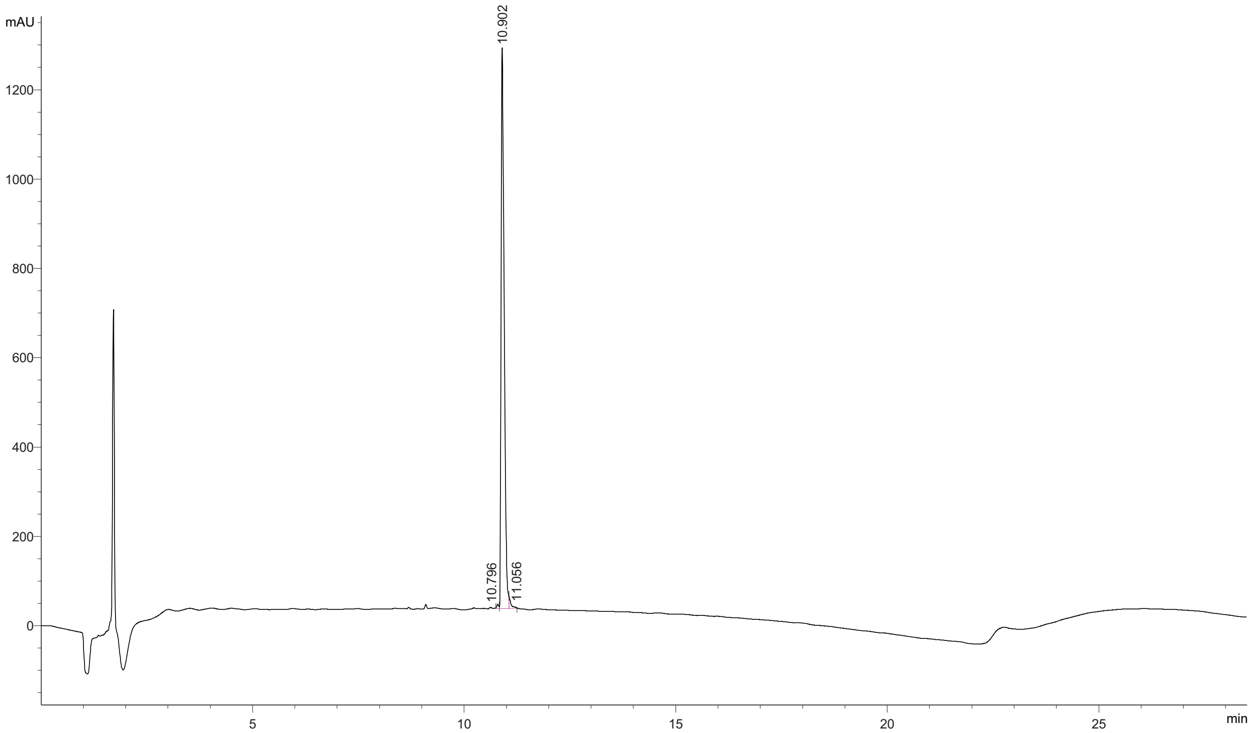

Peak #	RetTime [min]	Type	Width [min]	Area [mAU*s]	Height [mAU]	Area %
1	10.796	MF	0.0560	33.51337	9.98297	0.5203
2	10.902	MF	0.0836	6319.58105	1259.25037	98.1043
3	11.056	FM	0.0524	88.60129	28.16475	1.3754

Totals : 6441.69571 1297.39809

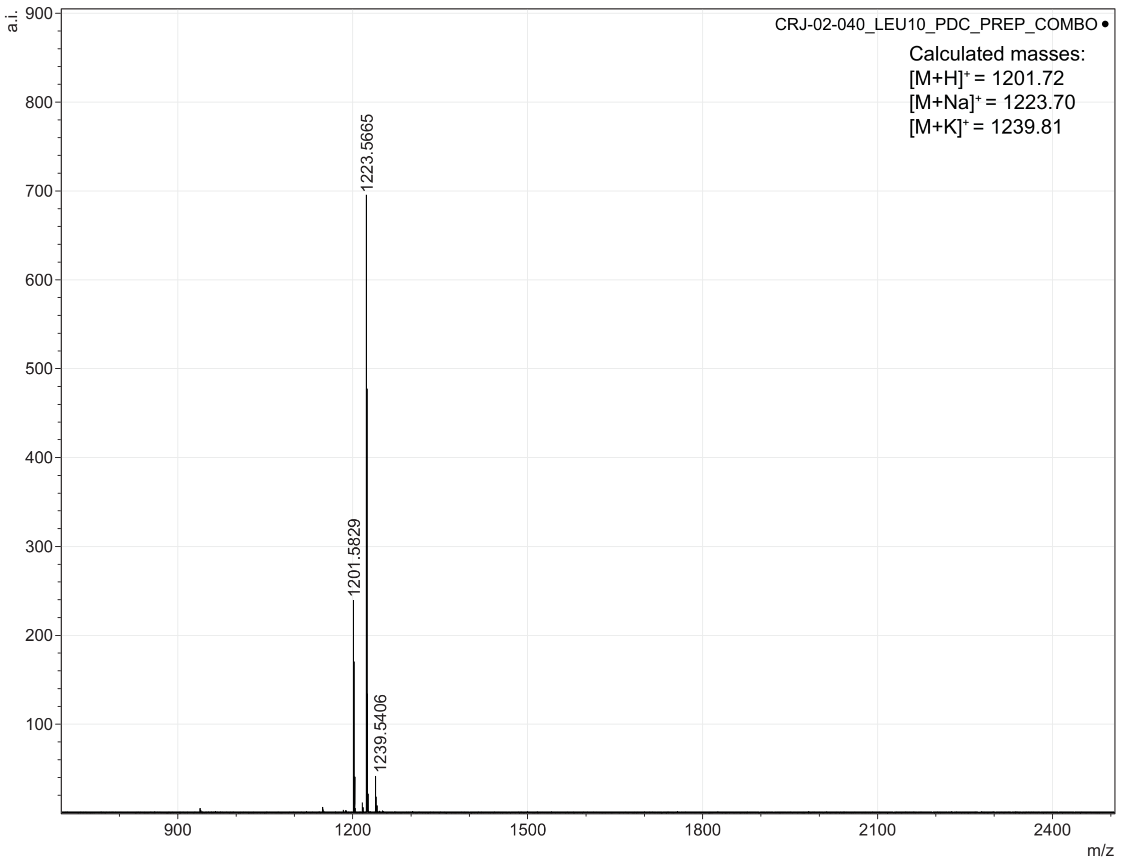
CRJ-02-040_LEU10_PDC_PREP_COMBO
Calculated masses:
[M+H]+ = 1201.72
[M+Na]+ = 1223.70
[M+K]+ = 1239.81
1223.5665
1201.5829
1239.5406
a.i.
m/z

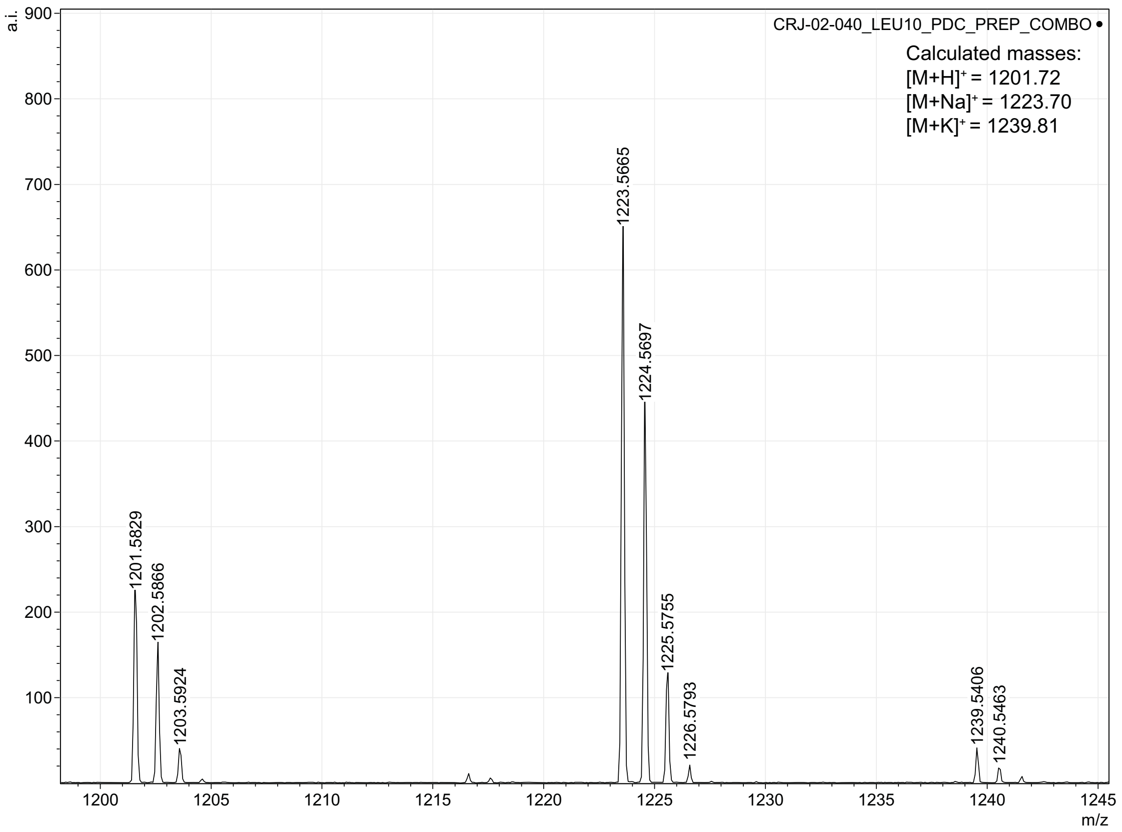
CRJ-02-040_LEU10_PDC_PREP_COMBO
Calculated masses:
[M+H]+ = 1201.72
[M+Na]+ = 1223.70
[M+K]+ = 1239.81
1223.5665
1224.5697
1225.5755
1226.5793
1201.5829
1202.5866
1203.5924
1239.5406
1240.5463
a.i.
m/z
